# Combined Effects of Metals, PCBs, Dioxins, and Furans on Cardiovascular Dysfunction

**DOI:** 10.3390/jox15030094

**Published:** 2025-06-19

**Authors:** Bolanle Akinyemi, Emmanuel Obeng-Gyasi

**Affiliations:** 1 Department of Built Environment, North Carolina A&T State University, Greensboro, NC 27411, USA; 2 Environmental Health and Disease Laboratory, North Carolina A&T State University, Greensboro, NC 27411, USA

**Keywords:** environmental pollutants, cardiovascular disease, heavy metals, polychlorinated biphenyls (PCBs), Bayesian Kernel Machine Regression (BKMR)

## Abstract

Environmental exposures to heavy metals, polychlorinated biphenyls (PCBs), dioxins, and furans have been associated with adverse cardiovascular outcomes, yet their combined effects remain underexplored. This study examined the joint influence of these contaminants on cardiovascular risk indicators in a representative sample of U.S. adults from the 2003–2004 National Health and Nutrition Examination Survey (NHANES). Biomarkers of exposure included lead, cadmium, mercury, twelve PCB congeners, seven dioxins, and ten furans. Cardiovascular outcomes were assessed using blood pressure, Framingham Risk Score (FRS), and lipid profiles. Associations were analyzed using multivariable linear regression and Bayesian Kernel Machine Regression (BKMR), adjusting for age, sex, ethnicity, body mass index, smoking, alcohol consumption, and income. The results demonstrated that metals, particularly mercury, were strongly associated with increased blood pressure and altered HDL cholesterol. PCBs were predominantly linked to elevated systolic blood pressure and FRS, with PCB156 and PCB126 identified as principal contributors. Furans exhibited the strongest associations with dyslipidemia, including elevated LDL cholesterol, total cholesterol, and triglycerides. Combined exposure analysis revealed a complex pattern, with increasing pollutant burdens associated with rising blood pressure and risk scores but declining lipid levels. These findings underscore the outcome-specific effects of pollutant mixtures and suggest that chronic low-level exposure to multiple environmental contaminants may contribute to cardiovascular dysfunction in the general population. Further longitudinal research is needed to confirm these associations and guide risk reduction strategies.

## 1. Introduction

Cardiovascular dysfunction (CVD) is a collective term that refers to the impaired ability of the cardiovascular system, including the heart and vascular system, to deliver blood throughout the body [1,2]. It is recognized as the leading non-communicable cause of death globally, with particularly profound effects in the United States, with 931,578 total deaths in 2021 [1]. The broad classification of CVD includes various conditions such as coronary artery disease, cerebrovascular disease, deep vein thrombosis, hypertension, and atherosclerosis, among others [2,3,4,5].

CVD is a complex and multifactorial health condition influenced by a combination of genetic, behavioral, and environmental factors [6]. Lifestyle choices such as particularly poor diet, physical inactivity, tobacco use, and excessive alcohol consumption are major modifiable risk factors contributing to the development of CVD [7]. These behaviors often interact synergistically with biological conditions such as hypertension, hyperlipidemia, obesity, and diabetes, amplifying the overall risk [8].

Additionally, behavioral and psychosocial factors such as chronic stress, poor sleep quality [9], and low socioeconomic status have been shown to influence cardiovascular outcomes, either directly or through unhealthy coping mechanisms (e.g., smoking or overeating) [10,11]. Genetics also plays a significant role, with heritable traits influencing lipid metabolism, blood pressure regulation, and inflammatory pathways [12]. Advances in genomics have identified numerous gene variants associated with CVD, though their expression is often modulated by environmental exposures and lifestyle factors [13,14,15].

Moreover, poor dietary choices such as diets high in saturated fats, sugars, and sodium are strongly linked to hypertension, obesity, and dyslipidemia, all of which are precursors to CVD [16,17,18]. Physical inactivity further exacerbates these risks by contributing to insulin resistance and endothelial dysfunction [19,20]. Smoking, another key behavioral factor, induces oxidative stress and inflammation, damages the vascular endothelium, and accelerates the progression of atherosclerosis [21,22]. Chronic stress, poor sleep, and substance abuse are additional behavioral influences that indirectly heighten cardiovascular risk by disrupting hormonal balance and promoting maladaptive coping strategies [14,23].

Genetic variations can influence lipid metabolism, blood pressure regulation, and the inflammatory response [24]. For example, polymorphisms in genes such as APOE, LDLR, and PCSK9 are known to affect cholesterol levels and increase the risk of coronary artery disease [13,25]. However, the phenotypic expression of these genetic traits is often modified by environmental and lifestyle factors, highlighting the importance of gene-to-environment interactions [26].

One of the challenges in combating CVDs is that it is frequently asymptomatic in early stages, leading to sudden and severe complications if not promptly detected [27,28]. Thus, regular screening and monitoring, especially for individuals with known risk factors, are paramount for early detection and effective management [29].

Environmental pollutants, both natural and anthropogenic, significantly contribute to health issues, including cardiovascular dysfunction [30,31]. The major sources of these pollutants include industrialization, transportation, and improper disposal of hazardous materials [31,32].

This involves activities such as industrialization, transportation, energy production, agricultural practices, waste disposal and landfills, deforestation and land-use change, and urbanization and construction, which initiate the release of contaminants such as toxic chemicals, pharmaceuticals, dyes, microplastics, polyaromatic hydrocarbons, toxic organic compounds, persistent organic pollutants POPs (like dioxin, polychlorinated biphenyls PCBs) [33], and heavy metals (such as lead, cadmium, mercury, etc.) into the environment [21,32].

Heavy metals such as lead (Pb), cadmium (Cd), and mercury (Hg) are notable environmental and public health concerns due to their toxicity and persistence in the environment [34,35,36,37]. Historically, Lead was a key component in various industries, but its toxicity to humans has made it a significant environmental and public health concern. It accumulates in the body over time, affecting multiple organ systems, especially the cardiovascular, neurological, renal, and hematological systems [34]. Lead exposure, even at low levels, has been associated with various adverse cardiovascular outcomes such as hypertension, atherosclerosis, arrhythmia, heart failure, and increased mortality rate [38]. The toxic effects of lead vary depending on the level and duration of exposure [39]. Chronic exposure to low levels of lead can have cumulative effects on systems such as the cardiovascular, neurological, renal, hematological, and reproductive systems, particularly in vulnerable populations such as children and pregnant women [40]. The primary exposure pathway for children is the ingestion or inhalation of lead-contaminated paint chips and dust, typically from deteriorating lead-based paints [41]. For adults, the elevated exposures are most associated with occupational settings [42].

Cadmium is a highly toxic heavy metal that is naturally present in the Earth’s crust but has become a significant environmental and occupational hazard due to human activities, including industrial processes, tobacco smoke, air pollution from burning fossil fuels, improper disposal of batteries, and water contamination resulting from these activities [35]. Cadmium is particularly concerning because of its long biological half-life, as it can persist in the body for decades, accumulating in vital organs such as the kidneys, liver, and bones [36]. Cadmium toxicity involves multiple mechanisms, including oxidative stress, inflammation, disruption of homeostasis, mitochondrial dysfunction, and cellular apoptosis; furthermore, exposure to cadmium is linked to hypertension, endothelial dysfunction, and heart failure [36]. Occupational exposure remains the most prevalent source of elevated cadmium levels, particularly in industries such as battery manufacturing, metal smelting, and welding [43,44].

Mercury is a naturally occurring heavy metal that poses significant health risks to humans and the environment [45]. While it exists in three various forms, elemental, inorganic, and organic mercury [37], all forms are toxic and can have detrimental effects on multiple organ systems, especially in developing fetuses and children. Historically, mercury was used in a variety of products and industrial processes; however, awareness of its toxicity has led to significant regulatory efforts aimed at limiting human exposure [37]. However, mercury pollution remains a global concern due to its persistence in the environment and its ability to bioaccumulate in the food chain, particularly in fish and seafood [46,47,48]. There are uncertainties surrounding the levels of methylmercury exposure resulting from fish consumption, as well as the potential health effects associated with this exposure [48]. Mercury exposure is associated with hypertension, atherosclerosis, and myocardial infarction [49]. Mercury’s toxicity can affect several organ systems, depending on the form of mercury, the route of exposure, and the dose [50,51]. Either chronic or low exposure levels can lead to cumulative damage over time, while acute exposure to high levels can result in immediate and severe health effects through mechanisms such as neurotoxicity, oxidative stress, inhibition of selenium-dependent enzymes, disruption of cellular homeostasis, and immune system dysregulation [52].

Polychlorinated biphenyls (PCBs) are a group of synthetic organic chemicals comprising 209 individual compounds, known as congeners, each with varying degrees of chlorination [53,54]. Despite being known for their non-flammability, chemical stability, insulation, and other useful properties, PCBs have become one of the most notorious environmental pollutants due to their persistence and their ability to bioaccumulate in living organisms, posing long-term health risks [55]. Exposure to PCBs affects the immune, endocrine, reproductive, nervous, and cardiovascular systems [54,56].

Dioxins are a group of chemically related compounds, such as polychlorinated dibenzo-p-dioxins (PCDDs), polychlorinated dibenzofurans (PCDFs), and some PCBs that exhibit similar toxic properties and are persistent environmental pollutants [57,58]. These highly toxic compounds are byproducts of various industrial processes, including waste incineration, chemical manufacturing, and the chlorine bleaching of paper [59]. Dioxins accumulate in fatty tissues due to their lipophilic nature, similar to PCBs, and can have severe health effects on humans, including carcinogenic and non-carcinogenic impacts like atherosclerosis, hypertension, diabetes, and disruptions to the nervous, immune, reproductive, and endocrine systems [58,60].

Furans are five-membered heterocyclic compounds with four carbon and one oxygen atom. They have garnered significant attention in medicinal chemistry and food science. They occur naturally in some products and are also utilized in the synthesis of various organic substances, including herbicides, pharmaceuticals, and plastics [61,62]. Furan derivatives exhibit diverse therapeutic applications [63], however, furan is also a potential food contaminant formed during thermal processing, classified as a possible human carcinogen by the International Agency for Research on Cancer [64]. Its formation in foods occurs through multiple pathways, including thermal degradation of carbohydrates, amino acids, and oxidation of ascorbic acid and polyunsaturated fatty acids [64]. Furans contribute significantly to food flavor and are widely present in thermally processed foods [62,65].

The simultaneous exposure to metals, PCBs, dioxins, and furans raises significant health concerns, as these pollutants can interact in ways that may amplify their toxic effects [66]. This concept refers to chemical interactions such as synergy, additivity, and antagonism, meaning that the combined health impacts of these chemicals may be more severe, equivalent to, or less than the effects of individual contaminants alone [67]. For instance, metals such as lead, cadmium, and mercury are known to generate reactive oxygen species (ROS) and induce oxidative stress [68]. When combined with PCBs and dioxins, which also generate oxidative stress and inflammatory responses, the compounded effect may lead to cellular damage which can accelerate inflammatory diseases, thereby increasing the risk of cardiovascular dysfunction and respiratory conditions [68,69].

Investigating the joint effects of metals, PCBs, dioxins, and furans on cardiovascular health is crucial for accurate health risk assessments, as current exposure standards may not fully capture the risks of simultaneous exposure to multiple contaminants [70].

Research into the combined effects of these pollutants is particularly relevant for developing public health policies. It can also inform clinical guidelines for diagnosing and treating conditions influenced by environmental pollutants, particularly in populations at high risk due to geographic or occupational exposure [71]. Recognizing the joint impacts can help in establishing more comprehensive strategies to limit exposure, improve detoxification treatments, and protect vulnerable populations. When individuals with high genetic susceptibility or unhealthy lifestyle practices are exposed to environmental pollutants, the resulting cardiovascular impact can be amplified, leading to higher morbidity and mortality. The objective of this study is to investigate the combined effects of metals (lead, cadmium, and mercury), PCBs, and dioxins on cardiovascular dysfunction. We hypothesize that the chemical mixtures studied may affect cardiovascular health through additional pathways, including endocrine disruption, mitochondrial dysfunction, and epigenetic modifications. Endocrine disruptors (PCBs, dioxins, furans) may interfere with hormonal signaling critical for vascular function [72,73]. Mitochondrial dysfunction induced by heavy metals (cadmium, mercury) can impair cardiac energetics and trigger cardiomyocyte apoptosis [74,75]. Additionally, epigenetic alterations caused by POP might alter gene expression patterns related to lipid metabolism, inflammation, and endothelial integrity, contributing cumulatively to CAD [76,77].

## 2. Materials and Methods

### 2.1. Data Source

The data used in the study were extracted from the 2003–2004 National Health and Nutrition Examination Survey (NHANES) database. NHANES offers publicly available data on the health and nutrition of the US civilian, non-institutionalized population. The NHANES program uses a cross-sectional health examination survey and employs a multi-stage, stratified probability sample of the participants. The data were collected by the United States Centers for Disease Control and Prevention (CDC) after obtaining informed consent from the participants [78].

### 2.2. Data Collection

The CDC publishes the NHANES dataset in two-year cycles, which includes interviews conducted in the homes of individuals of different ages. The consenting individuals are required to complete the health examination component of the survey. Most health examinations were carried out in the mobile examination centers (MEC), which provide a standardized setting for collecting high-quality data. Further details can be found on data collection, and the methodologies and processes are available on the CDC’s NHANES website [78].

### 2.3. Study Population

NHANES 2003–2004 data include over-sampling of White, African American, Mexican American, and other ethnic groups. The study design also includes a representative sample of these groups by age, gender, and income level. Each eligible participant signed a consent form. This consent form permits the collection and storage of a small sample of blood and urine for future specimen testing [78]. The database contains data for 10,122 individuals, collected between January 2003 and December 2004. Our study utilized data from 432 individuals, all of whom had complete data for each biomarker. For this study, individuals between the ages of 20 and 39 comprised 41.20 percent of the sample. Those between 40 and 59 were 29.40 percent, and those who were 60 and above were 29.40 percent.

### 2.4. Exposure Variables

The key exposure variables of this study were divided into three parts, metals (lead, cadmium, and mercury (blood total mercury)), PCBs (out of the 209 congeners of PCBs, we worked with 12 congeners), dioxins (7), and furans (10), as detailed in Appendix A (check Appendix A for the chemical name of PCBs, dioxins and furans).

Exposure to the combined mixture of 32 environmental chemicals including metals (e.g., lead, cadmium, mercury), PCBs, dioxins, and furans is of particular concern due to their persistence, bioaccumulation, and established or suspected cardiotoxic effects. These substances are often co-occurring in human biological matrices and environmental media, leading to chronic, low-level exposure through multiple routes [79]. The selection of this mixture reflects a known overlap in sources and toxicological profiles, as well as evidence from previous studies demonstrating their joint presence in the general population [80,81,82].

Relevant exposure pathways vary by compound class but broadly include dietary intake (particularly through the consumption of contaminated fish, meat, dairy, and processed foods) [83,84], inhalation of airborne particulates (especially near industrial sources or waste incinerators) [85], ingestion of contaminated soil or dust (notably for metals like lead and arsenic) [86,87], and occupational exposures (e.g., among workers in manufacturing, construction, waste management, and agriculture) [88,89]. Environmental contamination from historical industrial activity, legacy pollutants, and ongoing emissions continues to affect residential areas, particularly those in close proximity to industrial zones, highways, or older housing stock [90,91].

This mixture represents real-world exposures experienced by diverse populations, with cumulative risk arising from both shared and distinct pathways. Importantly, the inclusion of these 32 chemicals in the present study is supported by national biomonitoring data (NHANES) indicating frequent co-detection, thus reinforcing the ecological validity of the selected mixture for cardiovascular health research [92,93].

#### Exposure Assessment

Exposure biomarkers were measured using blood and serum samples collected during the NHANES 2003–2004 examination cycle [78]. Whole blood was used to assess levels of metals such as lead, cadmium, and total mercury, using inductively coupled plasma–dynamic reaction cell mass spectrometry (ICP-DRC-MS). The analyses were conducted with a PerkinElmer ELAN DRC-II ICP-MS system (PerkinElmer, Waltham, MA, USA), equipped with a Meinhard nebulizer, a cyclonic spray chamber, and an AS-93 autosampler [94]. Only total blood mercury was available for this cycle, encompassing both inorganic and methylmercury species [95]. POPs such as PCBs, dioxins, and furans, were quantified in serum using high-resolution gas chromatography with isotope-dilution high-resolution mass spectrometry (HRGC/ID-HRMS). This was performed using a Hewlett-Packard 6890 GC system (Agilent Technologies, Santa Clara, CA, USA) equipped with a DB-5ms capillary column (30 m × 0.25 mm i.d., 0.25 µm film thickness), interfaced with a Waters Autospec Premier high-resolution magnetic sector mass spectrometer (Waters Corporation, Milford, MA, USA) [96]. These compounds were lipid-adjusted based on serum triglyceride and cholesterol concentrations, reflecting their lipophilic properties and long biological half-lives [97]. The use of matrix-specific adjustments aligns with known toxicokinetic characteristics of each chemical class, improving the accuracy of exposure assessment and relevance to long-term cardiovascular risk [98,99].

### 2.5. Cardiovascular Disease Outcome Variables

The assessment of cardiovascular disease risk was determined through seven cardiovascular related indicators: diastolic blood pressure—DBP, systolic blood pressure—SBP, Framingham risk score—FRS, low density lipoprotein–LDL cholesterol, high density lipoprotein–HDL cholesterol, total cholesterol—TC, and triglycerides, along with relevant covariates [100,101]. These biomarkers were chosen based on their established association with cardiovascular health outcomes [102]. The models were adjusted for relevant covariates, including age, sex, ethnicity, BMI, smoking status, alcohol consumption, and income [103].

#### Calculating the Framingham Risk Score (FRS)

The FRS is a widely used indicator to estimate an individual’s 10-year risk of developing CVD [104]. The FRS helps clinicians assess primary prevention strategies by estimating the probability of coronary events, such as myocardial infarction (heart attack) and coronary heart disease-related death. The FRS is based on multiple key risk factors such as age, TC, HDL cholesterol, SBP, smoking status, diabetes, and hypertension treatments. Each risk factor is assigned a point value based on pre-defined scoring tables. The sum of these points is mapped to a 10-year CVD risk percentage [104,105]. The FRS was developed as done in previous studies [106].

### 2.6. Statistical Analytics

#### 2.6.1. Descriptive Analysis

Descriptive statistics were used to summarize the distribution of the exposures, demographic variables (age, sex, ethnicity, income, smoking status, alcohol consumption, and body mass index (BMI)), and CVD-related outcome variables. The mean and standard deviation were determined for continuous variables, while percentages were determined for categorical variables. Correlation analyses were then used to examine relationships between the exposure variables and each outcome variable [107]. Spearman’s rank correlation was used to account for potential non-linear associations through matrix and heatmap visualization for the outcome variables [108]. The Spearman rank correlation is mathematically represented as follows:

$$rs=\frac{1-6\sum d_{i}^{2}}{n\left(n^{2}-1\right)}$$

where *d_i_* is the difference between the ranks of the *i-th* pair, and *n* is the number of observations of the dataset.

The Spearman rank (*rs*) value ranges from −1 to +1, with a positive value indicating a positive relationship between the variables, while a negative value indicates a negative relationship, and when *rs* = 0, this indicates no association between the two variables [109,110].

#### 2.6.2. Linear Regression Methods

Multiple linear regressions were used to determine the association between the combined effects of metals (lead, cadmium, and mercury), PCBs, dioxins, and furans on cardiovascular disease makers. The response variable (*Y*) was the CVD-related outcome markers. All the regression models were adjusted for the demographic variables (age, sex, ethnicity, income, smoking status, alcohol consumption, and BMI) and the *p*-values were calculated. The corresponding 95% confidence intervals were obtained for each fitted model. The linear regression equation was as follows:

$$\begin{array}{l} Y=\beta 0+\beta 1Leadi+\beta 2Cadmiumi+\beta 3Mercuryi+\beta 4Age\\ \;\;\;\;\;\;\;\;+\beta 5Gender+\beta 6\;Income+\beta 7\;Ethnicity+\beta 8\;Smoking\\ \;\;\;\;\;\;\;\;+\beta 9BMI+\beta 10\;Alcohol+\varepsilon i \end{array}$$

where *Y* represents different responses such as FRS, DBP, SBP, HDL cholesterol, LDL cholesterol, TC and triglycerides, and εi ~ N(0, σ2)  is the random error term.

#### 2.6.3. Bayesian Kernel Machine Regression BKMR

Bayesian kernel machine regression (BKMR) was implemented to evaluate the combined effects of multiple exposures on cardiovascular-disease-related outcomes [105]. BKMR is a method that has been widely used to evaluate the combined effect of multiple exposures on a wide range of health outcomes, due to its capacity to model non-linear and non-additive relationships effectively. The BKMR model provided posterior inclusion probabilities (PIPs) to evaluate the contribution of various exposures to the cardiovascular outcomes examined in our study [105]. These outcomes are DBP, SBP, LDL cholesterol, HDL cholesterol, TC, and triglycerides. PIPs are a statistical measure that quantifies the likelihood of an exposure being a significant predictor while accounting for interactions among different exposures. This method enables us to determine the significance of each metal in influencing specific cardiovascular outcomes. For each variable  i=1,…,n we assume the following:

$$Y_{i}=h\left(z_{i}\right)+x_{i}^{T}\beta +\epsilon_{i}$$

where $Y_{i}$ is a health endpoint (cardiovascular dysfunction), $z_{i}={\left(z_{i}1,\;\ldots ,\;z_{i}M\right)}^{T}\;$ is a vector of *M* exposure variables (e.g., metals, PCBs, dioxins, and furans), $x_{i}$ contains the potential cofounders set, and ${\in_{i}}_{∼\;}^{i.i.d}N\left(0,\;\sigma^{2}\right).$ h(.) characterizes the high dimensional exposure–response function that may be incorporated in the non-linearity interaction between the mixture components [105].

We constructed a univariate exposure–response function to determine how a single variable (exposure) affects each outcome (response) while keeping all other variables constant. Next, we determined the overall effect of all exposures on the health outcome at different increasing quantiles from 25% to 75%. We moved on to construct the single-exposure effects at 95% credible intervals to identify the change in response that is associated with the change in single exposure from its 25th to 75th quantile while all other exposures are fixed at a specific quantile (25th, 50th, or 75th). Then, we determined the single-variable interaction of each metal, PCBs, dioxins, and furans, comparing the effect of each exposure when all other exposures are fixed.

All analysis was adjusted for age, sex, ethnicity, income, smoking status, alcohol consumption, and body mass index (BMI). We utilized R (version 4.4.2; R Foundation for Statistical Computing, Vienna, Austria) for all analyses, with a significance level of 0.05. We utilized a suite of specialized R packages to support advanced modeling, data processing, and visualization. The BKMR package was central to our analysis, enabling the implementation of an assessment to evaluate the joint effects of multiple environmental exposures [105].

## 3. Results

### 3.1. Characteristics of the Sample Population

Table 1 assesses the characteristics of the 432-sample population, demonstrating a diverse population with an overall mean age of 46.3 years (S.D. = 17.2). Men had a slightly higher mean age (47.6 years, S.D. = 17.5) compared to women (45.2 years, S.D. = 16.8). The overall average BMI was 28.3 (S.D. = 6.1), with women showing a marginally higher mean BMI (28.6, S.D. = 6.7) than men (28.0, S.D. = 5.3).

In terms of income levels, the largest proportion of the population fell within the range of $25,000 to $44,999, with 24.31% (Table 1), followed by those in the category $75,000 and above, with 22.22%. The ethnicity distribution showed a dominance of Mexican Americans (57.17%), with Non-Hispanic Whites (19.21%), Other Hispanics (16.9%), Non-Hispanic Blacks (3.24%), and Other Races (3.47%) making up smaller proportions (check Table 1 for further details).

Smoking status indicated that 29.40% were non-smokers and 18.98% were smokers. Alcohol consumption data showed that a large majority (68.5%) consumed alcohol, while 25.7% abstained.

### 3.2. Summary Statistics of Outcome Variables by Gender

Supplementary Table S1 assesses cardiovascular and lipid profile measures stratified by gender. Overall mean for SBP was 122.8 mmHg (S.D. = 19.9), with men showing higher levels (125.0 mmHg, S.D. = 18.6) compared to women (120.9 mmHg, S.D. = 20.8). DBP followed a similar pattern, with men again demonstrating slightly higher averages (71.6 mmHg, S.D. = 14.1) than women (69.4 mmHg, S.D. = 10.8).

Lipid profiles revealed important gender differences as women exhibited higher mean HDL cholesterol levels (59.7 mg/dL, S.D = 17.6) compared to men (48.6 mg/dL, S.D = 11.8). However, LDL cholesterol was notably higher in men at (122.4 mg/dL, S.D = 35.8) than in women (111.7 mg/dL, S.D = 36.6). Total cholesterol and triglycerides showed relatively similar values between genders, averaging 199.3 mg/dL and 140.2 mg/dL, respectively.

**Figure 1:**
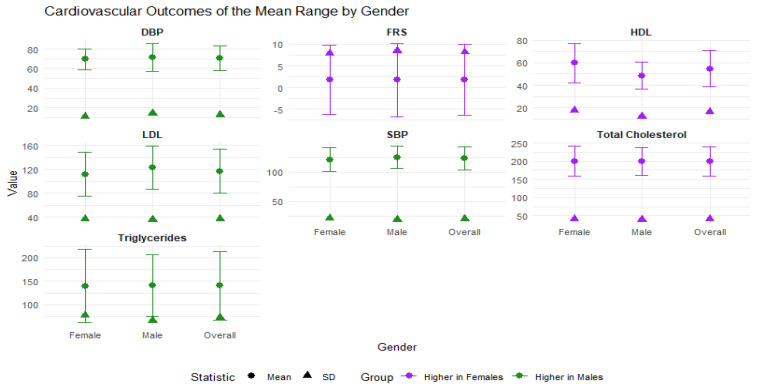
Dot-plot visualization of the descriptive statistics summary of cardiovascular risk factors according to highest mean value by gender.

Figure 1 visualizes the CVD risk factors, which include FRS, blood pressure indices, and lipid profiles. Out of the seven cardiovascular-related variables analyzed, four were lower in females and three were lower in males. The females had lower mean values for DBP, SBP, LDL cholesterol, and triglycerides. These indicators suggest that, on average, females may have a more favorable cardiovascular profile in terms of blood pressure and harmful lipid fractions. Conversely, males exhibited lower values for the FRS, HDL cholesterol, and total cholesterol. While a lower FRS suggests a better overall risk profile for males, their lower HDL (“good” cholesterol) may offset some of this benefit.

A similar grouping approach was applied for visualization in Supplementary Figure S1, with a total of 32 exposure variables analyzed and grouped accordingly. The first group includes 20 exposure variables in which females demonstrated lower mean concentrations than males. For metals and PCBs, the variables included cadmium, lead, PCB156, PCB157, PCB189, PCB81, and PCB169. Among dioxins, 1,2,3,7,8-Pentachlorodibenzo-p-dioxin (PeCDD), 1,2,3,4,7,8-Hexachlorodibenzo-p-dioxin (HxCDD), and 1,2,3,6,7,8-Hexachlorodibenzo-p-dioxin (HxCDD) showed lower levels in females. All 10 furans were predominantly lower in females, while mercury, PCB28, PCB66, PCB74, PCB105, PCB118, PCB167, PCB126, 1,2,3,7,8,9-Hexachlorodibenzo-p-dioxin (HxCDD), 1,2,3,4,6,7,8-Heptachlorodibenzo-p-dioxin (HpCDD), 1,2,3,4,6,7,8,9-Octachlorodibenzo-p-dioxin (OcDD), and 2,3,7,8-Tetrachlorodibenzo-p-dioxin (TcDD) were lower in males. This indicates that while females generally had lower exposures for most of these toxicants, there are notable exceptions where males exhibited a lower toxic burden.

### 3.3. Summary Statistics Mean (S.D) of Exposure Variables by Gender

Table 2 presents the unadjusted mean and standard deviation of the various pollutants, revealing diverse distributions. The descriptive statistics data illustrate gender differences within the population studied.

### 3.4. Spearman’s Correlation Analysis

**Figure 2:**
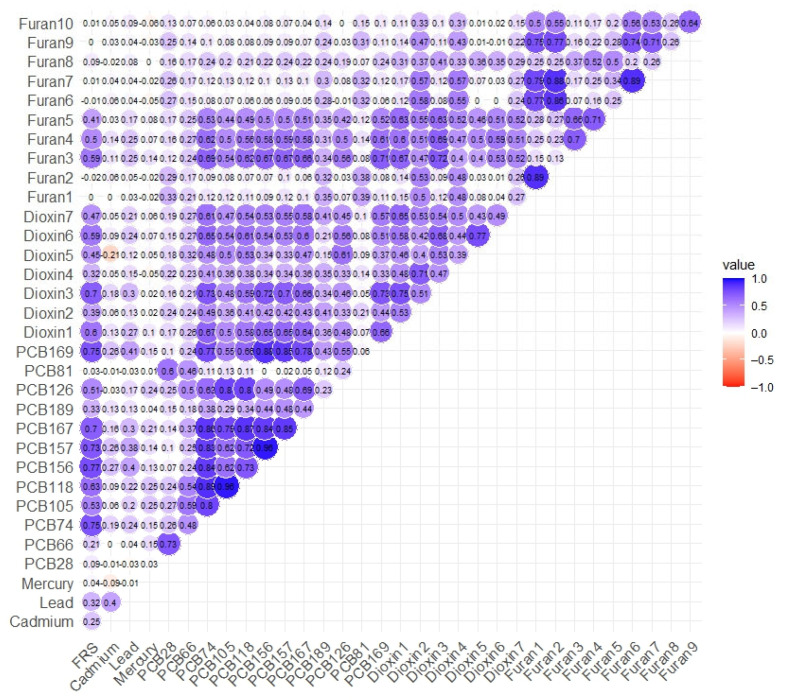
Spearman’s correlation between pairs of metals, PCBs, dioxins, and furans on FRS.

Figure 2 illustrates an unadjusted Spearman correlation analysis of each predictor and its correlation with the various biomarkers with FRS. The correlation ranges from 1 to −1, with 1 indicating a strong positive correlation and −1 indicating a strong negative correlation. It can also be visually identified by deep blue colors, indicating a strong correlation, and deep red colors indicating a negative correlation. Most of the predictors show a strong correlation with the FRS. The results reveal several significant associations between exposure to pollutants and the cardiovascular biomarkers, with the strongest positive correlation between FRS and PCB 156 at ≈0.8. Supplementary Figures S2–S7 display the plots for SBP, HDL cholesterol, LDL cholesterol, TC, and triglycerides.

### 3.5. Linear Regression Analysis

As shown in Table 3, linear regression revealed significant associations between multiple environmental exposures and cardiovascular or lipid outcomes, adjusting for demographics. Mercury, PCB118, and 1,2,3,7,8-Pentachlorodibenzofuran (PeCDF) were inversely associated with risk scores or lipid markers, while lead, select PCBs, and furans showed positive associations. DBP and SBP intercepts aligned with expected baseline values. Notably, OcDD, 1,2,3,4,7,8,9-Heptachlorodibenzofuran (HpCDF), and PCB105 were associated with changes in blood pressure and cholesterol, highlighting their potential cardiovascular relevance.

Mercury showed an inverse association with FRS, while age and gender remained strong positive predictors. Lead exhibited a moderate positive association with HDL, although the clinical implications are unclear. PCBs and dioxins had mixed effects: PCB 167 and HpCDF correlated with elevated SBP, while OcDD correlated with both DBP and SBP. PCB105, TCDF, and HxCDD were associated with higher HDL levels, whereas PCB118 and PeCDF were negatively associated, suggesting possible lipid dysregulation.

LDL and TC were inversely associated with PCB189, PCB169, and HpCDF, indicating potential non-monotonic or compensatory effects. Triglycerides were positively associated with PCB126 and PCB169, while HxCDF showed a negative association. These findings highlight the complex and heterogeneous impact of environmental mixtures on cardiovascular risk and lipid homeostasis [58].

### 3.6. BKMR Analysis

#### 3.6.1. Posterior Inclusion Probability (PIP)

Table 4 presents the PIPs from the BKMR analysis, which estimate the relative importance of each group of environmental markers (metals, PCBs, dioxins, and furans) in explaining variability in cardiovascular outcomes. Group-level PIPs indicate the contribution of each pollutant class, while conditional PIPs highlight key individual drivers within each group.

Metals were most relevant for DBP and HDL cholesterol, with mercury emerging as the primary contributor. PCBs had the highest PIPs for FRS and SBP, driven by PCB 156 and PCB 126, respectively. For LDL cholesterol, TC, and triglycerides, furans showed the highest relevance, with 2,3,7,8-Tetrachlorodibenzofuran (TCDF) contributing most to TC and 1,2,3,7,8-Pentachlorodibenzofuran (PeCDF) to LDL cholesterol and triglycerides.

#### 3.6.2. Univariate Exposure–Response Relationship

**Figure 3:**
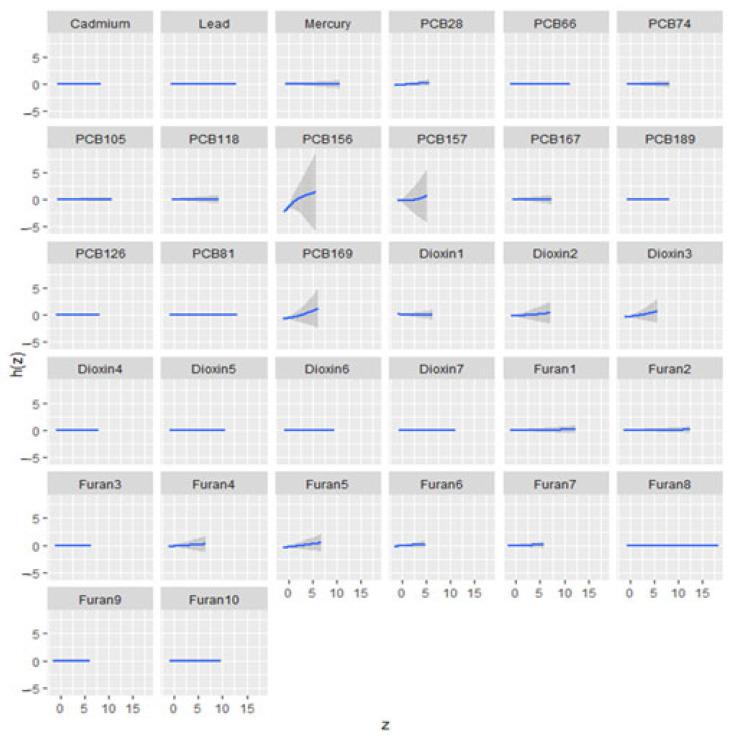
Univariate exposure–response functions and 95% credible interval of metals, PCBs, dioxins, and furans on CVD-related outcome: FRS. (Appendix A and SI for SBP, HDL cholesterol, LDL cholesterol, TC, triglycerides).

The univariate exposure–response relationship with a 95% credible interval is shown in Figure 3 for FRS. This indicates the effect of each pollutant in a mixture when all other pollutants are held at the median. Results indicated a non-linear relationship for PCB 156, and TC exhibited a relatively flat exposure–response relationship with most exposures; however, 2,3,7,8-Tetrachlorodibenzofuran (TCDF) displayed a non-linear exposure–response relationship, characterized by fluctuations. Lead showed a flat relationship with DBP, HDL, and triglycerides, while mercury showed a curved relationship with DBP and HDL. The results for SBP, DBP, LDL, HDL, TC, and triglycerides are found in Supplementary Figures S8–S13.

Adjusted for alcohol consumption, smoking status, age, ethnicity, income level, gender, and BMI.

#### 3.6.3. Overall Exposure Effect

**Figure 4:**
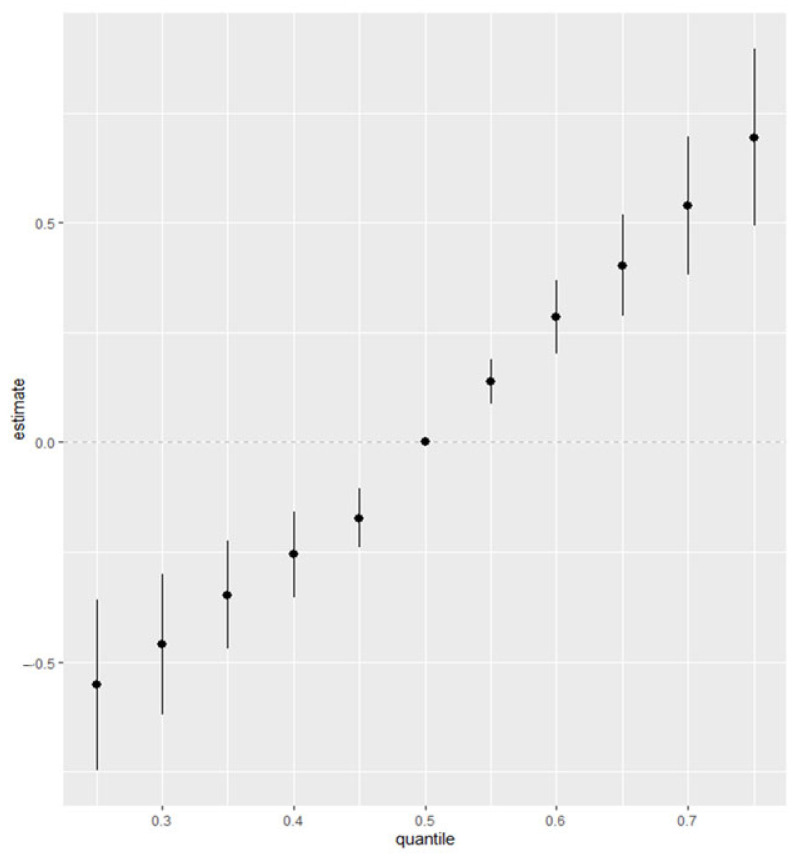
Overall exposure effects chart showing the increasing quantile of combined exposures from the 25th to the 75th quantile as compared to the 50th quantile for FRS. Adjusted for alcohol consumption, smoking status, age, ethnicity, income level, gender, and BMI.

Figure 4 illustrates the overall effect of exposure to all pollutants on FRS, comparing increasing quantiles from the 25th to the 75th percentile with the 50th percentile. FRS showed a positive relationship with small credible intervals, indicating the direction and certainty of the effect of all the exposures on FRS. Whereas DBP showed only a slight deviation from zero, with large credible intervals. Supplementary Figures S14–S19 show the results for other CVD-related markers.

#### 3.6.4. Single-Variable Effect

**Figure 5:**
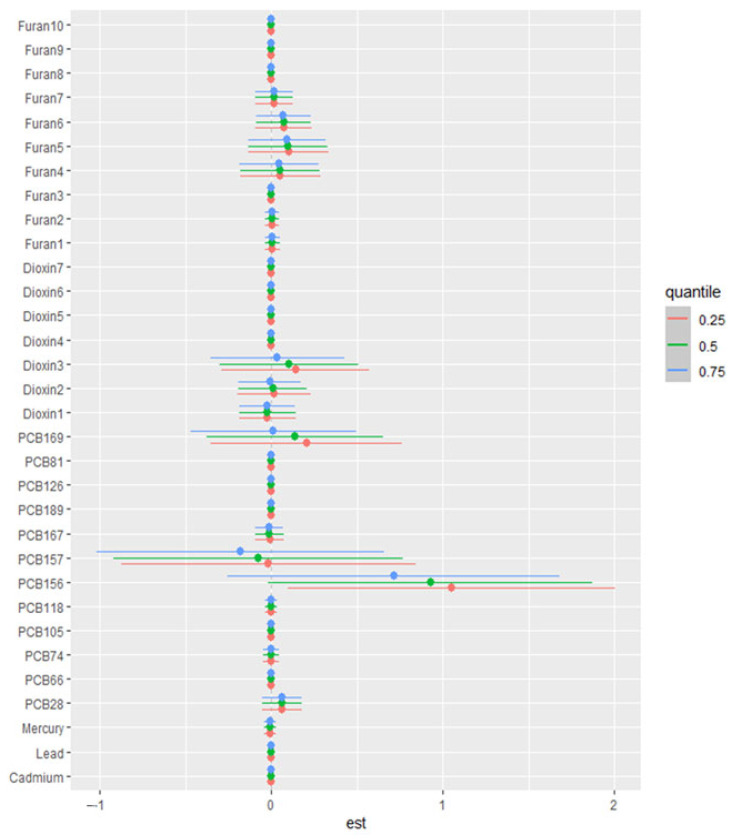
Single variable effect chart showing the single-exposure effect and 95% credible interval, defined as the change in response associated with a change in a single exposure from its 25th to 75th quantile while all other exposures are fixed at a specific quantile (25th, 50th, or 75th) for FRS. Adjusted for alcohol consumption, smoking status, age, ethnicity, income level, gender, and BMI.

Figure 5 presents the results of single-exposure effect analyses evaluating the associations between individual environmental exposures and key cardiovascular and FRS. Results for SBP, DBP, HDL, LDL, TC, and triglycerides are found in Supplementary Figures S20–S25.

**Figure 6:**
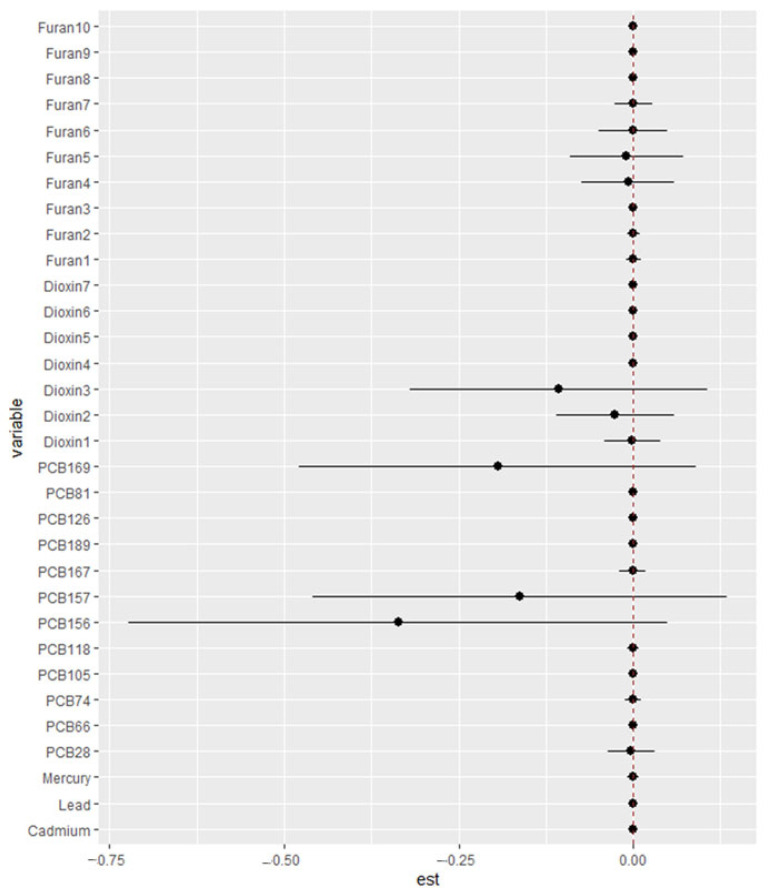
Single-variable interaction analyses for FRS assessing the effect of each environmental exposure (metals, PCBs, dioxins, and furans) from its 25th to 75th percentile, when all others are fixed at the 25th percentile compared to the 75th percentile. Adjusted for alcohol consumption, smoking status, age, ethnicity, income level, gender, and BMI.

A single-variable interaction analysis is demonstrated in Figure 6, illustrating the interaction between each pollutant and all the other pollutants. Results for SBP, DBP, HDL, LDL, TC, and triglycerides are found in Supplementary Figures S26–S31.

#### 3.6.5. Bivariate Exposure–Response Relationship

**Figure 7:**
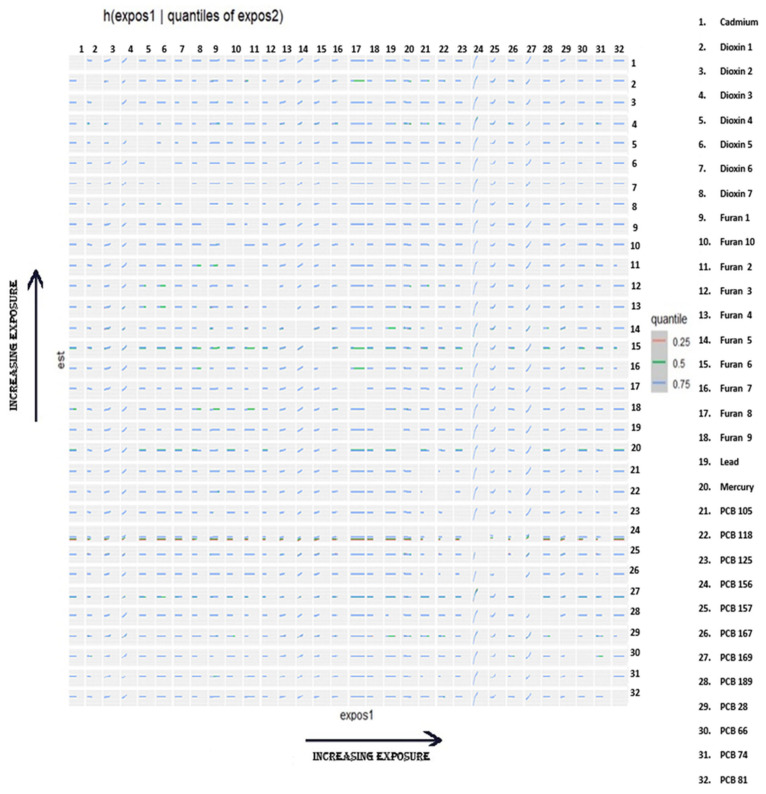
Bivariate exposure–response relationship for FRS. The bivariate exposure–response function illustrates the joint association of increasing exposure of metals, PCBs, dioxins, and furans (X-axis) as compared to increasing quantiles of a second exposure at the 0.25 (red), 0.5 (green), and 0.75 (blue) quantiles. Adjusted for alcohol consumption, smoking status, age, ethnicity, income level, gender, and BMI.

Figure 7 presents the bivariate exposure–response analysis of metals, PCBs, dioxins, and furans in relation to FRS. Each analysis assesses the effect of increasing levels of one exposure while holding a second exposure at varying quantiles (25th in red, 50th in green, and 75th in blue), with all other exposures maintained at their median values. The plots display exposure levels for the 32 measured toxicants along both axes. Each cell in the plot illustrates the joint association of two exposures on cardiovascular risk outcomes under the modeled conditions. Results for SBP, DBP, HDL, LDL, TC, and triglycerides are found in Supplementary Figures S32–S37.

## 4. Discussion

### 4.1. Overview and Descriptive Statistics Results

This study examined the combined effect of environmental pollutants such as heavy metals (lead, cadmium, mercury), PCBs, dioxins, furans, and several cardiovascular outcomes, including SBP, DBP, FRS, HDL cholesterol, LDL cholesterol, TC, and triglycerides.

The study population was mid-adult on average (mean age ~46 years) with a diverse ethnic makeup, predominantly Mexican American (57%). Men and women were roughly equal in number and showed notable differences in both cardiovascular measures and environmental exposures. Men had slightly higher mean in SBP and DBP than women (SBP 125.0 vs. 120.9 mmHg; DBP 71.6 vs. 69.4 mmHg). This matches the work of Reckelhoff and colleagues [111]. Conversely, women exhibited more favorable lipid profiles on average, with higher HDL cholesterol (59.7 vs. 48.6 mg/dL) and slightly lower LDL cholesterol (111.7 vs. 122.4 mg/dL) compared to men. These gender-based differences in CVD-related outcomes underscore the importance of adjusting for gender in the analysis. All subsequent models are controlled for age, gender, BMI, smoking, alcohol use, income, and ethnicity to account for such confounders.

Table 3 indicates that females generally had lower mean concentrations of many pollutants, though there were exceptions. For example, women had lower levels of cadmium and lead on average but had significantly higher levels of certain PCB congeners (e.g., PCB126) and a dioxin congener 1,2,3,4,6,7,8,9-Octachlorodibenzo-p-dioxin (OcDD) compared to men. These patterns suggest differences in exposure sources or toxicokinetics by gender, highlighting the need to consider it in all analyses [112]. Overall, the descriptive results establish a heterogeneous population with pronounced gender differences in cardiovascular risk factors and pollutant burdens, thereby justifying the need for multivariable analyses to disentangle relationships [113].

Given that approximately 75% of our sample is Hispanic, it is essential to examine the complex interplay of cultural, socioeconomic, and genetic factors within this demographic [114,115]. Socioeconomic factors, including access to healthcare and healthy foods, education, and occupational exposures, may exacerbate exposure and susceptibility to environmental pollutants [116,117]. Genetically, Hispanics may exhibit variations in genes related to lipid metabolism and inflammatory responses, which could potentially heighten their sensitivity to toxicants [118]. Understanding these culturally and genetically mediated susceptibilities enhances the interpretation of how environmental exposures influence cardiovascular health in this population subgroup [119,120].

### 4.2. Associations Between Pollutant Exposures and Cardiovascular Outcomes

#### BKMR Results

BKMR was used to assess the mixture effect as it effectively models non-linear effects and interactions. This allowed for the assessment of how metals, PCBs, dioxins, and furans collectively relate to cardiovascular dysfunction. Using advanced Bayesian modeling, the analysis identified which environmental exposures were most strongly associated with each cardiovascular-related outcome [105]. Overall, distinct pollutant classes emerged as key drivers for different health endpoints. The PIP analysis revealed that the metal exposure group (particularly mercury) showed the highest influence on DBP and HDL. In other words, higher metal burdens (notably mercury) were linked to higher DBP and unfavorable changes in HDL (a lower “good” cholesterol level), underscoring metals’ contribution to blood pressure elevation and dyslipidemia. By contrast, PCBs were the dominant exposures associated with elevated SBP and increased FRS. Within the PCB mixture, certain congeners stood out: PCB156 was a principal contributor to higher SBP, and PCB126 to higher FRS. These dioxin-like PCB congeners may exert hypertensive and pro-atherogenic effects that raise overall cardiovascular risk.

Meanwhile, dioxin and furan compounds were most strongly linked to adverse lipid profiles. Furans, in particular, were the leading group affecting LDL cholesterol, total cholesterol, and triglycerides. For example, 2,3,7,8-TCDF exposure was a key predictor of elevated total cholesterol levels, and 1,2,3,7,8-Pentachlorodibenzofuran (PeCDF) showed strong associations with higher LDL and triglycerides. The analysis of combined exposures further revealed that as the overall burden of these environmental chemicals increased (from lower quartile to upper quartile levels), there was a gradual rise in SBP, FRS, and even HDL, whereas an inverse trend was observed for LDL, total cholesterol, and triglycerides. This pattern suggests complex, outcome-specific effects: combined pollutant exposure tended to raise blood pressure and calculated risk scores, but interestingly was associated with lower total lipid levels, a finding that may reflect homeostatic or toxicity-related disruptions in lipid metabolism [121,122]. It is noteworthy that the FRS, which integrates age, blood pressure, cholesterol, and other factors, was particularly sensitive to PCB exposure, reinforcing the notion that POPs contribute significantly to overall cardiovascular risk in this population [123,124].

The overall exposure effect analysis revealed a strong positive association between combined environmental exposures and the FRS [105,125]. FRS is a composite clinical metric that estimates an individual’s 10-year risk of developing CVD based on key factors such as blood pressure, cholesterol levels, age, and smoking status [101]. The observed relationship suggests that cumulative exposure to metals, PCBs, dioxins, and furans may exacerbate traditional cardiovascular risk factors, potentially accelerating the progression toward clinically significant CVD [123,126]. This finding underscores the public health relevance of addressing environmental mixtures as contributors to cardiovascular risk [127,128].

In contrast, for total cholesterol and triglycerides, the combined exposure effect demonstrated an unexpected pattern: greater perturbations were observed at lower exposure levels compared to higher levels, suggesting a possible non-monotonic or hormetic response [129,130]. The paradoxical increase in HDL and decreases in LDL, TC, and triglycerides with higher exposure levels might reflect compensatory biological responses, saturation of metabolic pathways, or compensatory homeostatic mechanisms at higher exposure levels, which dampen the lipid-disrupting effects of these contaminants [131]. Such patterns highlight the complexity of mixture toxicology, where low-level exposures may elicit disproportionately strong biological responses [132]. High pollutant burdens might trigger adaptive mechanisms enhancing HDL-mediated reverse cholesterol transport, thereby temporarily reducing LDL and triglyceride levels [133]. Alternatively, these findings might indicate hepatic disruption, altering lipid metabolism and resulting in atypical lipid profiles [134].

The remaining CVD-related markers showed either flat exposure–response relationships or wide credible intervals, indicating a lack of clear directional effects or considerable statistical uncertainty. These results may reflect insufficient power for some endpoints, biological variability, or weaker direct associations with the specific mixture components evaluated.

The single-variable effect analysis (Figure 6) revealed largely modest associations across the cardiovascular and lipid markers. However, 2,3,7,8-Tetrachlorodibenzofuran (TCDF) demonstrated a strong effect on total cholesterol, while 1,2,3,7,8-Pentachlorodibenzofuran (PeCDF) exhibited a notable effect on triglycerides, suggesting these congeners may disproportionately influence lipid metabolism when considered individually.

In the single-variable interaction analysis (Figure 7), where the effect of each exposure was evaluated at high versus low background levels of all other pollutants in the mixture apart from the exposure of interest, 1,2,3,7,8-Pentachlorodibenzofuran (PeCDF) showed the most pronounced interaction for triglycerides. This indicates that its lipid-disrupting effects are magnified in the presence of elevated background levels of the remaining contaminants, suggesting potential synergistic or additive mixture effects on triglyceride regulation [91,135].

Additional interactions with triglycerides were observed for 1,2,3,6,7,8-Hexachlorodibenzo-p-dioxin (HxCDD) and lead, although the credible intervals were wider, reflecting greater uncertainty. Noteworthy interaction patterns for LDL cholesterol were also observed: 2,3,7,8-Tetrachlorodibenzofuran (TCDF) and 1,2,3,7,8-Pentachlorodibenzo-p-dioxin (PeCDD) each demonstrated interaction effects with all other pollutants, and PCB 169 showed a similar pattern, suggesting these chemicals may modulate LDL levels in ways influenced by the surrounding chemical mixture.

Elevations in TC, HDL, and LDL cholesterol observed in our study suggest disruptions in lipid metabolism, potentially influenced by the pollutants studied [136]. Chemical mixtures, particularly PCBs, dioxins, and furans, are known endocrine disruptors capable of altering lipid homeostasis via modulation of hepatic enzymes involved in cholesterol synthesis and metabolism [134,137]. For instance, PCBs and furans might induce hepatic cytochrome P450 enzymes, modifying LDL receptor activity and lipoprotein metabolism, leading to altered cholesterol profiles [138,139]. Our results, indicating both elevations and inverse relationships in lipid markers, reflect complex, possibly non-monotonic interactions characteristic of these pollutants [129,130].

Finally, PCB 157 and PCB 156 displayed consistent interaction effects with all other pollutants for the Framingham risk score (FRS), reinforcing the hypothesis that specific PCB congeners may act as central modifiers of overall cardiovascular risk within complex environmental mixtures. These findings highlight the importance of assessing individual pollutant effects within the broader context of co-exposure to all other components of the mixture, which may substantially alter cardiovascular and metabolic outcomes.

In this study, a substantial proportion of the sample was classified as overweight or obese, which is a well-established independent risk factor for cardiovascular disease. Adiposity may also modulate the toxicokinetics of lipophilic compounds such as PCBs, dioxins, and furans, which accumulate in fat tissue and may have altered distribution or mobilization in individuals with higher body mass [91,140]. Furthermore, metabolic dysregulation associated with excess adiposity could enhance susceptibility to cardiotoxic effects of environmental exposures through inflammatory or endocrine pathways. Although BMI was included as a covariate in our models, the potential for effect modification warrants further investigation, particularly in the context of POP mixtures.

### 4.3. Comparison with the Previous Literature

The study’s results align with and extend growing evidence from epidemiological and toxicological research on environmental pollutants and cardiovascular disease. The observation that some metals correlate with CVD dysfunction is consistent with decades of research linking metal exposure to adverse cardiovascular outcomes [141]. For instance, prior population studies have documented positive associations between metals levels and both systolic and diastolic blood pressure, even at relatively low blood lead concentrations [141,142]. Our study revealed a complex relationship, with an inverse U-shaped pattern, providing deeper insight into how mercury may influence the cardiovascular system within a mixture context. A comprehensive review concluded that evidence is sufficient to infer a causal relationship between lead exposure and hypertension [143]. In our study, lead in a mixture did not show these same prominent effects. Mercury and cadmium exposure have similarly been implicated in vascular dysfunction, and experimental data show that these metals induce oxidative stress and endothelial damage, while cohort studies have found cadmium exposure associated with increased risks of coronary heart disease and stroke [144,145,146]. Our studies’ PIP findings, which identified mercury as a top contributor to elevated blood pressure and reduced HDL, corroborate these mechanisms by demonstrating metals’ deleterious impact on cardiovascular risk factors in a human population [145,146].

The relationship involving PCBs and cardiovascular outcomes (especially for PIP analysis involving SBP, FRS, and TC) also mirrors previously reported links. For example, background exposure to PCBs has been associated with a higher prevalence of hypertension. Notably, studies have found that individuals with elevated levels of dioxin-like PCBs were more likely to have high blood pressure, suggesting that these particular congeners (which can activate similar pathways as dioxins) are especially cardiotoxic. Everett et al. (2008) [124] and Pavuk et al. (2019) [147] first reported that certain dioxin-like PCB congeners were significantly correlated with hypertension in U.S. adults, and Uemura et al. (2008) [148] observed a direct relationship between higher dioxin-like PCB burdens and increased blood pressure in a Japanese cohort. The present study’s identification of PCB156 and PCB126 (both dioxin-like) as key factors for SBP and risk score supports these earlier findings, underscoring a hypertensive role of persistent organochlorines. Moreover, the link between PCB exposure and blood lipid disturbances has been noted in prior research on populations with high PCB exposure through diet [149]. For example, a study among Canadian Inuit found that higher blood PCB concentrations were associated with significantly elevated TC, LDL cholesterol, and triglycerides [150]. The lack of effect on HDL in that study [150] aligns with our results, which did not find PCBs improving HDL but did find them contributing to higher atherogenic lipid levels. These parallels with the existing literature lend credibility to the study’s results and suggest that the associations observed are not isolated findings, but part of a consistent pattern observed in diverse populations. In the case of dioxins and furans, the study’s findings resonate with epidemiological observations from extreme exposure events. Communities exposed to high levels of dioxins (such as the Seveso, Italy industrial accident in 1976 [151] have exhibited elevated rates of ischemic heart disease and cardiovascular mortality decades later. Likewise, veterans with substantial TCDD (dioxin) exposure have shown increased incidence of hypertension and metabolic syndrome in long-term follow-ups. Thus, the strong influence of dioxin-like compounds and furans on cardiovascular risk factors in this study coincides with the known cardiovascular toxicity of these pollutants from prior incidents and occupational studies.

### 4.4. Broader Health Implications

This study is unique because it looks at multiple pollutants on CVD outcomes when prior studies have focused on single exposures. These findings carry important public health implications, as they highlight how chronic low-level exposure to combined environmental toxicants may contribute to cardiovascular dysfunction in the general population. CVD arises from a complex interplay of genetic, behavioral, and environmental factors; our findings add to the growing evidence that environmental pollutant burdens represent a significant and underrecognized contributor to CVD risk. Even modest increases in blood pressure or unfavorable changes in cholesterol due to pollutants can translate into a meaningful rise in population-level CVD risk. For instance, a slight upward shift in SBP or a reduction in HDL across many exposed individuals could lead to more heart attacks and strokes over time. The results suggest that mixtures of POPs and heavy metals, which are ubiquitously present in food chains, air, water, and consumer products, may act in concert to subtly accelerate atherosclerotic processes and vascular injury. Metals like lead, cadmium, and mercury are known to induce oxidative stress and inflammation in blood vessels, while PCBs and dioxins can disrupt endocrine and metabolic pathways, promoting dyslipidemia, hypertension, and insulin resistance [152]. The co-occurrence of these exposures could therefore amplify harmful effects, as indicated by this study’s mixture analysis [130]. For example, metabolic byproducts or metabolites may further potentiate cardiovascular damage by generating reactive intermediates capable of binding and damaging proteins, lipids, and DNA. Metabolites could also amplify oxidative stress or disrupt normal metabolic signaling pathways critical for cardiovascular function, highlighting their role in mediating toxic effects [153].

In a broader context, the findings underscore the need to consider environmental exposures as part of cardiovascular risk assessment. Clinicians and public health officials might need to advocate for the reduction of exposure to these chemicals, be it through policies to lower lead and mercury exposure sources, cleanup of PCB-contaminated sites, or stricter regulation of industrial byproducts like dioxins [91,154]. Additionally, individuals in highly exposed communities (such as those relying on contaminated fish or living near industrial areas) may benefit from targeted interventions (dietary changes, chelation therapies, etc.) to mitigate pollutant-related risk. That said, regulating a mixture remains inherently challenging, which explains why most studies and policies have traditionally focused on single exposures. However, targeted interventions, such as reducing traffic in highly polluted areas, can lower the resuspension of contaminated soil into the air, while stricter controls on industrial emissions and wastewater discharges can reduce the spread of pollutants into both air and water systems, thereby diminishing overall human exposure.

### 4.5. Strengths and Limitations

#### 4.5.1. Strengths

This analysis has several notable strengths. First, it examines a broad range of environmental exposures (metals, PCBs, dioxins, furans) simultaneously, using data from a well-characterized population. The use of multiple statistical approaches on traditional linear regression and advanced BKMR thus provides a comprehensive assessment of both individual exposure effects and mixture effects. By comparing these methods, we gained insights into how co-exposures might confound or modify associations. The study controlled for many potential confounders (age, gender, BMI, ethnicity, income, smoking, alcohol), which strengthens the validity of the observed associations by reducing spurious influence from those factors. Another strength is the inclusion of PIP analysis, which leverages Bayesian modeling to identify the pollutants (or pollutant groups) most strongly linked to outcomes. This helped distill the complex mixture into a ranked list of culprit chemicals, which is valuable for risk prioritization [105,155]. Additionally, the measurement of exposures and outcomes was conducted with standardized protocols (e.g., blood pressure measured by trained personnel, chemical analyses by CDC laboratories), lending credibility to data quality [78]. Lastly, this work addresses an important gap by relating environmental mixture exposures to subclinical cardiovascular indicators, an area of growing public health interest as even low-level environmental toxicants are increasingly recognized as cardiovascular risk factors [123,133,142]

#### 4.5.2. Limitations

Despite its contributions, the study has some limitations. Foremost, the NHANES design is cross-sectional, measuring both exposures and outcomes simultaneously. This precludes any firm conclusions about causality or the temporal sequence of events. We cannot be sure whether the exposures contributed to the changes in cardiovascular markers or if individuals with certain characteristics (e.g., poorer health or diet) accumulated different levels of pollutants. Longitudinal studies would be needed to confirm causation. Another limitation is the potential for residual confounding and measurement error. While many confounders were adjusted for, there may be other factors we did not account for, such as dietary patterns (beyond alcohol) or physical activity, which could influence both exposure and outcome (for instance, fish consumption affects mercury levels and also cardiovascular health). Additionally, some exposure measurements may be imprecise; persistent organochlorines in blood reflect long-term accumulation, but single measurements of metals may not accurately capture long-term exposure. Finally, the health outcomes are intermediate risk markers, not actual disease endpoints. While increases in BP or alterations in lipids are established risk factors for CVD, the clinical significance of the relatively modest changes observed here is uncertain. For example, a few mmHg rise in SBP associated with exposure might slightly raise the population risk of hypertension, but we did not directly assess outcomes like heart attacks or strokes. Nevertheless, many people worldwide still carry these persistent chemicals [149], and certain communities (like those consuming high fish or near contaminated sites) have exposures comparable to or higher than our study sample.

Additionally, this study did not apply TEF-based grouping of dioxin-like compounds, as our objective was to evaluate congener-specific effects within a broader multi-class chemical mixture. While toxic equivalency methods offer value in focused risk assessments, their use would have required narrowing the chemical set and pre-aggregating compounds, limiting the flexibility of our exploratory modeling approach. Future studies focused specifically on dioxin-like toxicity may benefit from incorporating TEQ-based methods to assess cumulative potency.

Finally, this study was grounded in a strong a priori hypothesis that combined exposure to 32 environmental chemicals that influence cardiovascular disease risk. While BKMR enables the exploration of complex, non-linear, and interactive relationships within mixtures, the individual exposure–response and interaction patterns identified should be interpreted as hypothesis-generating rather than confirmatory. Such findings are valuable for guiding future mechanistic and experimental studies that can more precisely test and validate these observed associations under controlled conditions.

Despite these limitations, the study’s findings are biologically plausible and align with a growing body of literature linking environmental toxicants to cardiovascular risk factors. By acknowledging the above limitations, we temper our conclusions and highlight areas for future research, such as prospective studies and mechanistic investigations, to better establish causality and interaction effects.

## 5. Conclusions

In conclusion, our study contributes to the growing recognition that environmental pollutant exposures are linked to cardiovascular risk factors in meaningful ways. The positive associations between the pollutant mixture and SBP and risk score, as well as the identification of specific toxicants influencing cholesterol and blood pressure, support a clear relationship between environmental contaminants and cardiovascular disease risk. While causal inference is limited by the cross-sectional design, the findings are consistent with biological mechanisms (e.g., metal-induced oxidative stress, AhR-mediated dyslipidemia) and epidemiological evidence from other cohorts. Future research should build on these observations by employing longitudinal designs and exploring molecular pathways (e.g., inflammation, endothelial dysfunction) through which these exposures exert their effects. Public health practitioners should be cognizant of environmental exposures when assessing cardiovascular risk in populations, particularly in communities with known high exposure levels. Efforts to minimize exposure to POPs and heavy metals could be considered part of cardiovascular disease prevention strategies. Ultimately, reducing environmental pollutant burdens may contribute to improved cardiovascular health at the population level.

## Figures and Tables

**Table 1:** Descriptive statistics of the unadjusted covariates by gender with mean (s.d) for and (*n*, %).

*n* = 432		Overall Mean (S.D)	Male Mean (S.D)	Female Mean (S.D)
**AGE (years)**	Age	46.26 (17.17)	47.56 (17.54)	45.16 (16.82)
**BMI**	BMI	28.31 (6.07)	27.98 (5.26)	28.60 (6.68)
**INCOME**	$0 to $14,999	13.19% (57)	12.6% (25)	13.6% (32)
	$15,000 to $24,999	14.81% (64)	13.2% (26)	16.2% (38)
	$25,000 to $44,999	24.31% (105)	25.2% (50)	23.5% (55)
	$45,000 to $64,999	9.49% (41)	14.1% (28)	14.1% (33)
	$65,000 to $74,999	6.25% (27)	7.6% (15)	5.1% (12)
	$75,000 and over	22.22% (96)	22.7% (45)	21.8% (51)
**(%, *n*)**	Unavailable	5.09% (22)	4.5% (9)	5.6% (13)
**ETHNICITY** **(%, *n*)**	Mexican American	57.17% (247)	57.1% (113)	57.3% (134)
	Other Hispanic	16.9% (73)	17.2% (34)	16.7% (39)
	Non-Hispanic White	19.21% (83)	17.2% (34)	20.9% (49)
	Non-Hispanic Black	3.24% (14)	3.5% (7)	3.0% (7)
	Other Race	3.47% (15)	5.1% (10)	2.1% (5)
**SMOKERS (%, *n*)**	No	29.40% (127)	33.8% (67)	25.6% (60)
	Yes	18.98% (82)	25.8% (51)	13.2% (31)
	NA	51.62% (223)	40.4% (80)	61.1% (143)
**ALCOHOL**	No	25.7% (111)	17.7% (35)	32.5% (76)
	Yes	68.5% (296)	80.3% (159)	58.5% (137)
**(%, *n*)**	NA	5.8% (25)	2.0% (4)	9.0% (21)

**Table 2:** Distribution of exposure and study variables.

	Overall Mean (S.D)	Male Mean (S.D)	Female Mean (S.D)	*p*_Value
Cadmium	0.58 (0.76)	0.60 (0.81)	0.56 (0.72)	0.6613
Lead	2.07 (2.51)	2.68 (3.40)	1.55 (1.13)	**<0.0001**
Mercury	1.86 (2.91)	1.84 (2.66)	1.87 (3.12)	0.927
PCB28	5.41 (2.79)	5.30 (2.66)	5.50 (2.90)	0.4558
PCB66	1.64 (1.33)	1.45 (0.85)	1.80 (1.61)	**0.0034**
PCB74	7.97 (10.34)	6.88 (9.56)	8.90 (10.90)	**0.0408**
PCB105	2.18 (3.86)	1.84 (3.57)	2.47 (4.08)	0.0854
PCB118	10.92 (18.13)	9.33 (16.43)	12.27 (19.37)	0.0882
PCB156	5.69 (6.34)	6.37 (6.58)	5.11 (6.08)	**0.0408**
PCB157	1.36 (1.57)	1.49 (1.63)	1.25 (1.52)	0.1113
PCB167	1.38 (1.98)	1.36 (2.05)	1.40 (1.92)	0.8553
PCB189	0.39 (0.66)	0.46 (0.73)	0.33 (0.58)	**0.048**
PCB126	26.11 (32.02)	23.13 (28.01)	28.64 (34.91)	0.0694
PCB81	6.04 (4.73)	6.28 (5.82)	5.83 (3.57)	0.3365
PCB169	15.94 (14.81)	18.96 (16.97)	13.39 (12.16)	**0.0001**
1,2,3,7,8-PeCDD	4.49 (4.23)	4.72 (4.10)	4.30 (4.33)	0.3012
1,2,3,4,7,8-HxCDD	4.20 (3.45)	4.37 (3.61)	4.05 (3.31)	0.3279
1,2,3,6,7,8-HxCDD	28.35 (24.48)	30.21 (25.76)	26.77 (23.28)	0.1494
1,2,3,7,8,9-HxCDD	4.44 (3.68)	4.37 (3.30)	4.50 (3.97)	0.7103
1,2,3,4,6,7,8-HpCDD	36.81 (35.32)	32.19 (26.33)	40.72 (41.08)	**0.0095**
1,2,3,4,6,7,8,9-OCDD	297.51 (309.91)	250.82 (274.35)	337.01 (332.58)	**0.0033**
2,3,7,8-TCDD	1.99 (2.24)	1.89 (2.36)	2.07 (2.13)	0.3863
2,3,7,8-TCDF	1.61 (0.81)	1.69 (0.88)	1.53 (0.73)	**0.0417**
1,2,3,7,8-PeCDF	1.67 (0.66)	1.78 (0.76)	1.58 (0.55)	0.0019
2,3,4,7,8-PeCDF	5.20 (3.92)	5.40 (4.03)	5.03 (3.81)	**0.3298**
1,2,3,4,7,8-HxCDF	4.19 (2.90)	4.41 (2.92)	4.00 (2.87)	0.1421
1,2,3,6,7,8-HxCDF	3.74 (2.61)	3.96 (2.76)	3.56 (2.46)	0.1181
1,2,3,7,8,9-HxCDF	1.91 (0.74)	2.02 (0.71)	1.82 (0.75)	**0.0049**
2,3,4,6,7,8-HxCDF	1.90 (0.74)	2.02 (0.71)	1.80 (0.74)	**0.0019**
1,2,3,4,6,7,8-HpCDF	9.48 (16.74)	10.96 (23.27)	8.23 (7.57)	0.1139
1,2,3,4,7,8,9-HpCDF	2.03 (0.84)	2.23 (0.92)	1.87 (0.73)	**<0.0001**
1,2,3,4,6,7,8,9-OCDF	3.60 (2.99)	4.06 (3.35)	3.20 (2.58)	**0.0033**

*p*-values were computed using a *t*-test. Descriptive values are unadjusted means (SD). Bold font represents *p*-values indicating statistical significance.

**Table 3:** Linear regression analysis for CVD markers. Coefficient and 95% CI presented. Adjusted for gender, age, ethnicity, income, smoking, alcohol, and BMI.

	FRS			DBP				SBP		HDL Cholesterol		
Variable	Estimate	2.50%	97.50%	Estimate	2.50%	97.50%	Estimate	2.50%	97.50%	Estimate	2.50%	97.50%
Cadmium	0.0524	−0.2425	0.3473	0.2543	−2.0124	2.5211	0.3376	−2.6814	3.3567	0.8949	−1.6259	3.4157
Lead	0.0326	−0.0847	0.1498	−0.3343	−1.2356	0.567	−0.4529	−1.6534	0.7476	**1.6352**	**0.6328**	**2.6375**
**Mercury**	**−0.1294**	**−0.2366**	**−0.0221**	−0.3496	−1.1742	0.4749	−0.7925	−1.8907	0.3056	0.5281	−0.3889	1.445
PCB28	0.0781	−0.0789	0.2351	−0.3706	−1.5774	0.8361	0.1664	−1.4409	1.7737	0.078	−1.264	1.42
PCB66	0 × 10⁰	−0.5203	0.5204	−1.787	−5.7868	2.2131	−0.3795	−5.7069	4.948	−1.517	−5.9652	2.9311
PCB74	−0.0362	−0.1646	0.0922	−0.242	−1.2288	0.7449	−0.0446	−1.359	1.2699	−0.3141	−1.4115	0.7834
PCB105	0.4967	−0.2493	1.2427	2.955	−2.7797	8.6892	−2.4813	−10.1189	5.1564	**7.2819**	**0.9049**	**13.6589**
PCB118	−0.1188	−0.296	0.0584	−0.6759	−2.0379	0.6861	−0.0098	−1.8238	1.8043	**−1.5648**	**−3.0794**	**−0.0502**
PCB156	0.0589	−0.2569	0.3747	1.441	−0.9861	3.8687	−0.244	−3.4769	2.9891	−2.5911	−5.2905	0.1083
PCB157	0.0641	−1.3694	1.4977	−4.796	−15.8161	6.2233	−0.6909	−15.3679	13.9862	11.845	−0.4095	24.0995
PCB167	0.4992	−0.3142	1.3126	3.43	−2.8221	9.6824	**8.4078**	**0.0805**	**16.7351**	2.3399	−4.6129	9.2928
PCB189	−0.2927	−0.8616	0.2762	0.1178	−4.2552	4.4908	−0.0504	−5.8747	5.7739	−3.419	−8.2819	1.444
PCB126	−0.0216	−0.0463	0.003	−0.0461	−0.2358	0.1436	0.1438	−0.1089	0.3964	−0.0226	−0.2335	0.1884
PCB81	0.0363	−0.0515	0.1241	0.5052	−0.17	1.1804	−0.6219	−1.5212	0.2774	−0.2184	−0.9693	0.5325
PCB169	−0.0186	−0.0729	0.0356	−0.0857	−0.5025	0.3311	−0.2218	−0.7769	0.3333	−0.2154	−0.6789	0.2481
1,2,3,7,8-PeCDD	0.0232	−0.1216	0.168	−0.1989	−1.312	0.9142	1.2638	−0.2187	2.7463	1.1602	−0.0776	2.3981
1,2,3,4,7,8-HxCDD	−0.0947	−0.2586	0.0693	−0.2817	−1.5423	0.9789	−0.063	−1.742	1.616	**1.8077**	**0.4058**	**3.2096**
1,2,3,6,7,8-HxCDD	−0.0076	−0.0343	0.0192	−0.0011	−0.2065	0.2043	0.1795	−0.0941	0.4531	−0.2212	−0.4496	0.0072
1,2,3,7,8,9-HxCDD	−0.0133	−0.1441	0.1174	−0.2434	−1.2486	0.7618	−1.0308	−2.3696	0.308	−0.5812	−1.6991	0.5366
1,2,3,4,6,7,8-HpCDD	0.0047	−0.016	0.0254	−0.0683	−0.2274	0.0909	−0.1302	−0.3421	0.0818	**−0.1826**	**−0.3595**	**−0.0056**
1,2,3,4,6,7,8,9-OCDD	−0.0009	−0.0031	0.0014	**0.0182**	**0.0008**	**0.0356**	**0.0359**	**0.0128**	**0.0591**	0.0126	−0.0068	0.0319
2,3,7,8-TCDD	0.1505	−0.1338	0.4348	0.7044	−1.4809	2.8898	0.2198	−2.6909	3.1304	0.1668	−2.2635	2.597
2,3,7,8-TCDF	−0.2456	−1.1107	0.6194	2.056	−4.5931	8.7058	−1.1339	−9.9902	7.7224	**9.3056**	**1.911**	**16.7002**
1,2,3,7,8-PeCDF	0.5568	−0.6099	1.7235	−3.498	−12.4659	5.4708	0.0152	−11.9296	11.96	**−12.5138**	**−22.4871**	**−2.5405**
2,3,4,7,8-PeCDF	0.0213	−0.1563	0.1989	−0.0248	−1.3901	1.3405	**−2.1838**	**−4.0022**	**−0.3654**	0.2497	−1.2686	1.768
1,2,3,4,7,8-HxCDF	0.0002	−0.2087	0.2091	−0.7256	−2.3316	0.8803	0.4267	−1.7122	2.5657	−0.9021	−2.688	0.8838
1,2,3,6,7,8-HxCDF	0.0025	−0.1992	0.2042	0 × 10⁰	−1.5503	1.5504	−0.9673	−3.0322	1.0976	0.5732	−1.1508	2.2973
1,2,3,7,8,9-HxCDF	−0.1117	−0.891	0.6675	−2.92	−8.9105	3.0702	−7.9312	−15.9097	0.0472	0.1835	−6.4781	6.8451
2,3,4,6,7,8-HxCDF	0.2336	−0.6677	1.1349	6.342	−0.5864	13.2707	7.4923	−1.7358	16.7204	2.4686	−5.2364	10.1735
1,2,3,4,6,7,8-HpCDF	0.0283	−0.0085	0.0651	0.0772	−0.2056	0.3599	−0.1359	−0.5124	0.2407	0.2335	−0.0809	0.548
1,2,3,4,7,8,9-HpCDF	−0.0298	−0.3969	0.3374	0.9836	−1.8387	3.8059	**4.6609**	**0.902**	**8.4199**	0.2411	−2.8974	3.3796
1,2,3,4,6,7,8,9-OCDF	−0.0112	−0.1118	0.0894	−0.618	−1.3916	0.1557	0.4179	−0.6126	1.4483	−0.759	−1.6193	0.1014
	LDL Cholesterol				Total Cholesterol				Triglycerides			
Variable	Estimate		2.50%	97.50%	Estimate		2.50%	97.50%	Estimate		2.50%	97.50%
Cadmium	−6.0506		−12.1415	0.0402	−3.7856		−9.9776	2.4065	6.8229		−4.225	17.8708
Lead	1.2753		−1.1466	3.6972	2.3144		−0.1477	3.6972	−3.01		−7.4029	1.383
**Mercury**	0.1087		−2.1068	2.3242	0.1087		−1.8907	2.3242	−0.3652		−4.225	3.9022
PCB28	−0.9122		−4.1548	2.3305	0.1664		−1.4409	1.7737	−2.2123		−2.9085	2.7421
PCB66	1.6917		−9.0562	12.4397	−0.5855		−5.7069	10.341	−3.8406		−11.512	10.341
PCB74	0.3296		−2.3222	2.9814	0.4055		−2.3222	3.1013	0.4055		−2.2904	6.8105
PCB105	10.7004		−4.7082	26.109	**16.702**		**1.0374**	**32.3666**	−4.641		−33.72	22.178
PCB118	−3.5709		−7.2307	0.0888	**−4.641**		**−8.3615**	**−0.9205**	2.3313		−4.3069	8.9695
PCB156	5.4245		−1.098	11.9469	5.4245		−3.7879	9.4737	0.0808		−11.7499	11.9116
PCB157	−16.7059		−46.3162	12.9044	−16.7059		−46.3162	13.9862	−29.8984		−15.3679	13.9862
PCB167	15.0296		−1.7705	31.8296	13.2651		−1.7705	31.8296	−20.0693		−51.7845	11.2449
PCB189	**−13.2181**		**−24.9684**	**−1.4678**	**−12.376**		**−24.3219**	**−1.4678**	21.1604		−5.1529	30.3442
PCB126	−0.3785		−0.8882	0.1312	−0.1211		−0.8882	0.1312	**1.3933**		**0.4679**	**4.3539**
PCB81	0.7397		−1.0746	2.554	0.7397		−1.0746	2.554	−2.8892		−4.5673	2.4178
PCB169	**−1.7456**		**−2.8655**	**−0.6256**	**−1.522**		**−2.6606**	**−0.3835**	**2.1635**		**0.1321**	**4.1949**
1,2,3,7,8-PeCDD	−0.4382		−3.4291	2.5528	1.1698		−1.8708	4.2104	1.1698		−3.1078	7.7425
1,2,3,4,7,8-HxCDD	0.5033		−2.8841	3.8906	1.9035		−1.5401	3.8906	−2.0164		−8.1605	4.9059
1,2,3,6,7,8-HxCDD	−0.5101		−1.062	0.0419	−0.5101		−1.062	0.0419	−1.0918		−2.092	0.4731
1,2,3,7,8,9-HxCDD	1.6823		−1.0187	4.3833	−1.0308		−1.2517	0.308	1.8724		−1.8708	7.7425
1,2,3,4,6,7,8-HpCDD	0.2382		−0.1894	0.6658	0.033		−0.1894	0.6658	−0.1302		−0.4017	0.6734
1,2,3,4,6,7,8,9-OCDD	−0.0371		−0.0838	0.0096	−0.0188		−0.0663	0.0287	0.029		−0.0557	0.1137
2,3,7,8-TCDD	0.309		−5.5632	6.1812	0.3048		−5.6649	6.2745	−0.9104		−11.5616	9.7409
2,3,7,8-TCDF	−0.5556		−18.423	17.3117	4.2134		−13.9507	22.3776	−23.0568		−55.4654	9.3519
1,2,3,7,8-PeCDF	−0.4972		−24.5954	23.6011	−12.5138		−34.5954	23.6011	0.0152		−34.2253	60.3987
2,3,4,7,8-PeCDF	2.6819		−0.9866	6.3505	3.1156		−0.9866	6.8451	3.1156		−0.6139	6.8452
1,2,3,4,7,8-HxCDF	2.7534		−1.5619	7.0686	2.4715		−1.9159	7.0686	2.9631		−1.9154	6.8584
1,2,3,6,7,8-HxCDF	−0.0623		−4.2281	4.1036	0.6035		−4.2281	4.1036	0.5045		−3.6316	4.8386
1,2,3,7,8,9-HxCDF	−4.5552		−20.6514	11.541	−4.5552		−20.6514	11.541	−7.9312		−63.7826	−5.3902
2,3,4,6,7,8-HxCDF	−2.6908		−21.3081	15.9265	−2.6908		−17.7634	20.0898	1.1632		−26.8828	40.6553
1,2,3,4,6,7,8-HpCDF	−0.1103		−0.87	0.6495	−0.2424		−0.87	0.6495	**−1.8075**		**−3.1859**	**−0.4299**
1,2,3,4,7,8,9-HpCDF	**−11.0937**		**−18.6773**	**−3.5102**	**−12.3215**		**−20.0315**	**−3.5102**	−7.3934		−21.1488	6.9623
1,2,3,4,6,7,8,9-OCDF	0.5159		−1.563	2.5947	0.5159		−1.563	2.5947	2.7165		−0.6126	1.4483

Adjusted for gender, age, ethnicity, income, smoking, alcohol, and BMI. Bold font for *confidence intervals* indicates statistical significance.

**Table 4:** Hierarchical BKMR results for cardiovascular related outcomes DBP, SBP, FRS, HDL, LDL, TC, and triglycerides.

		DBP			SBP		FRS		
Variable	Group	Group PIP	Conditional PIP		Group PIP	Conditional PIP	Group PIP	Conditional PIP	
Cadmium	1	0.1662	0.0173		0.0054	0.2593	0.0006	0	
Lead	1	0.1662	0.0327		0.0054	0.3704	0.0006	0	
Mercury	1	0.1662	0.95		0.0054	0.3704	0.0006	1	
PCB28	2	0.0703	0.0381		0.9019	0.0008	0.9336	0.0018	
PCB66	2	0.0703	0.0188		0.9019	0.0412	0.9336	0	
PCB74	2	0.0703	0.1507		0.9019	0.0267	0.9336	0.0006	
PCB105	2	0.0703	0.0148		0.9019	0.0169	0.9336	0.0001	
PCB118	2	0.0703	0.0205		0.9019	0.0608	0.9336	0.0007	
PCB156	2	0.0703	0.0939		0.9019	0	0.9336	0.6638	
PCB157	2	0.0703	0.0825		0.9019	0	0.9336	0.1381	
PCB167	2	0.0703	0.0324		0.9019	0.0024	0.9336	0.0001	
PCB189	2	0.0703	0.0324		0.9019	0.0003	0.9336	0	
PCB126	2	0.0703	0.0267		0.9019	0.8508	0.9336	0	
PCB81	2	0.0703	0.06		0.9019	0	0.9336	0	
PCB169	2	0.0703	0.4295		0.9019	0	0.9336	0.2073	
1,2,3,7,8-PeCDD	3	0.0978	0.2834		0.2299	0.0642	0.066	0.2121	
1,2,3,4,7,8-HxCDD	3	0.0978	0.2036		0.2299	0.003	0.066	0.3861	
1,2,3,6,7,8-HxCDD	3	0.0978	0.2931		0.2299	0.0046	0.066	0.5927	
1,2,3,7,8,9-HxCDD	3	0.0978	0.0961		0.2299	0.0863	0.066	0	
1,2,3,4,6,7,8-HpCDD	3	0.0978	0.0446		0.2299	0.3689	0.066	0	
1,2,3,4,6,7,8,9-OCDD	3	0.0978	0.0209		0.2299	0.0268	0.066	0	
2,3,7,8-TCDD	3	0.0978	0.0585		0.2299	0.0002	0.066	0	
2,3,7,8-TCDF	4	0.1164	0.0144		0.183	0.0136	0.0239	0.0151	
1,2,3,7,8-PeCDF	4	0.1164	0.0491		0.183	0.0052	0.0239	0.0084	
2,3,4,7,8-PeCDF	4	0.1164	0.1391		0.183	0.92	0.0239	0	
1,2,3,4,7,8-HxCDF	4	0.1164	0.0325		0.183	0.0466	0.0239	0.2993	
1,2,3,6,7,8-HxCDF	4	0.1164	0.0306		0.183	0.0037	0.0239	0.51	
1,2,3,7,8,9-HxCDF	4	0.1164	0.1404		0.183	0.0015	0.0239	0.0124	
2,3,4,6,7,8-HxCDF	4	0.1164	0.1431		0.183	0.0033	0.0239	0.0435	
1,2,3,4,6,7,8-HpCDF	4	0.1164	0		0.183	0	0.0239	0	
1,2,3,4,7,8,9-HpCDF	4	0.1164	0.0908		0.183	0.061	0.0239	0	
1,2,3,4,6,7,8,9-OCDF	4	0.1164	0.0745		0.183	0	0.0239	0	
		HDL		LDL Cholesterol		Total Cholesterol		Triglycerides	
Variable	Group	Group PIP	Conditional PIP	Group PIP	Conditional PIP	Group PIP	Conditional PIP	Group PIP	Conditional PIP
Cadmium	1	0.0882	0	0.2946	0.9293	0.0052	0.9845	0.8211	0.0002
Lead	1	0.0882	0.0163	0.2946	0.0648	0.0052	0.0155	0.8211	0.9208
Mercury	1	0.0882	0.9837	0.2946	0.0058	0.0052	0	0.8211	0.079
PCB28	2	0.0491	0.1079	0.9383	0	0.0395	0	0.4136	0.0089
PCB66	2	0.0491	0.0179	0.9383	0.001	0.0395	0	0.4136	0.0037
PCB74	2	0.0491	0.0204	0.9383	0.0017	0.0395	0.1266	0.4136	0.0147
PCB105	2	0.0491	0.048	0.9383	0.0014	0.0395	0	0.4136	0.0112
PCB118	2	0.0491	0.0798	0.9383	0.0037	0.0395	0.0162	0.4136	0.0074
PCB156	2	0.0491	0.0953	0.9383	0.004	0.0395	0.2452	0.4136	0.0181
PCB157	2	0.0491	0.1743	0.9383	0.0084	0.0395	0.2817	0.4136	0.027
PCB167	2	0.0491	0.0497	0.9383	0.006	0.0395	0.0794	0.4136	0.0155
PCB189	2	0.0491	0.0204	0.9383	0.0028	0.0395	0.0993	0.4136	0.017
PCB126	2	0.0491	0.0497	0.9383	0.002	0.0395	0	0.4136	0.8508
PCB81	2	0.0491	0	0.9383	0	0.0395	0	0.4136	0.0147
PCB169	2	0.0491	0.1531	0.9383	0.9698	0.0395	0.157	0.4136	0.0109
1,2,3,7,8-PeCDD	3	0.0733	0.4037	0.3343	0.5271	0.0112	0.0573	0.3652	0.0194
1,2,3,4,7,8-HxCDD	3	0.0733	0.0327	0.3343	0.0125	0.0112	0.0072	0.3652	0.0194
1,2,3,6,7,8-HxCDD	3	0.0733	0.2714	0.3343	0.1351	0.0112	0.8172	0.3652	0.5877
1,2,3,7,8,9-HxCDD	3	0.0733	0.1304	0.3343	0.1811	0.0112	0.0358	0.3652	0.1518
1,2,3,4,6,7,8-HpCDD	3	0.0733	0.1335	0.3343	0.0869	0.0112	0.0287	0.3652	0.0157
1,2,3,4,6,7,8,9-OCDD	3	0.0733	0.0245	0.3343	0.0141	0.0112	0.0287	0.3652	0.1952
2,3,7,8-TCDD	3	0.0733	0.0001	0.3343	0.0432	0.0112	0.0251	0.3652	0.0194
2,3,7,8-TCDF	4	0.0536	0	1	0.1706	1	1	1	0
1,2,3,7,8-PeCDF	4	0.0536	0.0105	1	0.8294	1	0	1	1
2,3,4,7,8-PeCDF	4	0.0536	0.4892	1	0	1	0	1	0
1,2,3,4,7,8-HxCDF	4	0.0536	0.0635	1	0	1	0	1	0
1,2,3,6,7,8-HxCDF	4	0.0536	0.0152	1	0	1	0	1	0
1,2,3,7,8,9-HxCDF	4	0.0536	0.0916	1	0	1	0	1	0
2,3,4,6,7,8-HxCDF	4	0.0536	0.0672	1	0	1	0	1	0
1,2,3,4,6,7,8-HpCDF	4	0.0536	0	1	0	1	0	1	0
1,2,3,4,7,8,9-HpCDF	4	0.0536	0.0497	1	0	1	0	1	0
1,2,3,4,6,7,8,9-OCDF	4	0.0536	0.0695	1	0	1	0	1	0

Adjusted for gender, age, ethnicity, income, smoking, alcohol, and BMI.

## Data Availability

The NHANES dataset is publicly available online, accessible at https://wwwn.cdc.gov/nchs/nhanes/continuousnhanes/overview.aspx?BeginYear=2003 (accessed on 1 May 2025).

## Supplementary Materials

The following supporting information can be downloaded at: https://www.mdpi.com/article/10.3390/jox15030094/s1, Figure S1: Dot plot of descriptive summary of exposures by gender; Figure S2: Spearman correlation heatmap for DBP and exposures (metals, PCBs, dioxins, and furans); Figure S3: Spearman correlation heatmap for SBP and exposures; Figure S4: Spearman correlation heatmap for HDL and exposures; Figure S5: Spearman correlation heatmap for LDL and exposures; Figure S6: Spearman correlation heatmap for Total Cholesterol and exposures; Figure S7: Spearman correlation heatmap for Triglycerides and exposures; Figure S8: Univariate exposure–response function of exposures on DBP; Figure S9: Univariate exposure–response function of exposures on SBP; Figure S10: Univariate exposure–response function of exposures on HDL; Figure S11: Univariate exposure–response function of exposures on LDL; Figure S12: Univariate exposure–response function of exposures on Total Cholesterol; Figure S13: Univariate exposure–response function of exposures on Triglycerides; Figure S14: Overall exposure effect of combined exposures on DBP examined at 0.25–0.75 quantiles as compared to the 0.5 quantile; Figure S15: Overall exposure effect of combined exposures on SBP examined at 0.25–0.75 quantiles as compared to the 0.5 quantile; Figure S16: Overall exposure effect of combined exposures on HDL examined at 0.25–0.75 quantiles as compared to the 0.5 quantile; Figure S17: Overall exposure effect of combined exposures on LDL examined at 0.25–0.75 quantiles as compared to the 0.5 quantile; Figure S18: Overall exposure effect of combined exposures on Total Cholesterol examined at 0.25–0.75 quantiles as compared to the 0.5 quantile; Figure S19: Overall exposure effect of combined exposures on Triglycerides examined at 0.25–0.75 quantiles as compared to the 0.5 quantile; Figure S20: Single-variable exposure effect on DBP across fixed quantiles of other exposures; Figure S21: Single-variable exposure effect on SBP across fixed quantiles of other exposures; Figure S22: Single-variable exposure effect on HDL across fixed quantiles of other exposures; Figure S23: Single-variable exposure effect on LDL across fixed quantiles of other exposures; Figure S24: Single-variable exposure effect on Total Cholesterol across fixed quantiles of other exposures; Figure S25: Single-variable exposure effect on Triglycerides across fixed quantiles of other exposures; Figure S26: Single-variable interaction effects from 25th to 75th percentile on DBP; Figure S27: Single-variable interaction effects from 25th to 75th percentile on SBP; Figure S28: Single-variable interaction effects from 25th to 75th percentile on HDL; Figure S29: Single-variable interaction effects from 25th to 75th percentile on LDL; Figure S30: Single-variable interaction effects from 25th to 75th percentile on Total Cholesterol; Figure S31: Single-variable interaction effects from 25th to 75th percentile on Triglycerides; Figure S32: Bivariate exposure–response functions of combined exposures on DBP at quantiles 0.25, 0.5, and 0.75; Figure S33: Bivariate exposure–response functions of combined exposures on SBP; Figure S34: Bivariate exposure–response functions of combined exposures on HDL; Figure S35: Bivariate exposure–response functions of combined exposures on LDL; Figure S36: Bivariate exposure–response functions of combined exposures on Total Cholesterol; Figure S37: Bivariate exposure–response functions of combined exposures on Triglycerides; Table S1: Descriptive statistics (mean, SD) of cardiovascular biomarkers by gender.

## Appendix A

**Table A1:** Descriptions of PCBs, dioxins, and furans.

Chemical Group	Chemical Code/Name	Study Key	Description
PCBs	PCB28 Lipid Adj (ng/g)	PCB28	Polychlorinated biphenyl congener 28; persistent organic pollutant, bioaccumulative.
	PCB66 Lipid Adj (ng/g)	PCB66	PCB congener 66; moderately chlorinated PCB, bioaccumulative, persistent pollutant.
	PCB74 Lipid Adj (ng/g)	PCB74	PCB congener 74; persistent, bioaccumulative, potentially toxic pollutant.
	PCB105 Lipid Adj (ng/g)	PCB105	PCB congener 105; dioxin-like, bioaccumulative, toxic pollutant.
	PCB118 Lipid Adj (ng/g)	PCB118	PCB congener 118; dioxin-like PCB, persistent, bioaccumulative, toxic.
	PCB156 Lipid Adj (ng/g)	PCB156	PCB congener 156; highly chlorinated, dioxin-like, persistent pollutant.
	PCB157 Lipid Adj (ng/g)	PCB157	PCB congener 157; persistent organic pollutant, moderate toxicity.
	PCB167 Lipid Adj (ng/g)	PCB167	PCB congener 167; environmentally persistent, potential toxicological concern.
	PCB189 Lipid Adj (ng/g)	PCB189	PCB congener 189; highly chlorinated, very persistent, known for bioaccumulation.
	PCB126-3,3′,4,4′,5-pncb Lipid Adj (pg/g)	PCB126	Highly toxic, dioxin-like PCB congener; potent bioaccumulative chemical.
	PCB81-3,4,4′,5-tcb Lipid Adj (pg/g)	PCB81	Dioxin-like PCB congener; highly toxic, persistent pollutant.
	PCB169-3,3′,4,4′,5,5′-hxcb Lipid Adj (pg/g)	PCB169	Highly chlorinated dioxin-like PCB, significant bioaccumulation, toxicity.
Dioxins	LBXD01LA– 1,2,3,7,8-Pentachlorodibenzo-p-dioxin Lipid Adj (pg/g)	Dioxin1- 1,2,3,7,8-PeCDD	Pentachlorodibenzo-p-dioxin; highly toxic, persistent environmental contaminant.
	LBXD02LA– 1,2,3,4,7,8-Hexachlorinated dioxin Lipid Adj (pg/g)	Dioxin2- 1,2,3,4,7,8-HxCDD	Hexachlorinated dioxin; highly toxic, persistent, carcinogenic contaminant.
	LBXD03LA– 1,2,3,6,7,8-Hexachlorinated dioxin Lipid Adj (pg/g)	Dioxin3- 1,2,3,6,7,8-HxCDD	Hexachlorinated dioxin; persistent, bioaccumulative, highly toxic.
	LBXD04LA– 1,2,3,7,8,9-Hexachlorinated dioxin Lipid Adj (pg/g)	Dioxin4- 1,2,3,7,8,9-HxCDD	Hexachlorinated dioxin; persistent organic pollutant, toxicological concern.
	LBXD05LA– 1,2,3,4,6,7,8-Heptachlorinated dioxin Lipid Adj (pg/g)	Dioxin5- 1,2,3,4,6,7,8-HpCDD	Heptachlorinated dioxin; persistent, bioaccumulative, toxic contaminant.
	LBXD07LA– 1,2,3,4,6,7,8,9-Octachlorinated dioxin Lipid Adj (pg/g)	Dioxin6-1,2,3,4,6,7,8,9-OCDD	Octachlorinated dioxin (OCDD); highly persistent, bioaccumulative, toxic environmental pollutant.
	LBXTCDLA– 2,3,7,8-Octachlorinated dioxin Lipid Adj (pg/g)	Dioxin7- 2,3,7,8-TCDD	Tetrachlorodibenzo-p-dioxin (TCDD); extremely toxic, carcinogenic, persistent pollutant.
Furans (PCDFs)	LBXF01LA– 2,3,7,8-Tetrachloro dibenzofuran Lipid Adj (pg/g)	Furan1- 2,3,7,8-TCDF	Tetrachlorodibenzofuran (TCDF); toxic, bioaccumulative, environmentally persistent.
	LBXF02LA– 1,2,3,7,8-Pentachlorinated dibenzofuran Lipid Adj (pg/g)	Furan2- 1,2,3,7,8-PeCDF	Pentachlorinated dibenzofuran; highly toxic, persistent contaminant.
	LBXF03LA– 2,3,4,7,8-Pentachlorinated dibenzofuran Lipid Adj (pg/g)	Furan3- 2,3,4,7,8-PeCDF	Pentachlorinated dibenzofuran; toxic, persistent environmental pollutant.
	LBXF04LA– 1,2,3,4,7,8-Hexachlorinated dibenzofuran Lipid Adj (pg/g)	Furan4- 1,2,3,4,7,8-HxCDF	Hexachlorinated dibenzofuran; persistent, bioaccumulative, toxic pollutant.
	LBXF05LA– 1,2,3,6,7,8-Hexachlorinated dibenzofuran Lipid Adj (pg/g)	Furan5- 1,2,3,6,7,8-HxCDF	Hexachlorinated dibenzofuran; toxic, persistent, bioaccumulative contaminant.
	LBXF06LA– 1,2,3,7,8,9-Hexachlorinated dibenzofuran Lipid Adj (pg/g)	Furan6- 1,2,3,7,8,9-HxCDF	Hexachlorinated dibenzofuran; persistent pollutant with significant toxicity.
	LBXF07LA– 2,3,4,6,7,8-Hexachlorinated dibenzofuran Lipid Adj (pg/g)	Furan7- 2,3,4,6,7,8-HxCDF	Hexachlorinated dibenzofuran; toxic, persistent, bioaccumulative chemical.
	LBXF08LA– 1,2,3,4,6,7,8-Heptachlorinated dibenzofuran Lipid Adj (pg/g)	Furan8- 1,2,3,4,6,7,8-HpCDF	Heptachlorinated dibenzofuran; persistent, bioaccumulative, toxic contaminant.
	LBXF09LA– 1,2,3,4,7,8,9-Heptachlorinated dibenzofuran Lipid Adj (pg/g)	Furan9- 1,2,3,4,7,8,9-HpCDF	Heptachlorinated dibenzofuran; persistent, toxic environmental pollutant.
	LBXF10LA– 1,2,3,4,6,7,8,9-Octachlorinated dibenzofuran Lipid Adj (pg/g)	Furan10-1,2,3,4,6,7,8,9-OCDF	Octachlorinated dibenzofuran (OCDF); highly chlorinated, persistent pollutant.

## References

[1] Martin S.S., Aday A.W., Almarzooq Z.I., Anderson C.A., Arora P., Avery C.L., Baker-Smith C.M., Barone Gibbs B., Beaton A.Z., Boehme A.K. (2024). 2024 Heart disease and stroke statistics: A report of US and global data from the American Heart Association. Circulation.

[2] Greenfield D.M., Snowden J.A. (2018). Cardiovascular Diseases and Metabolic Syndrome. The EBMT Handbook.

[3] Carreras E., Dufour C., Mohty M., Kröger N. (2019). The EBMT Handbook: Hematopoietic Stem Cell Transplantation and Cellular Therapies.

[4] Al-Masaoodi R.A., Saleh J.M., Al-Shemery M.K. (2021). Determination of Cardiovascular Diseases (CVD) with Importance Biomarkers: Review.

[5] Hajar R. (2016). Framingham Contribution to Cardiovascular Disease. Heart Views Off. J. Gulf Heart Assoc..

[6] Kelishadi R., Poursafa P. (2014). A review on the genetic, environmental, and lifestyle aspects of the early-life origins of cardiovascular disease. Curr. Probl. Pediatr. Adolesc. Health Care.

[7] Uhlig K., Levey A.S., Sarnak M.J. (2003). The clinical epidemiology of cardiovascular diseases in chronic kidney disease: Traditional Cardiac Risk Factors in Individuals with Chronic Kidney Disease. Semin. Dial..

[8] Everson-Rose S.A., Lewis T.T. (2005). Psychosocial factors and cardiovascular diseases. Annu. Rev. Public Health.

[9] Okereke O.I., Manson J.E. (2017). Psychosocial Factors and Cardiovascular Disease Risk. Circ. Res..

[10] Steptoe A., Kivimäki M. (2012). Stress and cardiovascular disease. Nat. Rev. Cardiol..

[11] Dupre M.E., Nelson A., Lynch S.M., Granger B.B., Xu H., Churchill E., Willis J.M., Curtis L.H., Peterson E.D. (2017). Socioeconomic, Psychosocial and Behavioral Characteristics of Patients Hospitalized With Cardiovascular Disease. Am. J. Med. Sci..

[12] Papageorgiou N. (2016). Cardiovascular Diseases: Genetic Susceptibility, Environmental Factors and Their Interaction.

[13] Khera A.V., Kathiresan S. (2017). Genetics of coronary artery disease: Discovery, biology and clinical translation. Nat. Rev. Genet..

[14] Forman D., Bulwer B.E. (2006). Cardiovascular disease: Optimal approaches to risk factor modification of diet and lifestyle. Curr. Treat. Options Cardiovasc. Med..

[15] Kashuba R., Menzie C., Martin L. (2021). Risk of cardiovascular disease is driven by different combinations of environmental, medical and behavioral factors: Building a conceptual model for cumulative risk assessment. Hum. Ecol. Risk Assess. Int. J..

[16] Reddy K.S., Katan M.B. (2004). Diet, nutrition and the prevention of hypertension and cardiovascular diseases. Public Health Nutr..

[17] Samuel P.O., Edo G.I., Emakpor O.L., Oloni G.O., Ezekiel G.O., Essaghah A.E.A., Agoh E., Agbo J.J. (2024). Lifestyle modifications for preventing and managing cardiovascular diseases. Sport Sci. Health.

[18] Mozaffarian D. (2016). Dietary and policy priorities for cardiovascular disease, diabetes, and obesity: A comprehensive review. Circulation.

[19] Gratas-Delamarche A., Derbré F., Vincent S., Cillard J. (2014). Physical inactivity, insulin resistance, and the oxidative-inflammatory loop. Free Radic. Res..

[20] Muniyappa R., Sowers J.R. (2013). Role of insulin resistance in endothelial dysfunction. Rev. Endocr. Metab. Disord..

[21] Chen W., Zhang S., Hu X., Chen F., Li D. (2023). A Review of Healthy Dietary Choices for Cardiovascular Disease: From Individual Nutrients and Foods to Dietary Patterns. Nutrients.

[22] Russo P., Milani F., De Iure A., Proietti S., Limongi D., Prezioso C., Checconi P., Zagà V., Novazzi F., Maggi F. (2024). Effect of cigarette smoking on clinical and molecular endpoints in COPD patients. Int. J. Mol. Sci..

[23] Xu L., Zimmermann M., Forkey H., Griffin J., Wilds C., Morgan W.S., Byatt N., McNeal C.J. (2022). How to mitigate risk of premature cardiovascular disease among children and adolescents with mental health conditions. Curr. Atheroscler. Rep..

[24] Ramos Meyers G., Samouda H., Bohn T. (2022). Short chain fatty acid metabolism in relation to gut microbiota and genetic variability. Nutrients.

[25] Hayat M., Kerr R., Bentley A.R., Rotimi C.N., Raal F.J., Ramsay M. (2020). Genetic associations between serum low LDL-cholesterol levels and variants in LDLR, APOB, PCSK9 and LDLRAP1 in African populations. PLoS ONE.

[26] Kathiresan S., Srivastava D. (2012). Genetics of Human Cardiovascular Disease. Cell.

[27] Zhang Y.-T., Zheng Y., Lin W.-H., Zhang H., Zhou X.-L. (2013). Challenges and Opportunities in Cardiovascular Health Informatics. IEEE Trans. Biomed. Eng..

[28] DeVon H.A., Vuckovic K.M., Ryan C.J., Barnason S., Zerwic J.J., Pozehl B.J., Schulz P.S., Seo Y., Zimmerman L. (2017). Systematic review of symptom clusters in cardiovascular disease. Eur. J. Cardiovasc. Nurs..

[29] de Lacerda D.A., Honorato P.F., Lima L.L.L., Oliveira A.V.L., de Lira E.A., de Lira F.E.A., de Sousa Neto J.F., de Lacerda M.A., Siqueira T.d.O., Braga T.R.O. Challenges in the early diagnosis of cardiovascular diseases in primary care in Brazil: Analysis and proposals for solutions. Proceedings of the VI Seven International Multidisciplinary Congress.

[30] Shetty S.S., Deepthi D., Harshitha S., Sonkusare S., Naik P.B., Madhyastha H. (2023). Environmental pollutants and their effects on human health. Heliyon.

[31] Timothy N.A., Williams E.T. (2019). Environmental Pollution by Heavy Metal: An Overview. Int. J. Environ. Chem..

[32] Ibadi E.A., Awad H.K., Hussain L.I. (2024). Environmental toxins and their effects on A comprehensive review of exposure: Human organs and their accumulation. J. Agric. Environ. Vet. Sci..

[33] Hussain C.M., Keçili R. (2020). Environmental pollution and environmental analysis. Modern Environmental Analysis Techniques for Pollutants.

[34] Agency for Toxic Substances and Disease Registry (ATSDR) (2020). Toxicological Profile for Lead.

[35] Tchounwou P.B., Yedjou C.G., Patlolla A.K., Sutton D.J. (2012). Heavy metal toxicity and the environment. Exp. Suppl..

[36] Agency for Toxic Substances and Disease Registry (ATSDR) (2012). Toxicological Profile for Cadmium.

[37] Agency for Toxic Substances and Disease Registry (ATSDR) (2022). Toxicological Profile for Mercury.

[38] Schwartz J. (1991). Lead, blood pressure, and cardiovascular disease in men and women. Environ. Health Perspect..

[39] Gidlow D.A. (2015). Lead toxicity. Occup. Med..

[40] Collin M.S., Venkatraman S.K., Vijayakumar N., Kanimozhi V., Arbaaz S.M., Stacey R.S., Anusha J., Choudhary R., Lvov V., Tovar G.I. (2022). Bioaccumulation of lead (Pb) and its effects on human: A review. J. Hazard. Mater. Adv..

[41] Swaringen B.F., Gawlik E., Kamenov G.D., McTigue N.E., Cornwell D.A., Bonzongo J.-C.J. (2022). Children’s exposure to environmental lead: A review of potential sources, blood levels, and methods used to reduce exposure. Environ. Res..

[42] Yu Y.-L., Yang W.-Y., Hara A., Asayama K., Roels H.A., Nawrot T.S., Staessen J.A. (2023). Public and occupational health risks related to lead exposure updated according to present-day blood lead levels. Hypertens. Res..

[43] Okpogba A.N., Ogbodo E.C., Amah U.K., Mounmbegna E.P., Obi-Ezeani C.N., Iwuji J.C. (2020). Evaluation of some heavy metal levels in blood of lead acid battery manufacturing factory workers in Nnewi, Nigeria. Indian J. Pharm. Pharmacol..

[44] Obeng-Gyasi E. (2019). Sources of lead exposure in various countries. Rev. Environ. Health.

[45] Jyothi N.R., Farook N.A.M. (2020). Mercury toxicity in public health. Heavy Metal Toxicity in Public Health.

[46] Zafar A., Javed S., Akram N., Naqvi S.A.R. (2024). Health risks of mercury. Mercury Toxicity Mitigation: Sustainable Nexus Approach.

[47] Mishra S., Bharagava R.N., More N., Yadav A., Zainith S., Mani S., Chowdhary P. (2018). Heavy metal contamination: An alarming threat to environment and human health. Environmental Biotechnology: For Sustainable Future.

[48] Committee E.S. (2015). Statement on the benefits of fish/seafood consumption compared to the risks of methylmercury in fish/seafood. EFSA J..

[49] Jannetto P.J., Cowl C.T. (2023). Elementary Overview of Heavy Metals. Clin. Chem..

[50] Hong Y.-S., Kim Y.-M., Lee K.-E. (2012). Methylmercury exposure and health effects. J. Prev. Med. Public Health.

[51] Grandjean P., Satoh H., Murata K., Eto K. (2010). Adverse effects of methylmercury: Environmental health research implications. Environ. Health Perspect..

[52] Lawal K.K., Ekeleme I.K., Onuigbo C.M., Ikpeazu V.O., Obiekezie S.O. (2021). A review on the public health implications of heavy metals. World J. Adv. Res. Rev..

[53] Penteado J.C.P., Vaz J.M. (2001). O legado das bifenilas policloradas (PCBs) (The legacy of polychlorinated biphenyls (PCBs)). Química Nova.

[54] Agency for Toxic Substances and Disease Registry (ATSDR) (2000). Toxicological Profile for Polychlorinated Biphenyls (PCBs).

[55] Biziuk M., Beyer A. (2020). Polychlorinated Biphenyls (PCBs). Managing Global Resources and Universal Processes.

[56] Ododo M.M., Wabalo B.K. (2019). Polychlorinated Biphenyls (PCBs) and Their Impacts on Human Health: A Review. J. Environ. Pollut. Hum. Health.

[57] Rathoure A.K. (2018). Dioxins: Source, origin and toxicity assessment. Biodivers. Int. J..

[58] Agency for Toxic Substances and Disease Registry (ATSDR) (2024). Toxicological Profile for Chlorinated Dibenzo-p-Dioxins.

[59] Kamrin M.A., Rodgers P.W. (1985). Dioxins in the Environment.

[60] Marinković N., Pašalić D., Ferenčak G., Gršković B., Rukavina A.S. (2010). Dioxins and Human Toxicity. Arh. Za Hig. Rada Toksikol..

[61] Banerjee R., Banerjee M. (2020). Medicinal significance of furan derivatives: A Review. Int. J. Rev. Life Sci..

[62] Agency for Toxic Substances and Disease Registry (ATSDR) (2023). Toxicological Profile for Chlorodibenzofurans (CDFs).

[63] De B., Sen S., Easwari T.S. (2015). Chemistry and Therapeutic Aspect of Furan: A Short Review. Asian J. Res. Chem..

[64] Seok Y.-J., Her J.-Y., Kim Y.-G., Kim M.Y., Jeong S.Y., Kim M.K., Lee J.Y., Kim C.-I., Yoon H.-J., Lee K.-G. (2015). Furan in Thermally Processed Foods—A Review. Toxicol. Res..

[65] Maga J.A. (1979). Furans in foods. CRC Crit. Rev. Food Sci. Nutr..

[66] Petrosino V., Motta G., Tenore G.C., Coletta M.A., Guariglia A., Testa D. (2018). The role of heavy metals and polychlorinated biphenyls (PCBs) in the oncogenesis of head and neck tumors and thyroid diseases: A pilot study. Biometals.

[67] Singh N., Gupta V.K., Kumar A., Sharma B. (2017). Synergistic Effects of Heavy Metals and Pesticides in Living Systems. Front. Chem..

[68] Matés J.M., Segura J.A., Alonso F.J., Márquez J. (2010). Roles of dioxins and heavy metals in cancer and neurological diseases using ROS-mediated mechanisms. Free Radic. Biol. Med..

[69] Bopp S.K., Barouki R., Brack W., Dalla Costa S., Dorne J.C.M., Drakvik P.E., Faust M., Karjalainen T.K., Kephalopoulos S., van Klaveren J. (2018). Current EU research activities on combined exposure to multiple chemicals. Environ. Int..

[70] Fu X., Xu J., Zhang R., Yu J. (2020). The association between environmental endocrine disruptors and cardiovascular diseases: A systematic review and meta-analysis. Environ. Res..

[71] Bashir T., Obeng-Gyasi E. (2022). Interaction of per-and polyfluoroalkyl substances and allostatic load among adults in various occupations. Diseases.

[72] Zarerad E., Niksalehi K., Armandeh M., Sani M.A., Ataei M., Mousavi T., Maghsoudi A.S., Hassani S. (2023). Polychlorinated Biphenyls: A Review of Recent Updates on Food Safety and Environmental Monitoring, Health and Toxicological Implications, and Analysis. Mini Rev. Med. Chem..

[73] Lee H.K., Pak Y.K. (2018). Persistent Organic Pollutants, Mitochondrial Dysfunction, and Metabolic Syndrome. Mitochondrial Dysfunction Caused by Drugs and Environmental Toxicants.

[74] Igwe E., Onoja S., Nwodo P., Baharane V., Diakite S., Saquee F., Ugwu B., Amechi O., Niambe O., Shaibu O. (2023). Identification of Sources of Some Priority Heavy Metallic Pollutants Caus-ing Environmental Degradation and It’s Health Implications. J. Ind. Pollut. Control.

[75] Pant A.B. (2024). Nanotoxicology. Dictionary of Toxicology.

[76] Lind P.M., Lind L. (2020). Are persistent organic pollutants linked to lipid abnormalities, atherosclerosis and cardiovascular disease? A review. J. Lipid Atheroscler..

[77] Raghavan A., Pirruccello J.P., Ellinor P.T., Lindsay M.E. (2024). Using genomics to identify novel therapeutic targets for aortic disease. Arterioscler. Thromb. Vasc. Biol..

[78] National Health and Nutrition Examination Survey NHANES 2003/2004. https://wwwn.cdc.gov/nchs/nhanes/continuousnhanes/default.aspx?BeginYear=2003.

[79] Caussy D., Gochfeld M., Gurzau E., Neagu C., Ruedel H. (2003). Lessons from case studies of metals: Investigating exposure, bioavailability, and risk. Ecotoxicol. Environ. Saf..

[80] Kanan S., Samara F. (2018). Dioxins and furans: A review from chemical and environmental perspectives. Trends Environ. Anal. Chem..

[81] Rice C.P., O’Keefe P., Kubiak T. (2002). Sources, pathways, and effects of PCBs, dioxins, and dibenzofurans. Handbook of Ecotoxicology.

[82] Evans R.M., Martin O.V., Faust M., Kortenkamp A. (2016). Should the scope of human mixture risk assessment span legislative/regulatory silos for chemicals?. Sci. Total Environ..

[83] Yip F., Christensen B., Sircar K., Naeher L., Bruce N., Pennise D., Lozier M., Pilishvili T., Loo Farrar J., Stanistreet D. (2017). Assessment of traditional and improved stove use on household air pollution and personal exposures in rural western Kenya. Environ. Int..

[84] Mahaffey K.R. (2004). Fish and shellfish as dietary sources of methylmercury and the ω-3 fatty acids, eicosahexaenoic acid and docosahexaenoic acid: Risks and benefits. Environ. Res..

[85] Downward G.S., Hu W., Large D., Veld H., Xu J., Reiss B., Wu G., Wei F., Chapman R.S., Rothman N. (2014). Heterogeneity in coal composition and implications for lung cancer risk in Xuanwei and Fuyuan counties, China. Environ. Int..

[86] Akagi H., Grandjean P., Takizawa Y., Weihe P. (1998). Methylmercury Dose Estimation from Umbilical Cord Concentrations in Patients with Minamata Disease. Environ. Res..

[87] Baishaw S., Edwards J., Daughtry B., Ross K. (2007). Mercury in seafood: Mechanisms of accumulation and consequences for consumer health. Rev. Environ. Health.

[88] Abballe A., Barbieri P.G., di Domenico A., Garattini S., Iacovella N., Ingelido A.M., Marra V., Miniero R., Valentini S., De Felip E. (2013). Occupational exposure to PCDDs, PCDFs, and PCBs of metallurgical workers in some industrial plants of the Brescia area, northern Italy. Chemosphere.

[89] Montano L., Pironti C., Pinto G., Ricciardi M., Buono A., Brogna C., Venier M., Piscopo M., Amoresano A., Motta O. (2022). Polychlorinated biphenyls (PCBs) in the environment: Occupational and exposure events, effects on human health and fertility. Toxics.

[90] Jacobs D.E., Clickner R.P., Zhou J.Y., Viet S.M., Marker D.A., Rogers J.W., Zeldin D.C., Broene P., Friedman W. (2002). The prevalence of lead-based paint hazards in U.S. housing. Environ. Health Perspect..

[91] David O. (2006). Carpenter. Polychlorinated Biphenyls (PCBs): Routes of Exposure and Effects on Human Health. Rev. Environ. Health.

[92] Asadollahi-Baboli M. (2012). Exploring QSTR analysis of the toxicity of phenols and thiophenols using machine learning methods. Environ. Toxicol. Pharmacol..

[93] Luben T.J., Buckley B.J., Patel M.M., Stevens T., Coffman E., Rappazzo K.M., Owens E.O., Hines E.P., Moore D., Painter K. (2018). A cross-disciplinary evaluation of evidence for multipollutant effects on cardiovascular disease. Environ. Res..

[94] Seubert J.M., Kennedy C.J. (2000). Benzo[a]pyrene Toxicokinetics in Rainbow Trout (Oncorhynchus mykiss) Acclimated to Different Salinities. Arch. Environ. Contam. Toxicol..

[95] Mahaffey K.R., Clickner R.P., Jeffries R.A. (2009). Adult Women’s Blood Mercury Concentrations Vary Regionally in the United States: Association with Patterns of Fish Consumption (NHANES 1999–2004). Environ. Health Perspect..

[96] Vaye O., Ngumbu R.S., Xia D. (2022). A review of the application of comprehensive two-dimensional gas chromatography MS-based techniques for the analysis of persistent organic pollutants and ultra-trace level of organic pollutants in environmental samples. Rev. Anal. Chem..

[97] Schisterman E.F., Whitcomb B.W., Louis G.M.B., Louis T.A. (2005). Lipid Adjustment in the Analysis of Environmental Contaminants and Human Health Risks. Environ. Health Perspect..

[98] Lee E.-S.Y., Chen H., Soliman K.F.A., Charlton C.G. (2005). Effects of Homocysteine on the Dopaminergic System and Behavior in Rodents. NeuroToxicology.

[99] Baratta M., Jian W., Hengel S., Kaur S., Cunliffe J., Boer J., Hughes N., Kar S., Kellie J., Kim Y.J. (2024). 2023 White Paper on Recent Issues in Bioanalysis: Deuterated Drugs; LNP; Tumor/FFPE Biopsy; Targeted Proteomics; Small Molecule Covalent Inhibitors; Chiral Bioanalysis; Remote Regulatory Assessments; Sample Reconciliation/Chain of Custody (PART 1A—Recommendations on Mass Spectrometry, Chromatography, Sample Preparation Latest Developments, Challenges, and Solutions and BMV/Regulated Bioanalysis PART 1B—Regulatory Agencies’ Inputs on Regulated Bioanalysis/BMV, Biomarkers/IVD/CDx/BAV, Immunogenicity, Gene & Cell Therapy and Vaccine). Bioanalysis.

[100] Goff D.C., Lloyd-Jones D.M., Bennett G., Coady S., D’Agostino R.B., Gibbons R., Greenland P., Lackland D.T., Levy D., O’Donnell C.J. (2014). 2013 ACC/AHA Guideline on the Assessment of Cardiovascular Risk. JACC.

[101] D’Agostino R.B., Vasan R.S., Pencina M.J., Wolf P.A., Cobain M., Massaro J.M., Kannel W.B. (2008). General Cardiovascular Risk Profile for Use in Primary Care. Circulation.

[102] Yusuf S., Hawken S., Ôunpuu S., Dans T., Avezum A., Lanas F., McQueen M., Budaj A., Pais P., Varigos J. (2004). Effect of potentially modifiable risk factors associated with myocardial infarction in 52 countries (the INTERHEART study): Case-control study. Lancet.

[103] Stringhini S., Carmeli C., Jokela M., Avendaño M., Muennig P., Guida F., Ricceri F., d’Errico A., Barros H., Bochud M. (2017). Socioeconomic status and the 25 x 25 risk factors as determinants of premature mortality: A multicohort study and meta-analysis of 1.7 million men and women. Lancet.

[104] Mahmood S.S., Levy D., Vasan R.S., Wang T.J. (2014). The Framingham Heart Study and the epidemiology of cardiovascular disease: A historical perspective. Lancet.

[105] Bobb J.F., Valeri L., Claus Henn B., Christiani D.C., Wright R.O., Mazumdar M., Godleski J.J., Coull B.A. (2015). Bayesian kernel machine regression for estimating the health effects of multi-pollutant mixtures. Biostatistics.

[106] Boafo Y.S., Mostafa S., Obeng-Gyasi E. (2023). Association of Combined Metals and PFAS with Cardiovascular Disease Risk. Toxics.

[107] Chung M.K., House J.S., Akhtari F.S., Makris K.C., Langston M.A., Islam K.T., Holmes P., Chadeau-Hyam M., Smirnov A.I., Du X. (2024). Decoding the exposome: Data science methodologies and implications in exposome-wide association studies (ExWASs). Exposome.

[108] Yu H., Hutson A.D. (2022). A robust Spearman correlation coefficient permutation test. Communications in Statistics Theory Methods.

[109] Akinbode O.L., Obeng-Gyasi E. (2025). Combined Effects of Arsenic, Cadmium, and Mercury with Cardiovascular Disease Risk: Insights from the All of Us Research Program. Int. J. Environ. Res. Public Health.

[110] Zar J.H. (2005). Spearman rank correlation. Encyclopedia of Biostatistics.

[111] Reckelhoff J.F. (2001). Gender differences in the regulation of blood pressure. Hypertension.

[112] Silveyra P., Al Housseiny H., Rebuli M.E. (2021). Sex and gender differences in the susceptibility to environmental exposures. Sex-Based Differences in Lung Physiology.

[113] Connelly P.J., Azizi Z., Alipour P., Delles C., Pilote L., Raparelli V. (2021). The Importance of Gender to Understand Sex Differences in Cardiovascular Disease. Can. J. Cardiol..

[114] Pearce N. (2008). Epidemiology in Latin America: An opportunity for a global dialogue. J. Epidemiol. Community Health.

[115] Kaplan R.C., Avilés-Santa M.L., Parrinello C.M., Hanna D.B., Jung M., Castañeda S.F., Hankinson A.L., Isasi C.R., Birnbaum-Weitzman O., Kim R.S. (2014). Body Mass Index, Sex, and Cardiovascular Disease Risk Factors Among Hispanic/Latino Adults: Hispanic Community Health Study/Study of Latinos. J. Am. Heart Assoc..

[116] Egan K.B., Cornwell C.R., Courtney J.G., Ettinger A.S. (2021). Blood Lead Levels in U.S. Children Ages 1–11 Years, 1976–2016. Environ. Health Perspect..

[117] Morello-Frosch R., Shenassa E.D. (2006). The Environmental “Riskscape” and Social Inequality: Implications for Explaining Maternal and Child Health Disparities. Environ. Health Perspect..

[118] Smith M.W., Patterson N., Lautenberger J.A., Truelove A.L., McDonald G.J., Waliszewska A., Kessing B.D., Malasky M.J., Scafe C., Le E. (2004). A High-Density Admixture Map for Disease Gene Discovery in African Americans. Am. J. Hum. Genet..

[119] Wigle D.T. (2003). Child Health and the Environment.

[120] Kitayama S., Varnum M.E., Salvador C.E. (2019). Cultural neuroscience. Handbook of Cultural Psychology.

[121] Aminov Z., Haase R.F., Pavuk M., Carpenter D.O., Anniston Environmental Health Research Consortium (2013). Analysis of the effects of exposure to polychlorinated biphenyls and chlorinated pesticides on serum lipid levels in residents of Anniston, Alabama. Environ. Health.

[122] Goncharov A., Haase R.F., Santiago-Rivera A., Morse G., McCaffrey R.J., Rej R., Carpenter D.O. (2008). High serum PCBs are associated with elevation of serum lipids and cardiovascular disease in a Native American population. Environ. Res..

[123] Lind P.M., van Bavel B., Salihovic S., Lind L. (2012). Circulating levels of persistent organic pollutants (POPs) and carotid atherosclerosis in the elderly. Environ. Health Perspect..

[124] Everett C.J., Mainous A.G., Frithsen I.L., Player M.S., Matheson E.M. (2008). Association of polychlorinated biphenyls with hypertension in the 1999–2002 National Health and Nutrition Examination Survey. Environ. Res..

[125] O’Neill M.S., Diez-Roux A.V., Auchincloss A.H., Shen M., Lima J.A., Polak J.F., Barr R.G., Kaufman J., Jacobs D.R. (2011). Long-Term Exposure to Airborne Particles and Arterial Stiffness: The Multi-Ethnic Study of Atherosclerosis (MESA). Environ. Health Perspect..

[126] Avila-Alejo J.O., González-Palomo A.K., Plascencia-Villa G., José-Yacamán M., Navarro-Contreras H.R., Pérez-Maldonado I.N. (2017). Low cytotoxicity of anisotropic gold nanoparticles coated with lysine on peripheral blood mononuclear cells “in vitro”. Environ. Toxicol. Pharmacol..

[127] Adams S.V., Newcomb P.A. (2013). Urinary Cadmium as a Marker of Exposure in Epidemiological Studies. Environ. Health Perspect..

[128] Dominici F., Peng R.D., Barr C.D., Bell M.L. (2010). Protecting Human Health From Air Pollution: Shifting From a Single-pollutant to a Multipollutant Approach. Epidemiology.

[129] Vandenberg L.N., Colborn T., Hayes T.B., Heindel J.J., Jacobs D.R., Lee D.-H., Shioda T., Soto A.M., vom Saal F.S., Welshons W.V. (2012). Hormones and Endocrine-Disrupting Chemicals: Low-Dose Effects and Nonmonotonic Dose Responses. Endocr. Rev..

[130] Kortenkamp A. (2007). Ten Years of Mixing Cocktails: A Review of Combination Effects of Endocrine-Disrupting Chemicals. Environ. Health Perspect..

[131] Calabrese E.J., Baldwin L.A. (2003). Hormesis: The Dose-Response Revolution. Annu. Rev. Pharmacol. Toxicol..

[132] Carpenter D.O., Arcaro K., Spink D.C. (2002). Understanding the human health effects of chemical mixtures. Environ. Health Perspect..

[133] Lind L., Lind P.M. (2012). Can persistent organic pollutants and plastic-associated chemicals cause cardiovascular disease?. J. Intern. Med..

[134] Mostafalou S., Abdollahi M. (2013). Pesticides and human chronic diseases: Evidences, mechanisms, and perspectives. Toxicol. Appl. Pharmacol..

[135] Wisniewski P., Romano R.M., Kizys M.M.L., Oliveira K.C., Kasamatsu T., Giannocco G., Chiamolera M.I., Dias-da-Silva M.R., Romano M.A. (2015). Adult exposure to bisphenol A (BPA) in Wistar rats reduces sperm quality with disruption of the hypothalamic–pituitary–testicular axis. Toxicology.

[136] Bose-O’Reilly S., Lettmeier B., Gothe R.M., Beinhoff C., Siebert U., Drasch G. (2008). Mercury as a serious health hazard for children in gold mining areas. Environ. Res..

[137] Casals-Casas C., Desvergne B. (2011). Endocrine Disruptors: From Endocrine to Metabolic Disruption. Annu. Rev. Physiol..

[138] Bechi N., Ietta F., Romagnoli R., Focardi S., Corsi I., Buffi C., Paulesu L. (2006). Estrogen-Like Response to p-Nonylphenol in Human First Trimester Placenta and BeWo Choriocarcinoma Cells. Toxicol. Sci..

[139] Larkin A., Siddens L.K., Krueger S.K., Tilton S.C., Waters K.M., Williams D.E., Baird W.M. (2013). Application of a fuzzy neural network model in predicting polycyclic aromatic hydrocarbon-mediated perturbations of the Cyp1b1 transcriptional regulatory network in mouse skin. Toxicol. Appl. Pharmacol..

[140] Merrill M.L., Emond C., Kim M.J., Antignac J.-P., Bizec B.L., Clément K., Birnbaum L.S., Barouki R. (2013). Toxicological Function of Adipose Tissue: Focus on Persistent Organic Pollutants. Environ. Health Perspect..

[141] Vaziri N.D. (2008). Mechanisms of lead-induced hypertension and cardiovascular disease. Am. J. Physiol. Heart Circ. Physiol..

[142] Navas-Acien A., Guallar E., Silbergeld E.K., Rothenberg S.J. (2007). Lead exposure and cardiovascular disease--a systematic review. Environ. Health Perspect..

[143] Lamas G.A., Bhatnagar A., Jones M.R., Mann K.K., Nasir K., Tellez-Plaza M., Ujueta F., Navas-Acien A. (2023). Contaminant Metals as Cardiovascular Risk Factors: A Scientific Statement From the American Heart Association. J. Am. Heart Assoc. Cardiovasc. Cerebrovasc. Dis..

[144] Genchi G., Sinicropi M.S., Carocci A., Lauria G., Catalano A. (2017). Mercury Exposure and Heart Diseases. Int. J. Environ. Res. Public Health.

[145] Houston M.C. (2011). Role of mercury toxicity in hypertension, cardiovascular disease, and stroke. J. Clin. Hypertens..

[146] Tellez-Plaza M., Jones M.R., Dominguez-Lucas A., Guallar E., Navas-Acien A. (2013). Cadmium exposure and clinical cardiovascular disease: A systematic review. Curr. Atheroscler. Rep..

[147] Pavuk M., Serio T.C., Cusack C., Cave M., Rosenbaum P.F., Birnbaum L.S. (2019). Hypertension in Relation to Dioxins and Polychlorinated Biphenyls from the Anniston Community Health Survey Follow-Up. Environ. Health Perspect..

[148] Uemura H., Arisawa K., Hiyoshi M., Satoh H., Sumiyoshi Y., Morinaga K., Kodama K., Suzuki T., Nagai M., Suzuki T. (2008). Associations of environmental exposure to dioxins with prevalent diabetes among general inhabitants in Japan. Environ. Res..

[149] Melymuk L., Blumenthal J., Sáňka O., Shu-Yin A., Singla V., Šebková K., Pullen Fedinick K., Diamond M.L. (2022). Persistent Problem: Global Challenges to Managing PCBs. Environ. Sci. Technol..

[150] Singh K., Chan H.M. (2018). Association of blood polychlorinated biphenyls and cholesterol levels among Canadian Inuit. Environ. Res..

[151] Bertazzi P.A., Bernucci I., Brambilla G., Consonni D., Pesatori A.C. (1998). The Seveso studies on early and long-term effects of dioxin exposure: A review. Environ. Health Perspect..

[152] Lind P.M., Penell J., Salihovic S., van Bavel B., Lind L. (2014). Circulating levels of *p*,*p*′-DDE are related to prevalent hypertension in the elderly. Environ. Res..

[153] Koeth R.A., Wang Z., Levison B.S., Buffa J.A., Org E., Sheehy B.T., Britt E.B., Fu X., Wu Y., Li L. (2013). Intestinal microbiota metabolism of l-carnitine, a nutrient in red meat, promotes atherosclerosis. Nat. Med..

[154] Faroon O., Ruiz P. (2016). Polychlorinated biphenyls: New evidence from the last decade. Toxicol. Ind. Health.

[155] Czarnota J., Gennings C., Wheeler D.C. (2015). Assessment of Weighted Quantile Sum Regression for Modeling Chemical Mixtures and Cancer Risk. Cancer Inform..

