# Supplementary material for: Combined Effects of Metals, PCBs, Dioxins, and Furans on Cardiovascular Dysfunction
Source: J Xenobiot. 2025 Jun 19;15(3):94. doi: 10.3390/jox15030094 (PMC12194091; doi:10.3390/jox15030094)
Supplement: Supplementary file 1 [file jox-15-00094-s001.zip › jox-3677970-supplementary.pdf]

Combined Effects of Metals, PCBs, Dioxins, and Furans on Cardiovascular Dysfunction

Supplementary Materials

Supplementary Table S1: Descriptive statistics showing unadjusted (mean (S.D)) of outcome variables by gender

	**FRS**	**DBP (mmHg)**	**SBP (mmHg)**	**HDL CHOLESTEROL (mg/dL)**	**LDL CHOLESTEROL (mg/dL)**	**TOTAL CHOLESTEROL (mg/dL)**	**TRIGLYCERIDES (mg/dL)**
Overall	1.77 (8.19)	70.42 (12.45)	122.79 (19.87)	54.61 (16.20)	116.60 (36.57)	199.26 (40.16)	140.18 (72.63)
Male	1.76 (8.45)	71.58 (14.14)	124.98 (18.57)	48.55 (11.83)	122.44 (35.76)	199.16 (38.98)	140.77 (66.09)
Female	1.78 (7.99)	69.44 (10.76)	120.94 (20.77)	59.74 (17.58)	111.66 (36.60)	199.35 (41.20)	139.68 (77.87)

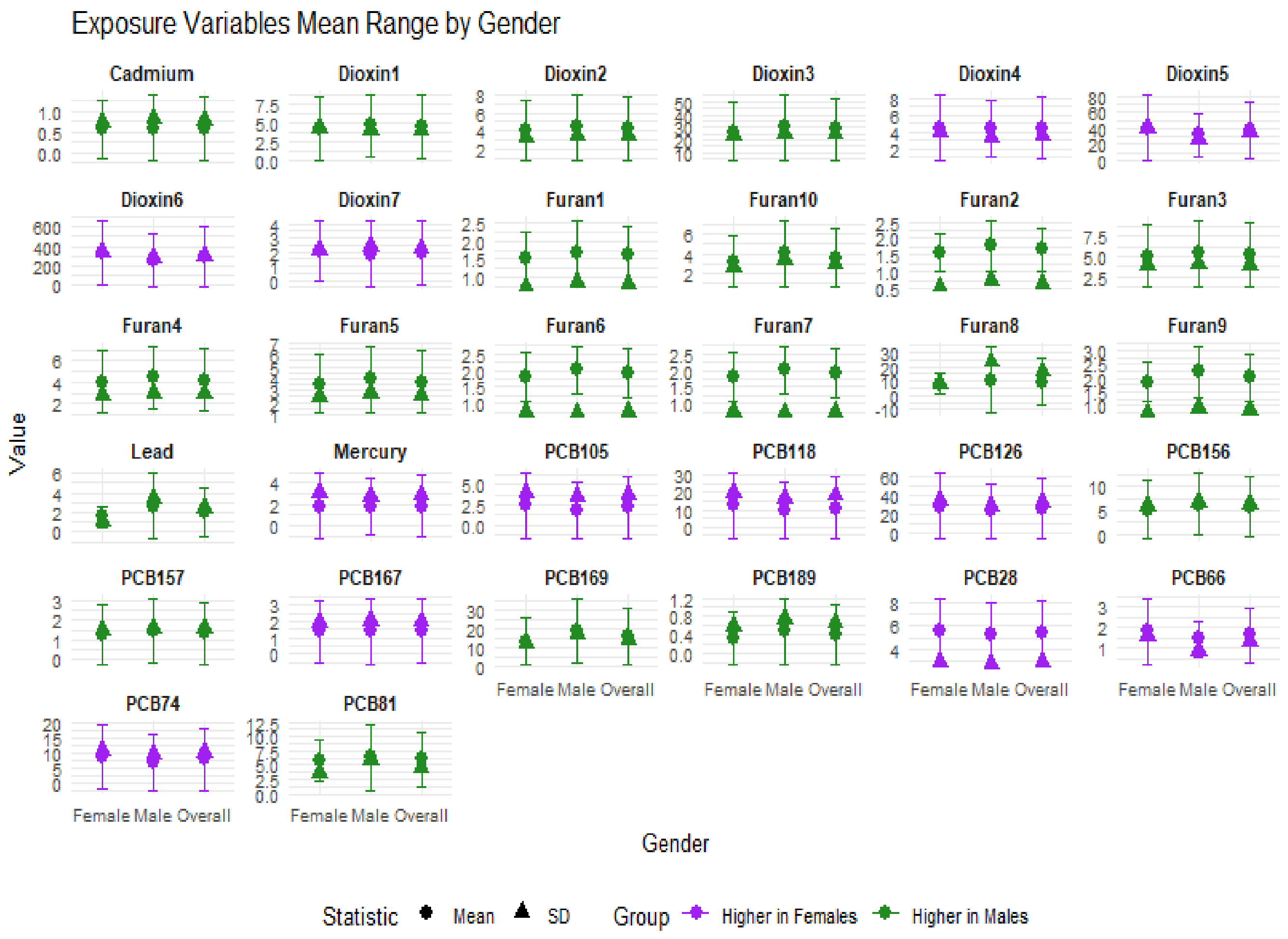

Supplementary Figure S1. Dot-plot visualization of the descriptive statistics summary of the exposures according to the highest mean value by gender.

DBP

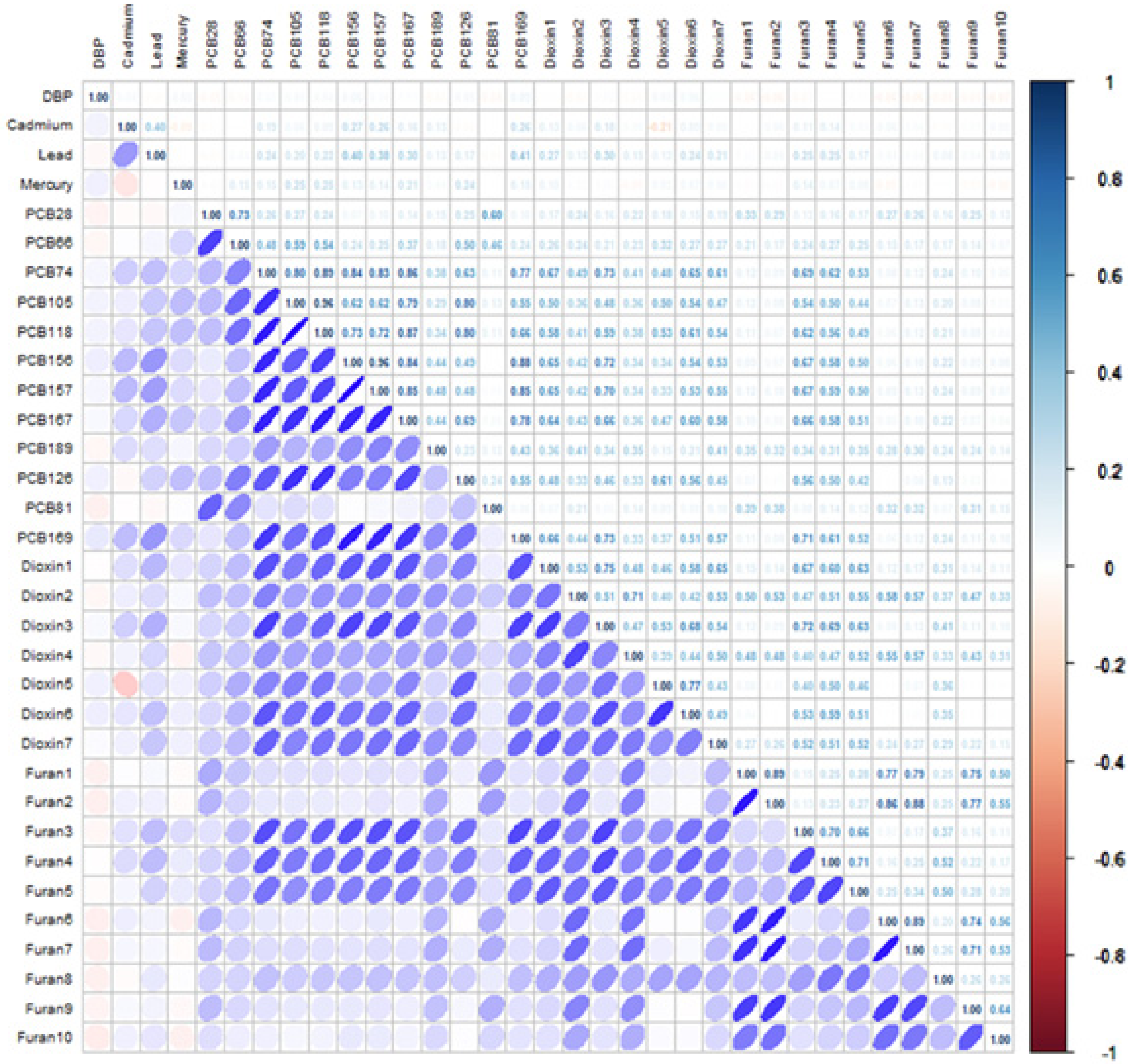

Supplementary Figure S2. Spearman correlation between pairs of metals, PCBs, Dioxins and Furans on Diastolic blood pressure DBP

SBP

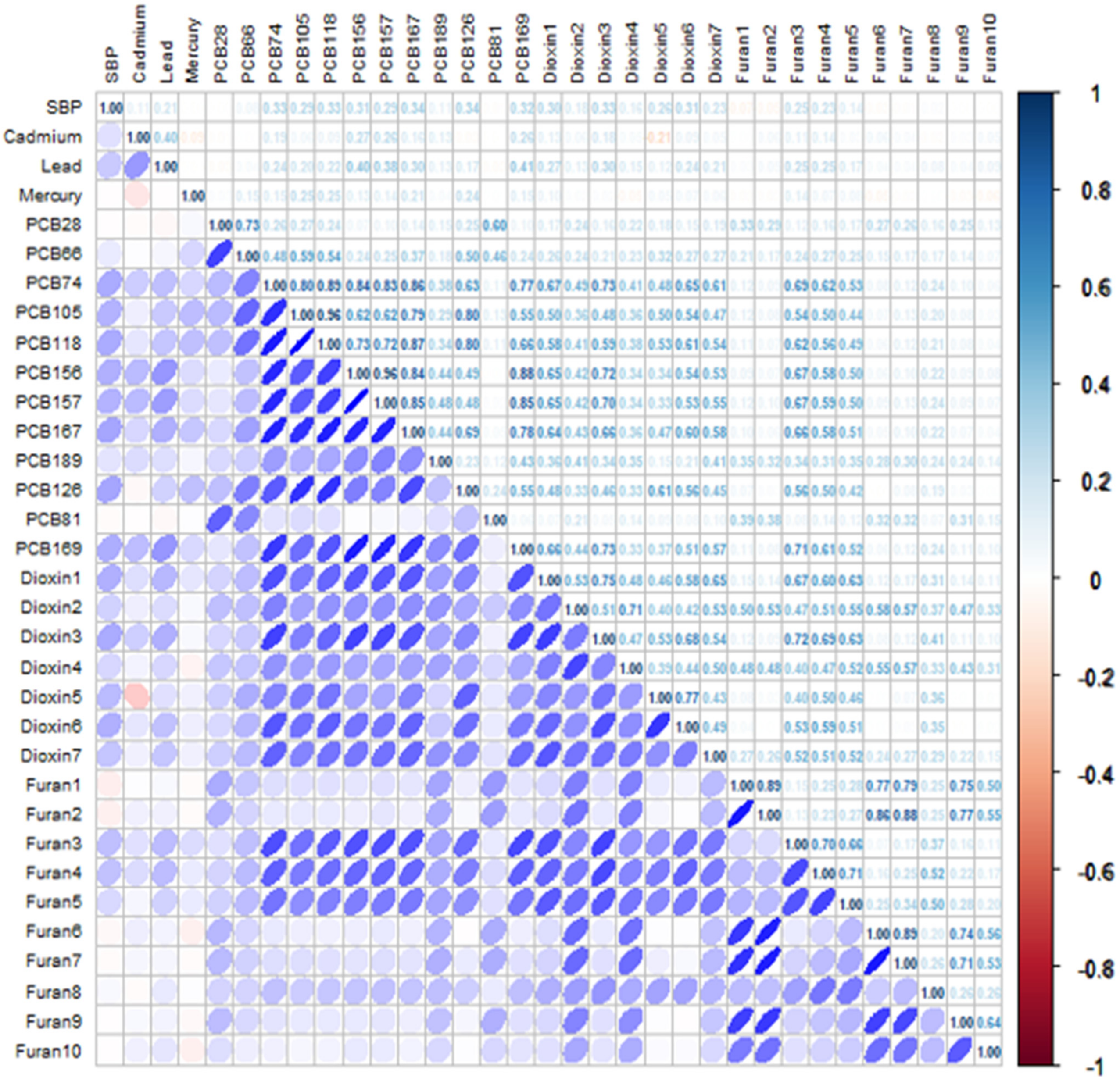

Supplementary Figure S3. Spearman correlation between pairs of metals, PCBs, Dioxins and Furans on Diastolic blood pressure SBP

HDL Cholesterol

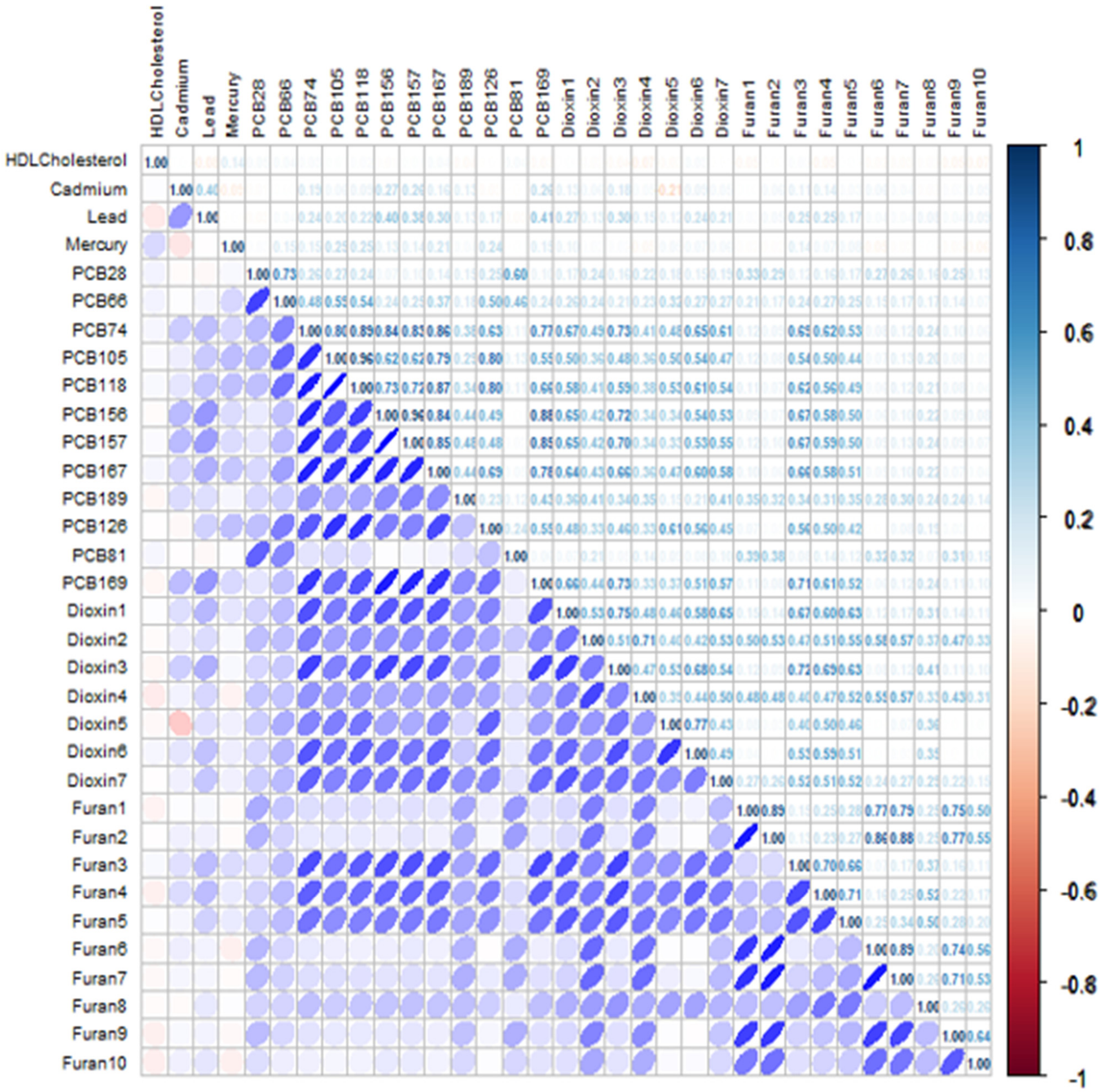

Supplementary Figure S4. Spearman correlation between pairs of metals, PCBs, Dioxins and Furans on Diastolic blood pressure HDL

LDL Cholesterol

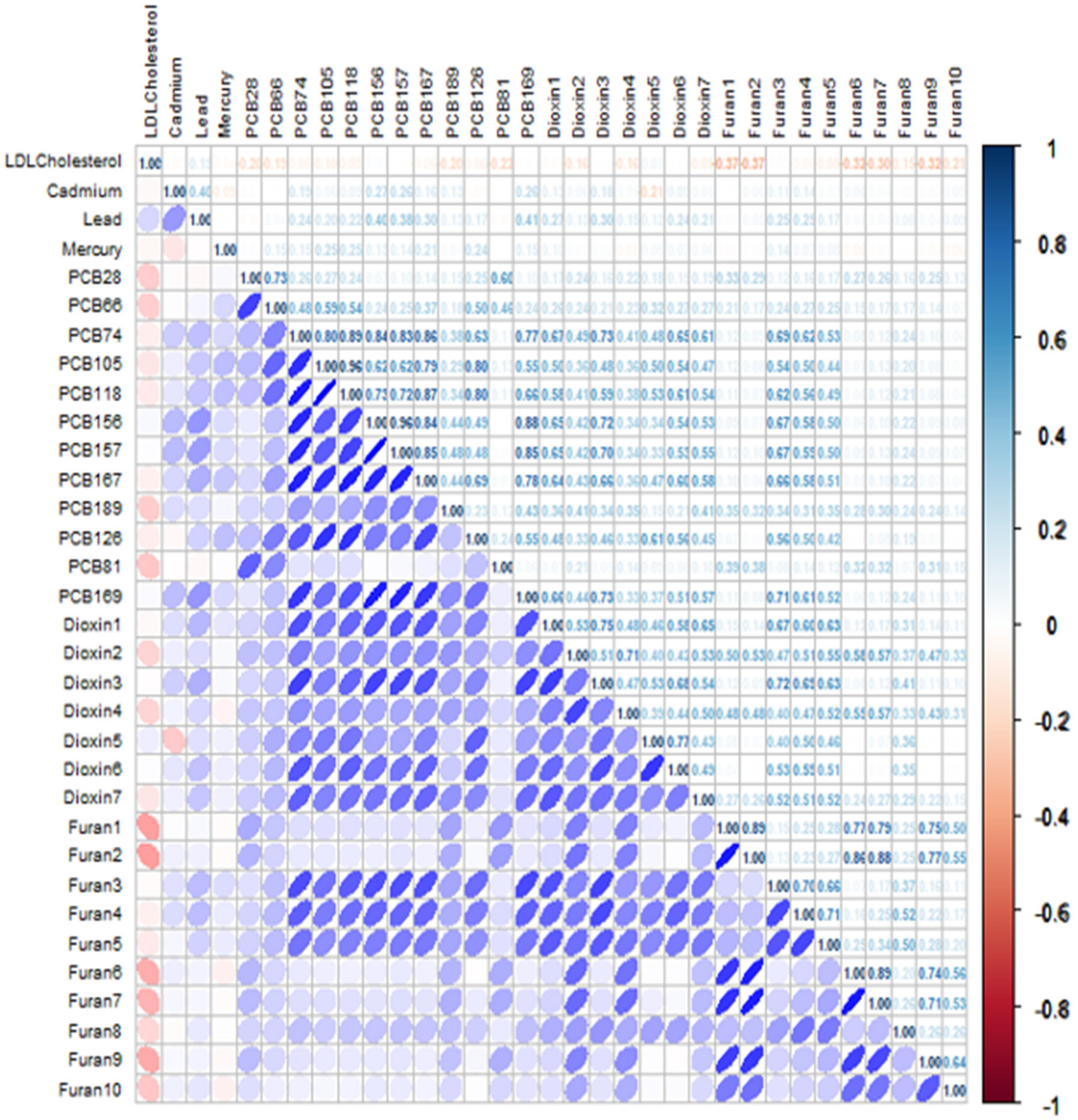

Supplementary Figure S5. Spearman correlation between pairs of metals, PCBs, Dioxins and Furans on Diastolic blood pressure LDL

TC

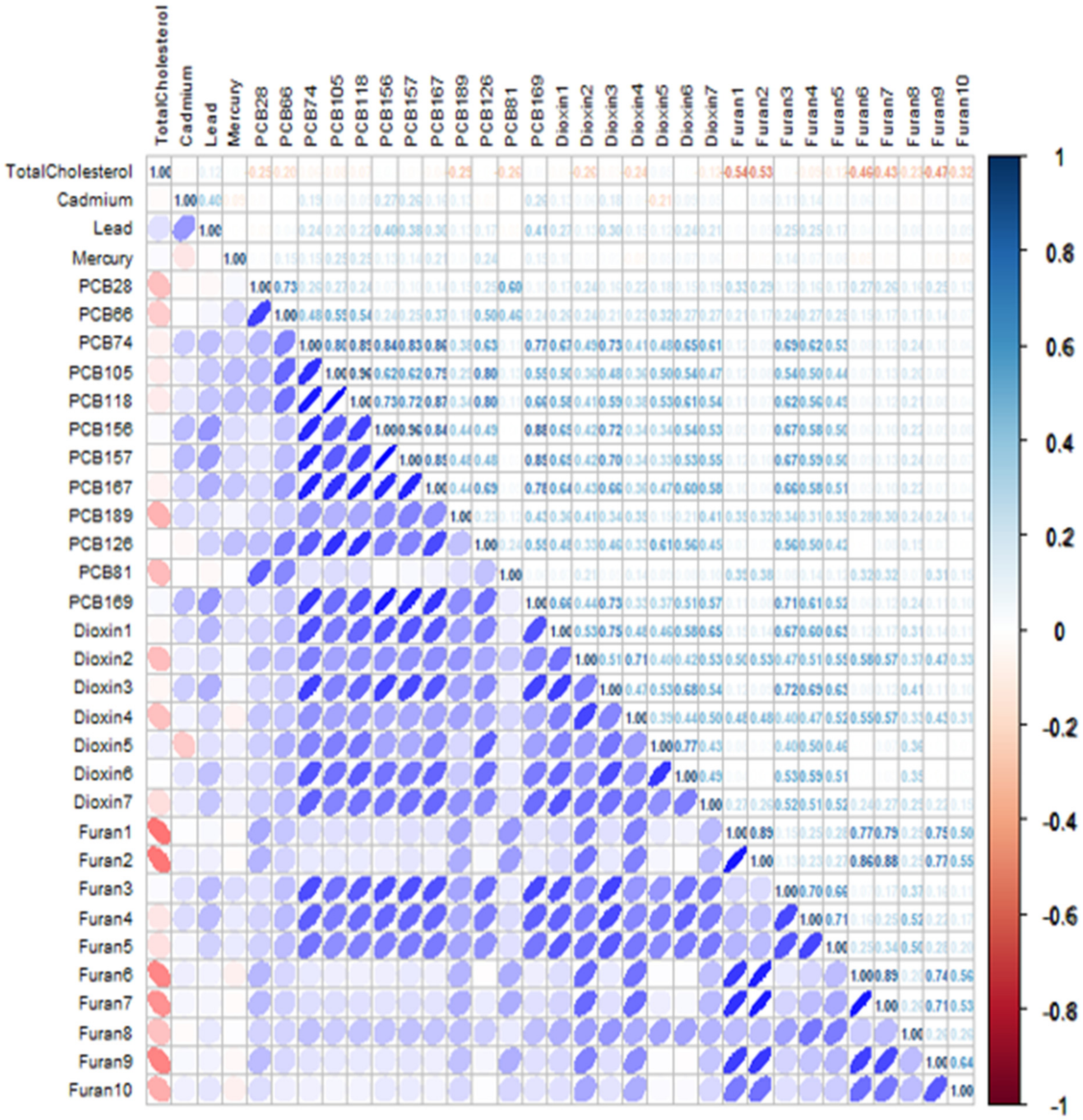

Supplementary Figure S6. Spearman correlation between pairs of metals, PCBs, Dioxins and Furans on Diastolic blood pressure TC

Triglycerides

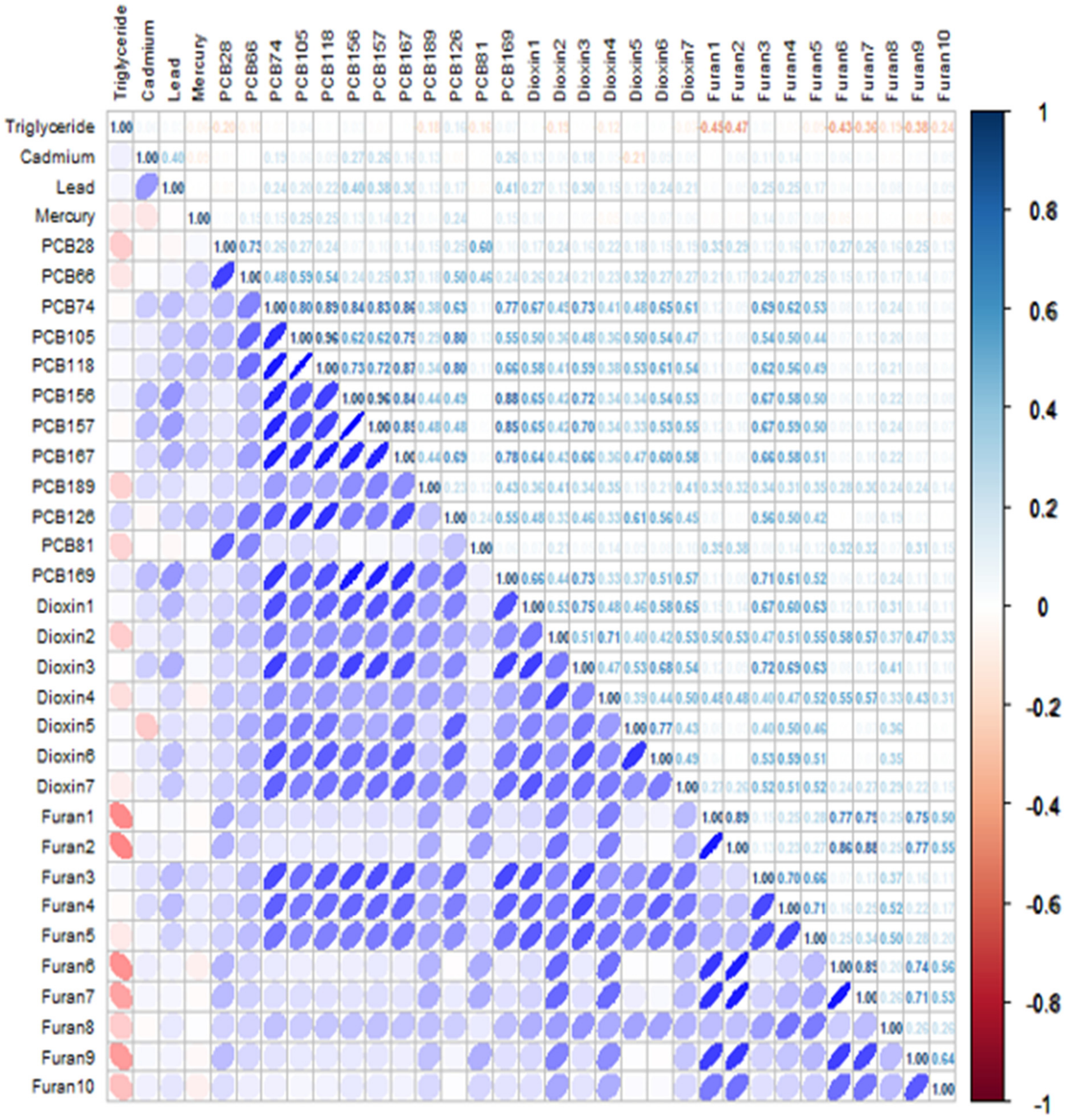

Supplementary Figure S7. Spearman correlation between pairs of metals, PCBs, Dioxins and Furans on Diastolic blood pressure Triglycerides

Univariate Exposure-Response relationship

Univariate exposure–response functions and 95% credible interval of metals, PCBs, Dioxins and Furans on CVD-related outcomes: DBP, SBP, HDL, LDL Cholesterol, Total Cholesterol, and Triglycerides. Adjusted for alcohol consumption, smoking status, age, ethnicity, income level, gender, and BMI.

DBP

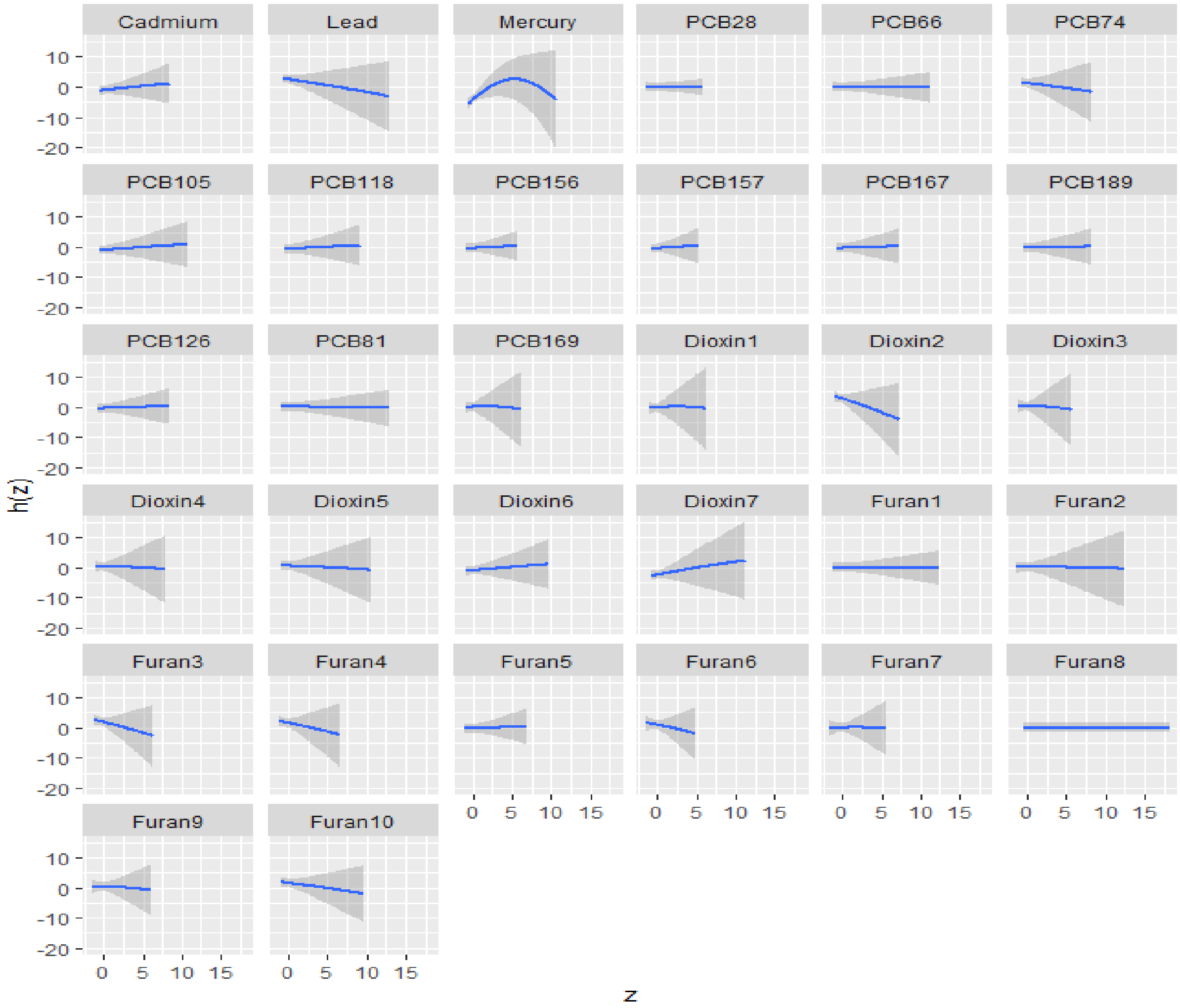

Supplementary Figure S8. Univariate exposure–response functions and 95% credible interval of metals, PCBs, Dioxins and Furans on CVD-related outcome: DBP.

SBP

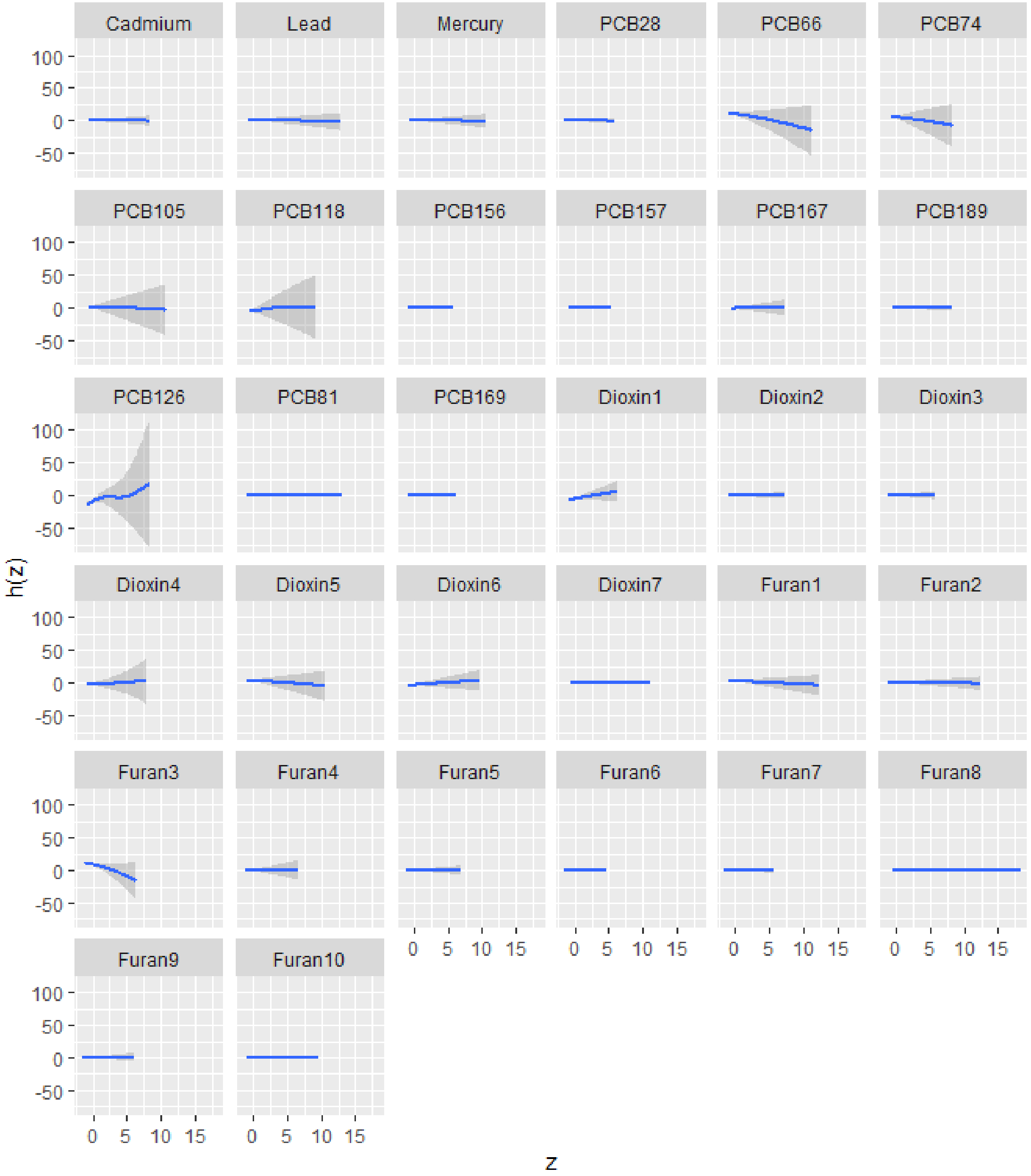

Supplementary Figure S9. Univariate exposure–response functions and 95% credible interval of metals, PCBs, Dioxins and Furans on CVD-related outcome: SBP.

HDL Cholesterol

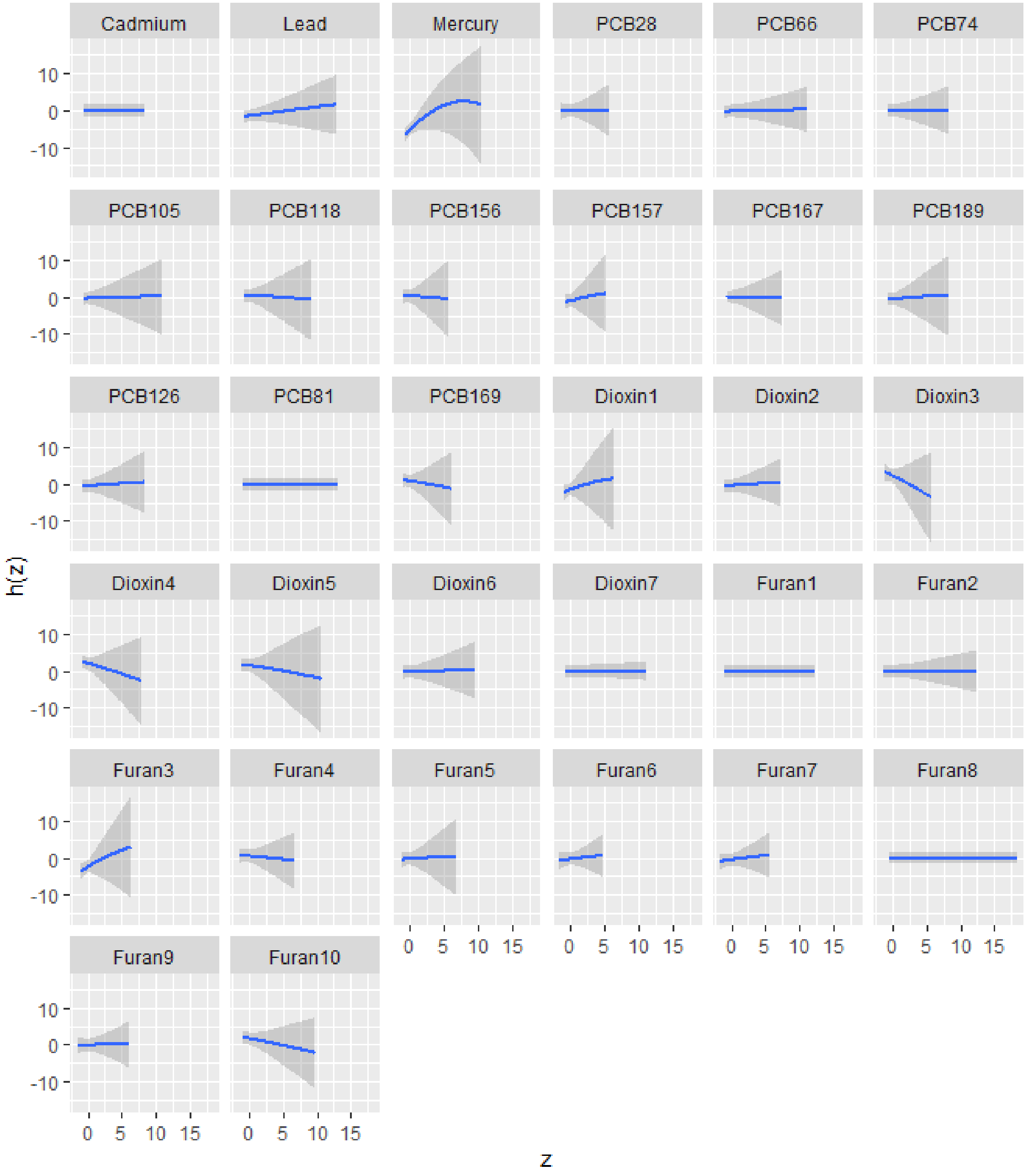

Supplementary Figure S10. Univariate exposure–response functions and 95% credible interval of metals, PCBs, Dioxins and Furans on CVD-related outcome: HDL.

LDL Cholesterol

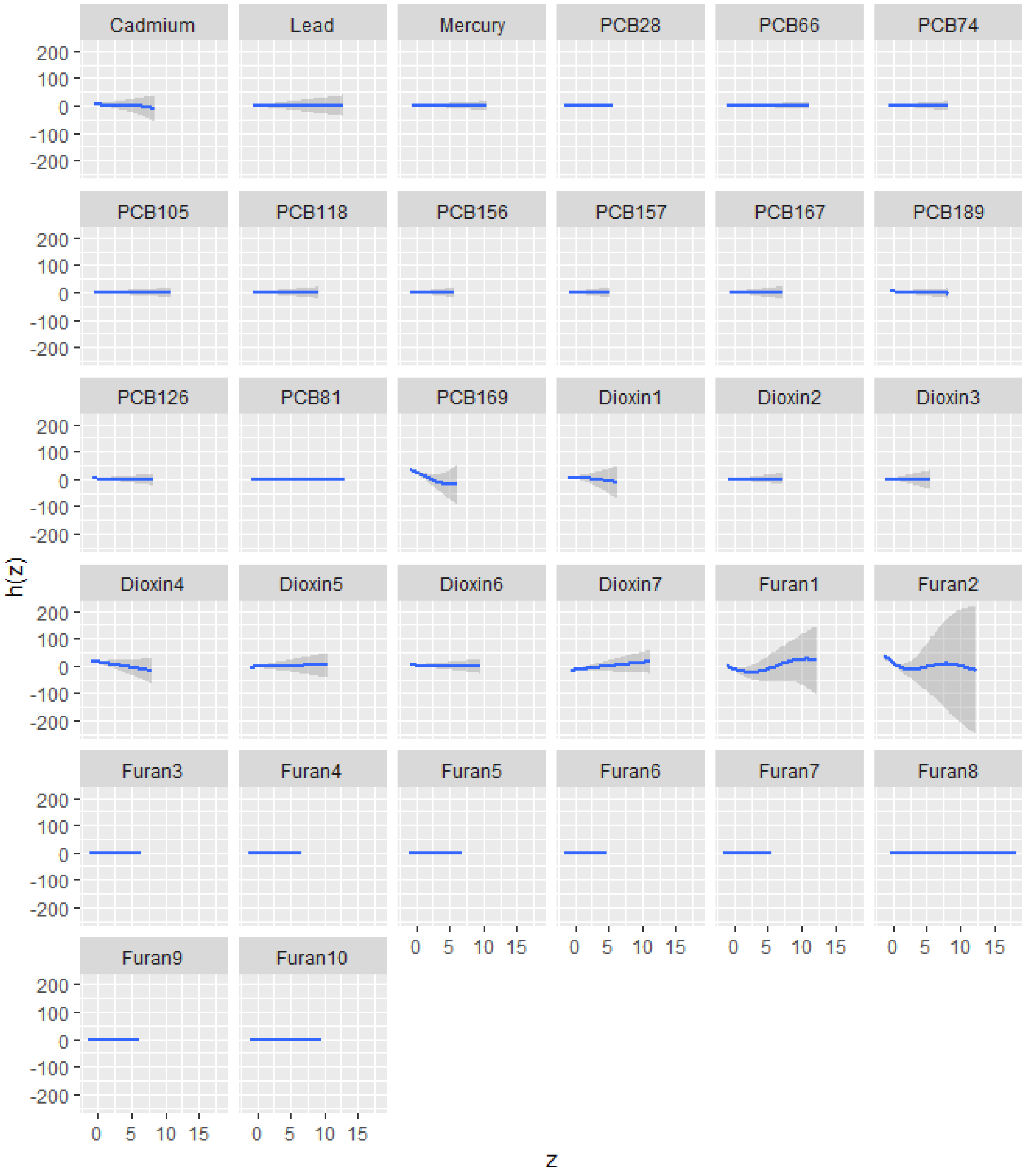

Supplementary Figure S11. Univariate exposure–response functions and 95% credible interval of metals, PCBs, Dioxins and Furans on CVD-related outcome: LDL.

TC

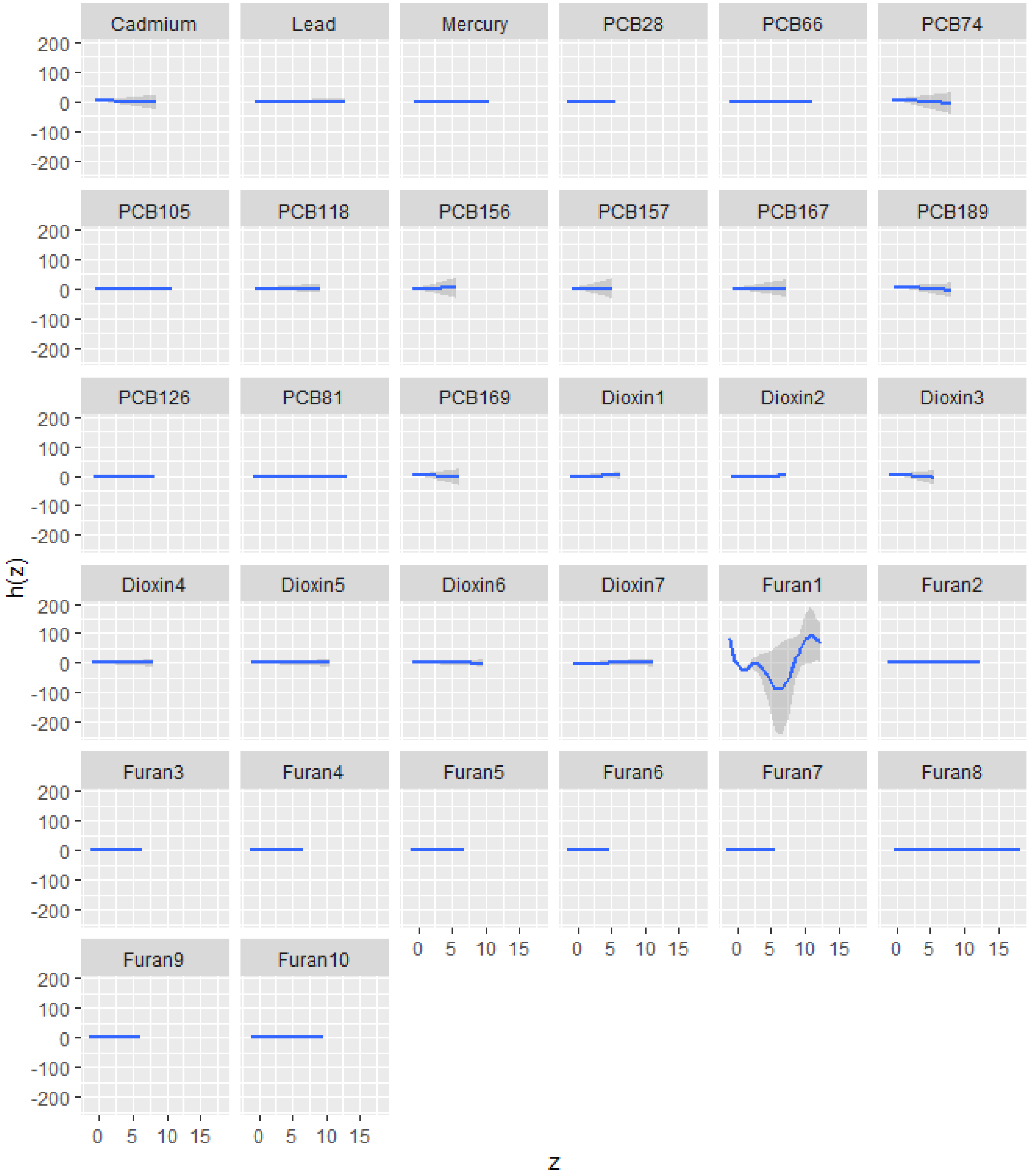

Supplementary Figure S12. Univariate exposure–response functions and 95% credible interval of metals, PCBs, Dioxins and Furans on CVD-related outcome: TC.

Triglycerides

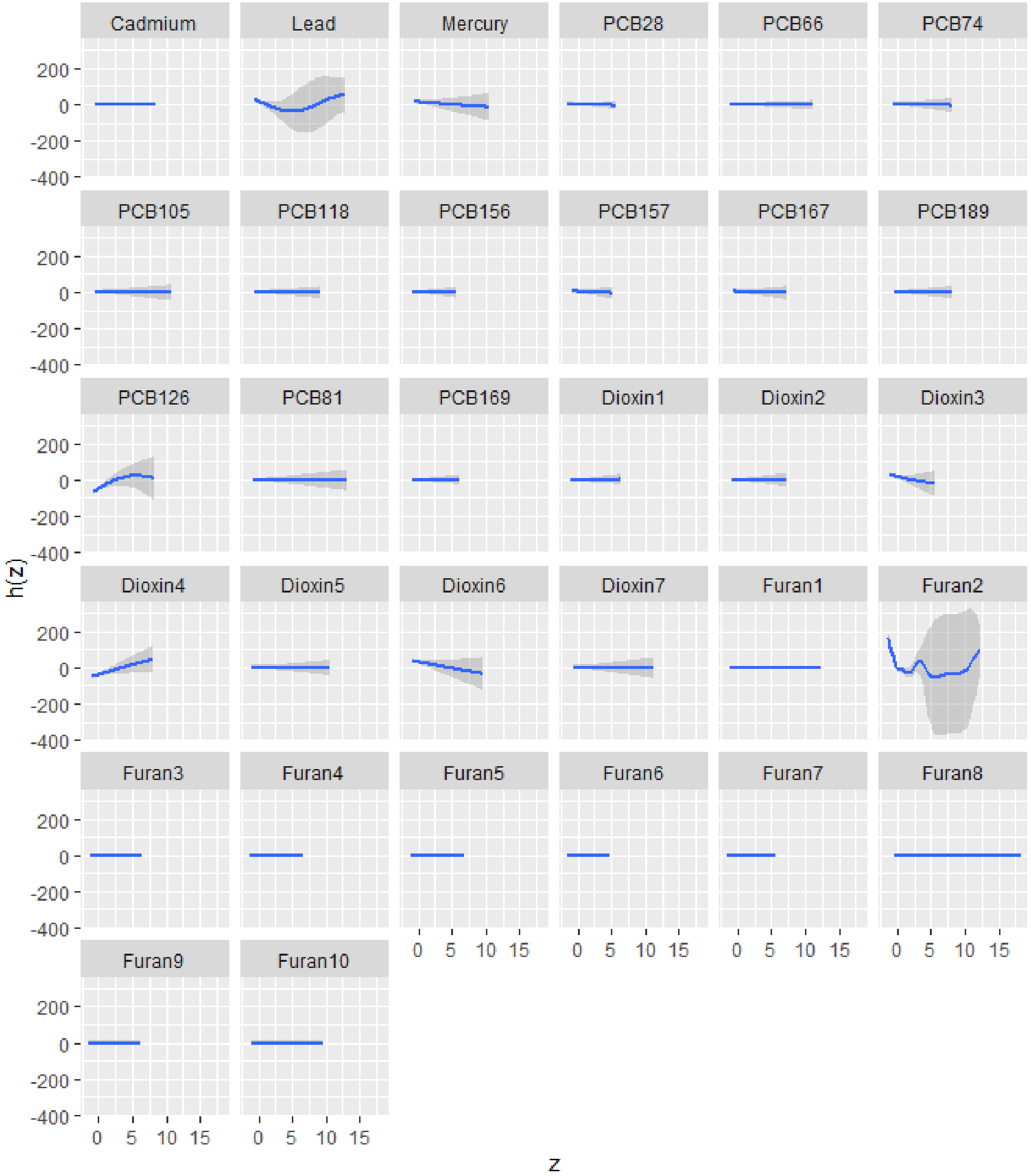

Supplementary Figure S13. Univariate exposure–response functions and 95% credible interval of metals, PCBs, Dioxins and Furans on CVD-related outcome: Triglycerides.

Overall exposure effects for DBP, SBP, HDL, LDL, TC, and Triglycerides are shown in the figures below.

DBP

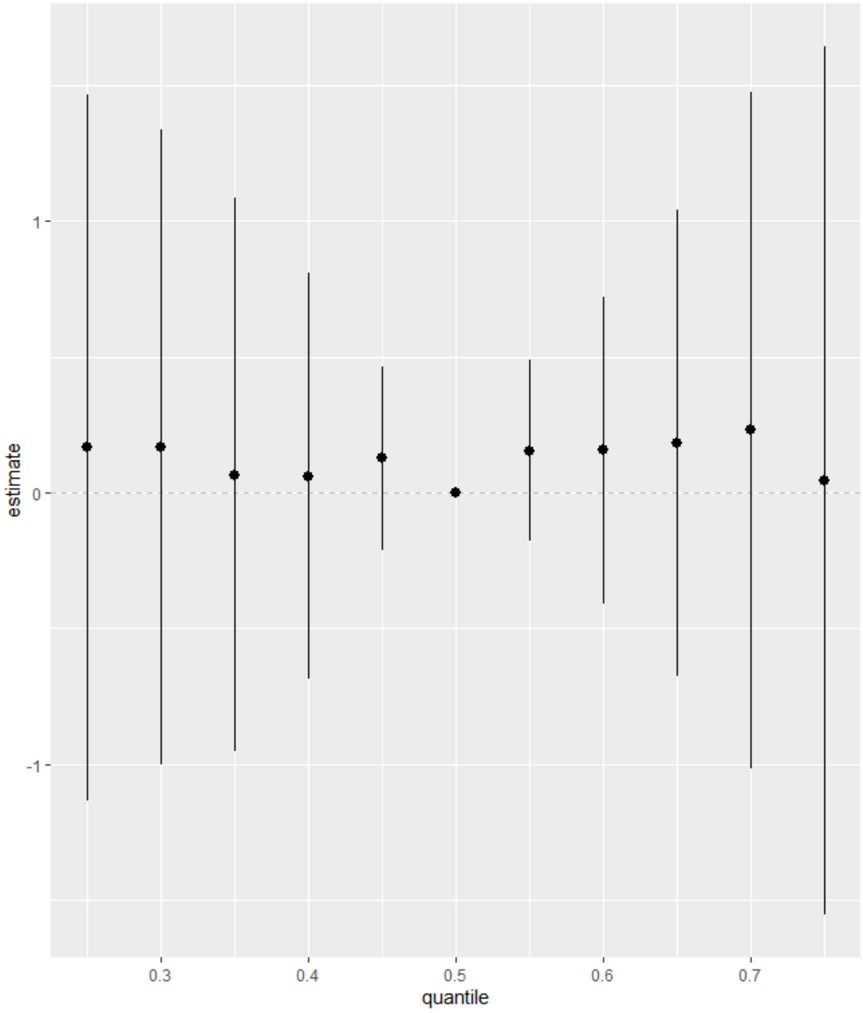

Supplementary Figure S14. Overall exposure effects – Chart showing the increasing quantile of combined exposures from 25th to 75th quantile as compared to the 50th quantile for DBP. Adjusted for alcohol consumption, smoking status, age, ethnicity, income level, gender, and BMI.

SBP

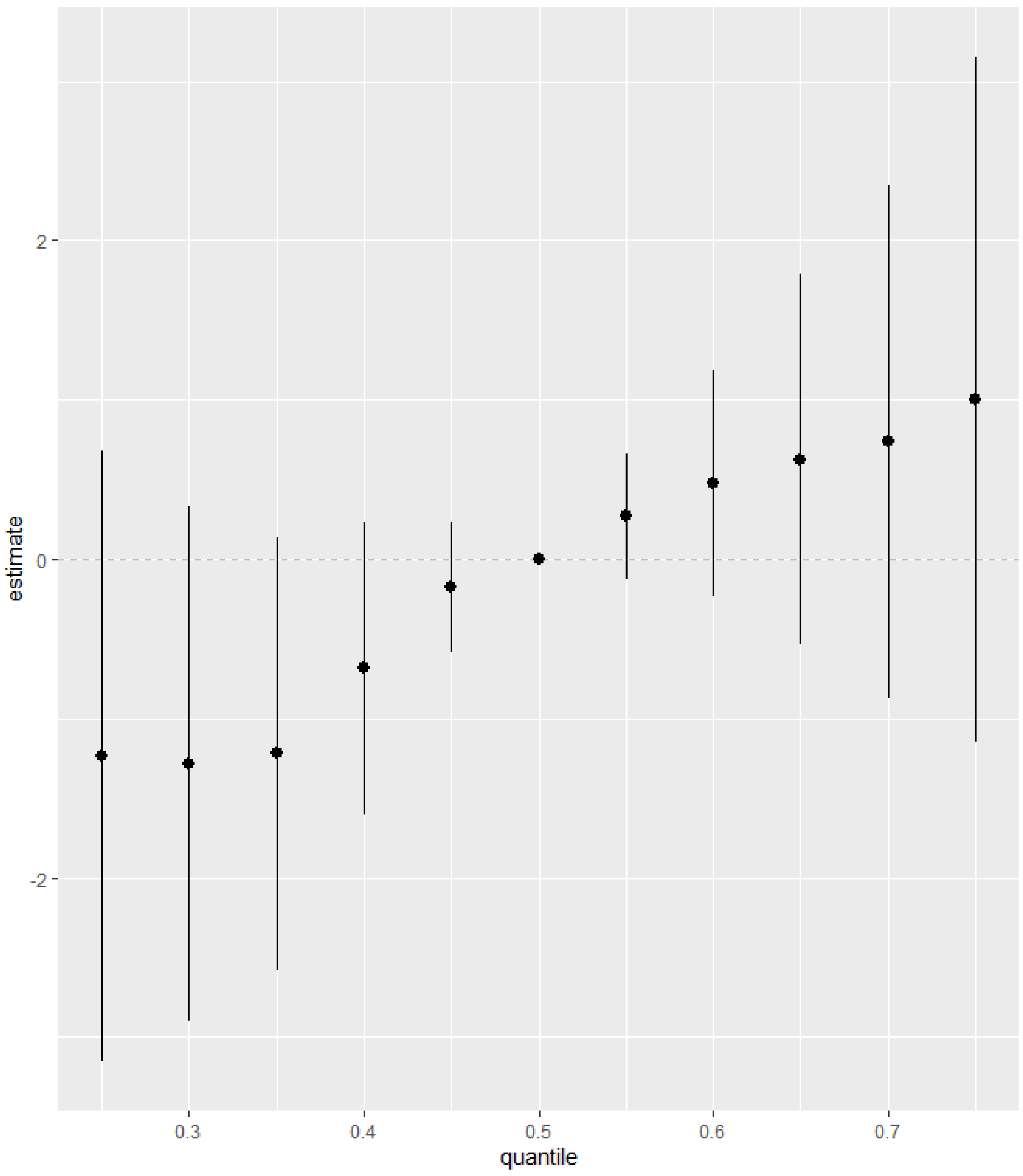

Supplementary Figure S15. Overall exposure effects – Chart showing the increasing quantile of combined exposures from 25th to 75th quantile as compared to the 50th quantile for SBP. Adjusted for alcohol consumption, smoking status, age, ethnicity, income level, gender, and BMI.

HDL Cholesterol

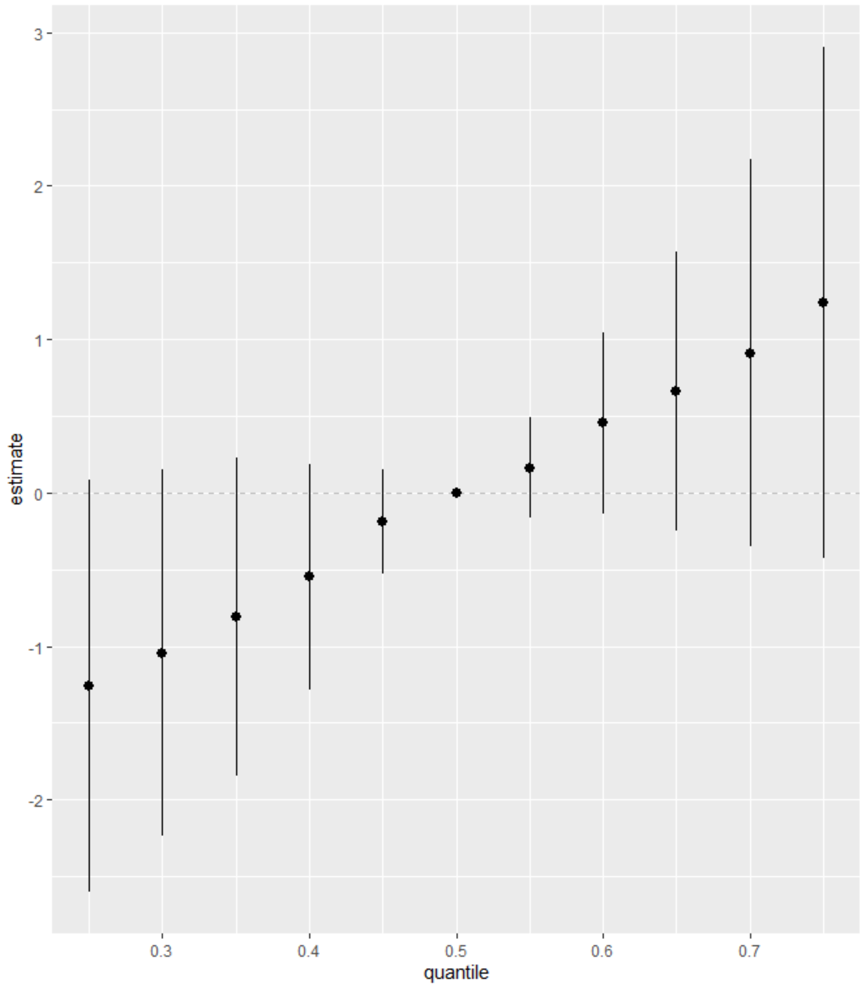

Supplementary Figure S16. Overall exposure effects – Chart showing the increasing quantile of combined exposures from 25th to 75th quantile as compared to the 50th quantile for HDL. Adjusted for alcohol consumption, smoking status, age, ethnicity, income level, gender, and BMI.

LDL Cholesterol

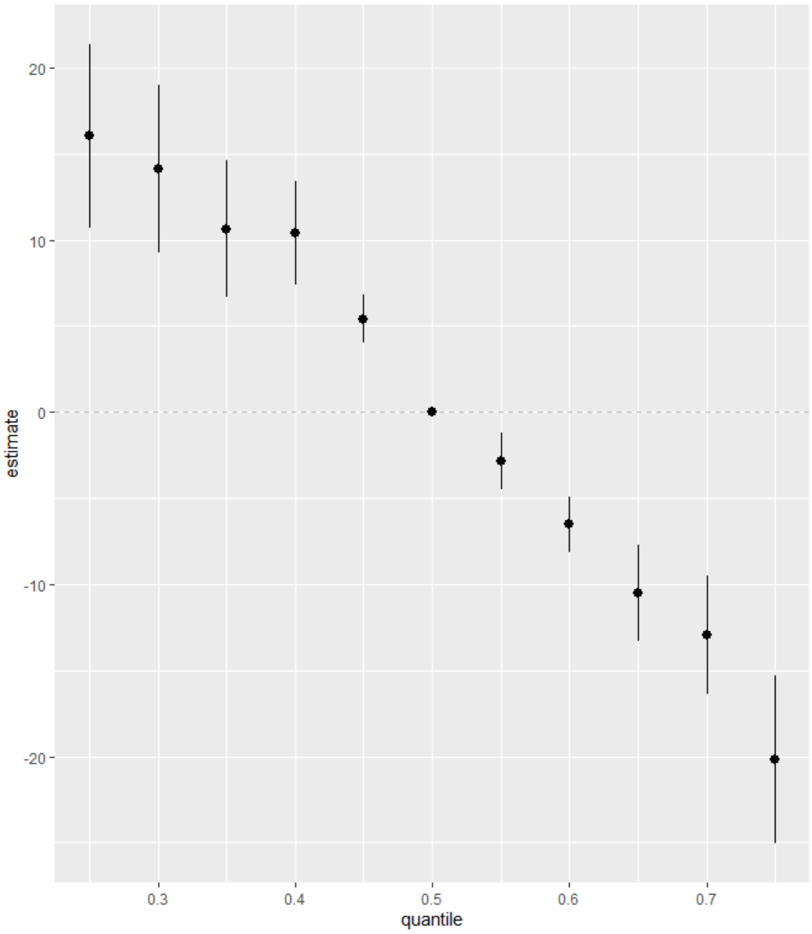

Supplementary Figure S17. Overall exposure effects – Chart showing the increasing quantile of combined exposures from 25th to 75th quantile as compared to the 50th quantile for LDL. Adjusted for alcohol consumption, smoking status, age, ethnicity, income level, gender, and BMI.

TC

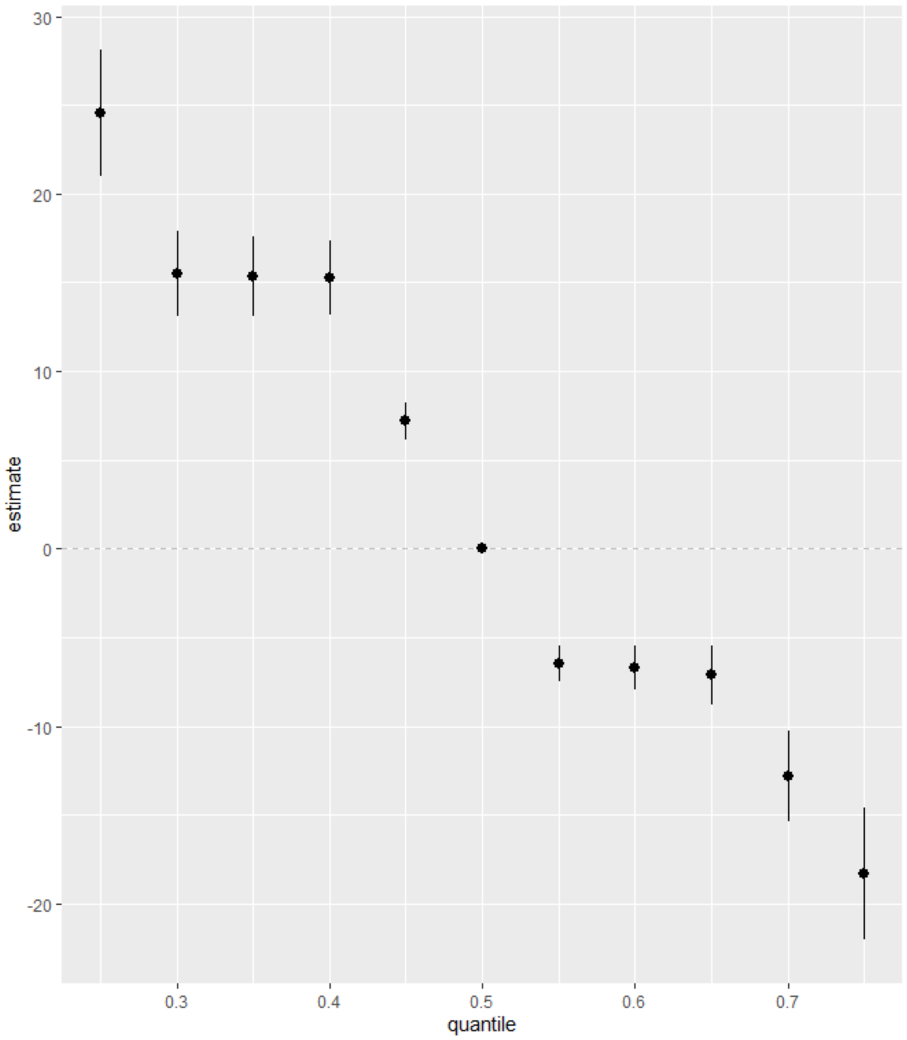

Supplementary Figure S18. Overall exposure effects – Chart showing the increasing quantile of combined exposures from 25th to 75th quantile as compared to the 50th quantile for TC. Adjusted for alcohol consumption, smoking status, age, ethnicity, income level, gender, and BMI.

Triglycerides

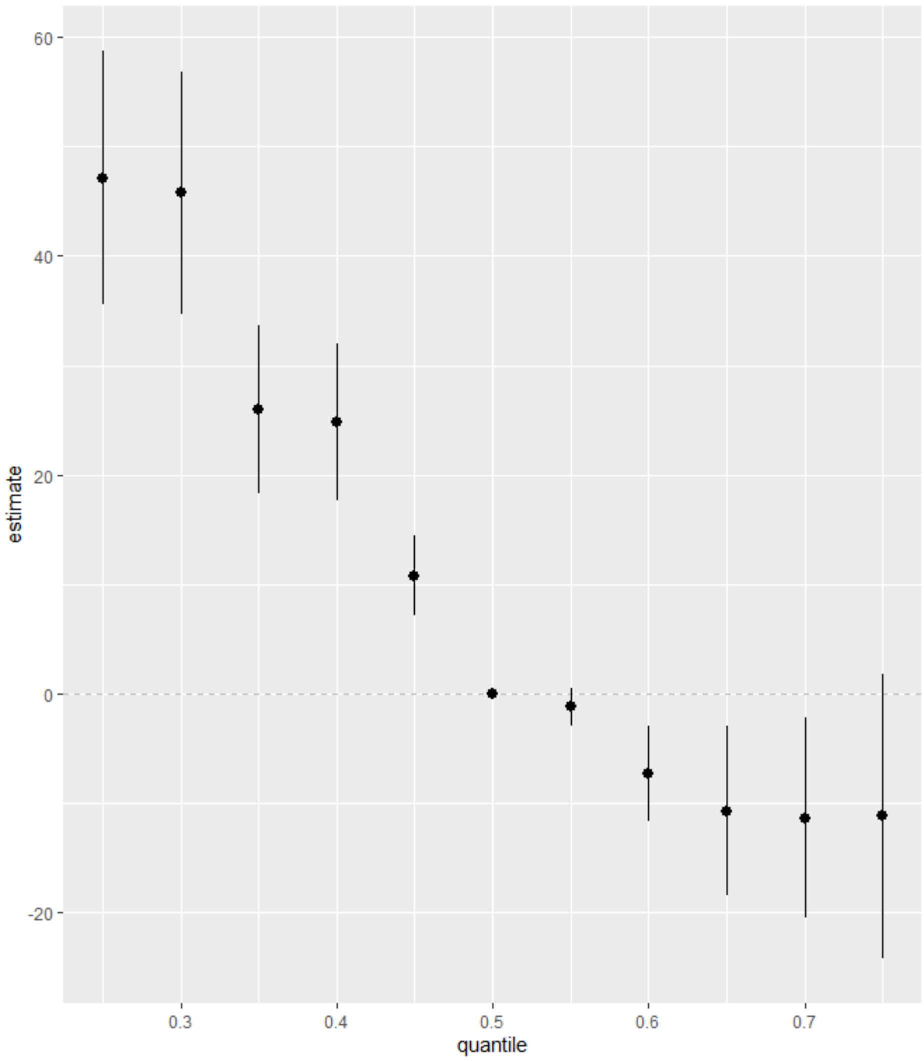

Supplementary Figure S19. Overall exposure effects – Chart showing the increasing quantile of combined exposures from 25th to 75th quantile as compared to the 50th quantile for Triglycerides. Adjusted for alcohol consumption, smoking status, age, ethnicity, income level, gender, and BMI.

Single Variable Effect

Charts below showing the single-exposure effect and 95% credible interval, defined as the change in response associated with a change in a single exposure from its 25th to 75th quantile while all other exposures are fixed at a specific quantile (25th, 50th, or 75th). Exposures of interest are metals, PCBs, Dioxins and Furans. DBP, SBP, FRS, HDL, LDL Cholesterol, TC, Triglycerides. Adjusted for alcohol consumption, smoking status, age, ethnicity, income level, gender, and BMI.

DBP

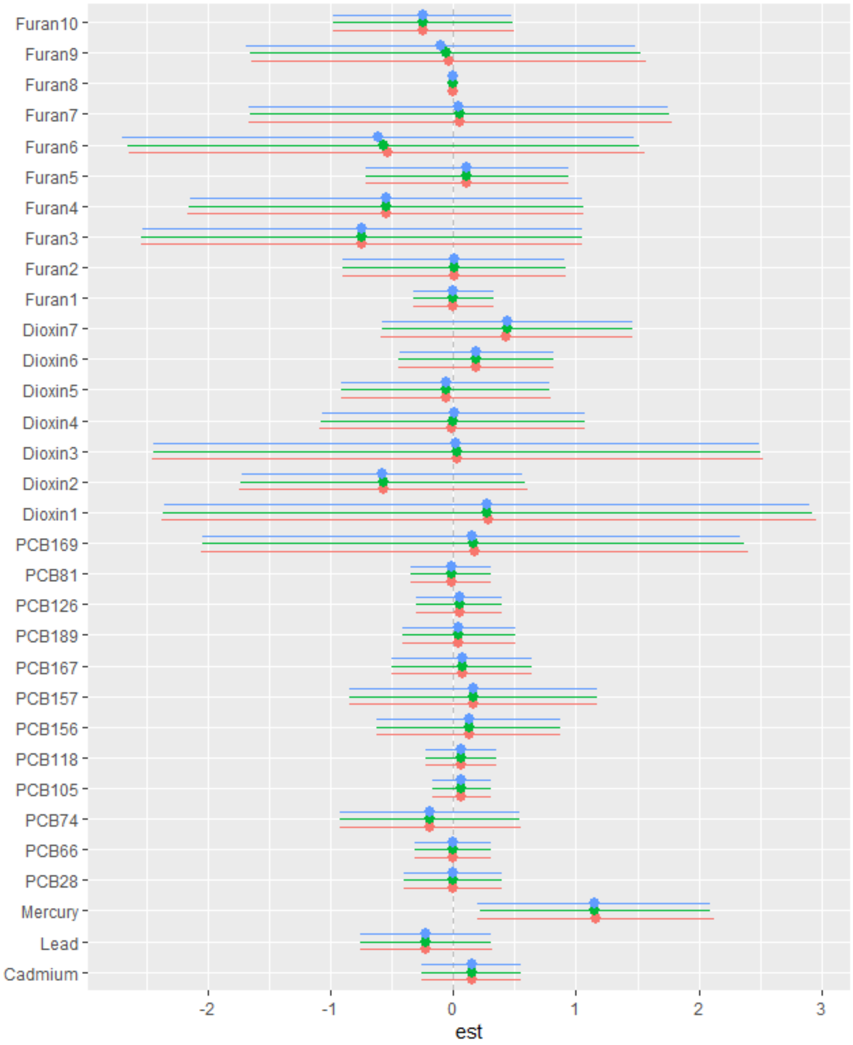

Supplementary Figure S20. Single Variable Effect - Chart showing the single-exposure effect and 95% credible interval, defined as the change in response associated with a change in a single exposure from its 25th to 75th quantile while all other exposures are fixed at a specific quantile (25th, 50th, or 75th) for DBP. Adjusted for alcohol consumption, smoking status, age, ethnicity, income level, gender, and BMI.

SBP

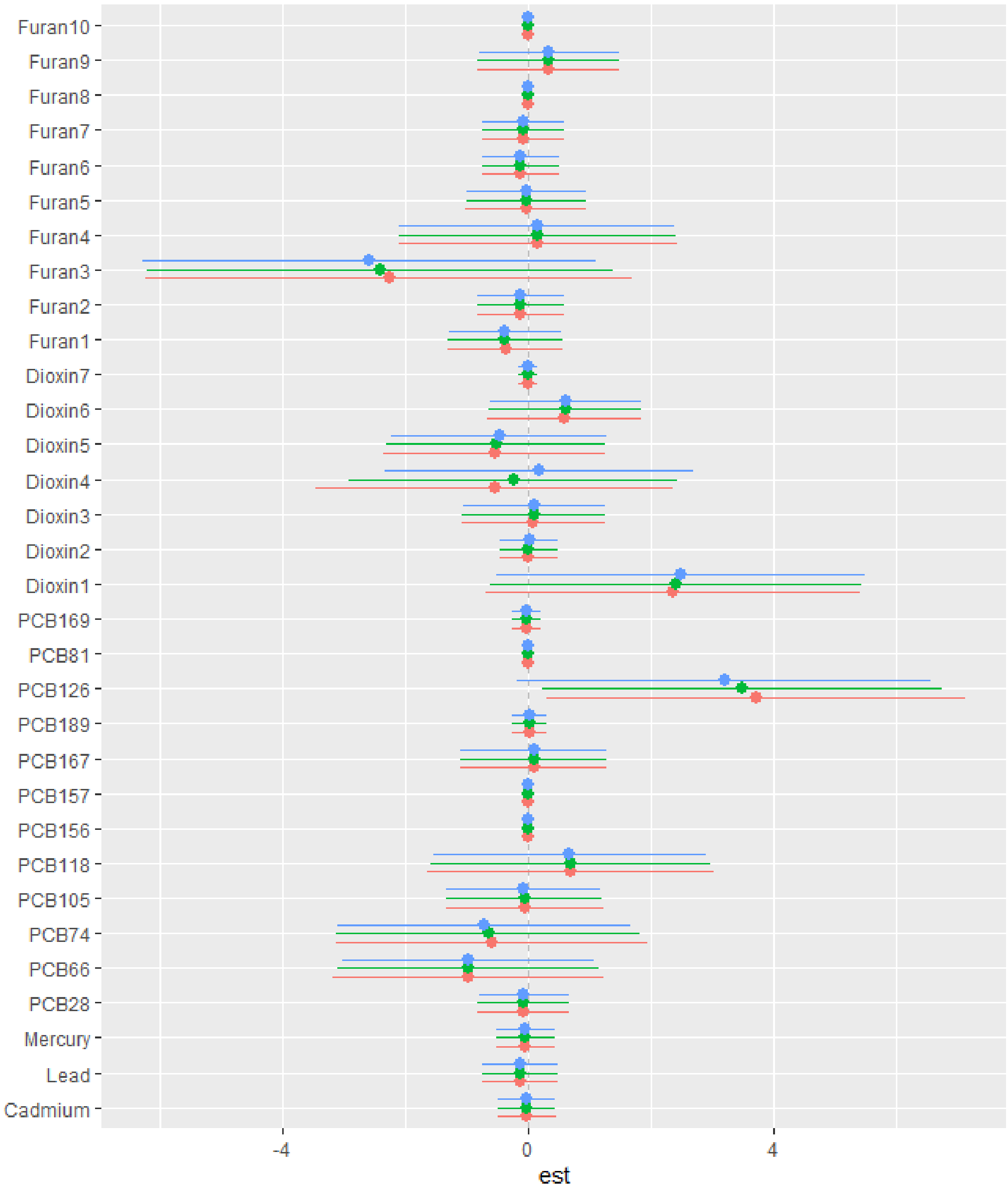

Supplementary Figure S21. Single Variable Effect - Chart showing the single-exposure effect and 95% credible interval, defined as the change in response associated with a change in a single exposure from its 25th to 75th quantile while all other exposures are fixed at a specific quantile (25th, 50th, or 75th) for SBP. Adjusted for alcohol consumption, smoking status, age, ethnicity, income level, gender, and BMI.

HDL Cholesterol

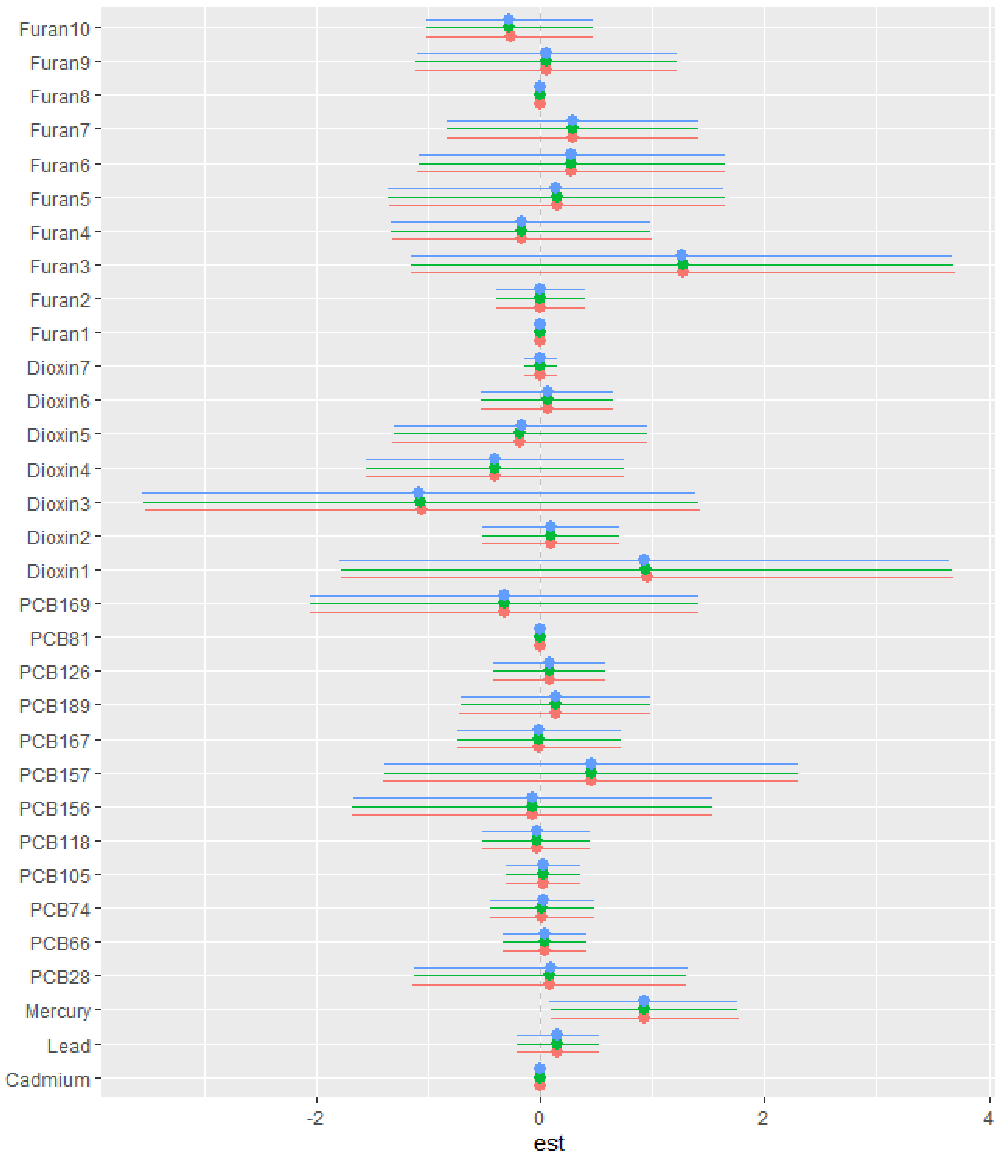

Supplementary Figure S22. Single Variable Effect - Chart showing the single-exposure effect and 95% credible interval, defined as the change in response associated with a change in a single exposure from its 25th to 75th quantile while all other exposures are fixed at a specific quantile (25th, 50th, or 75th) for HDL. Adjusted for alcohol consumption, smoking status, age, ethnicity, income level, gender, and BMI.

LDL Cholesterol

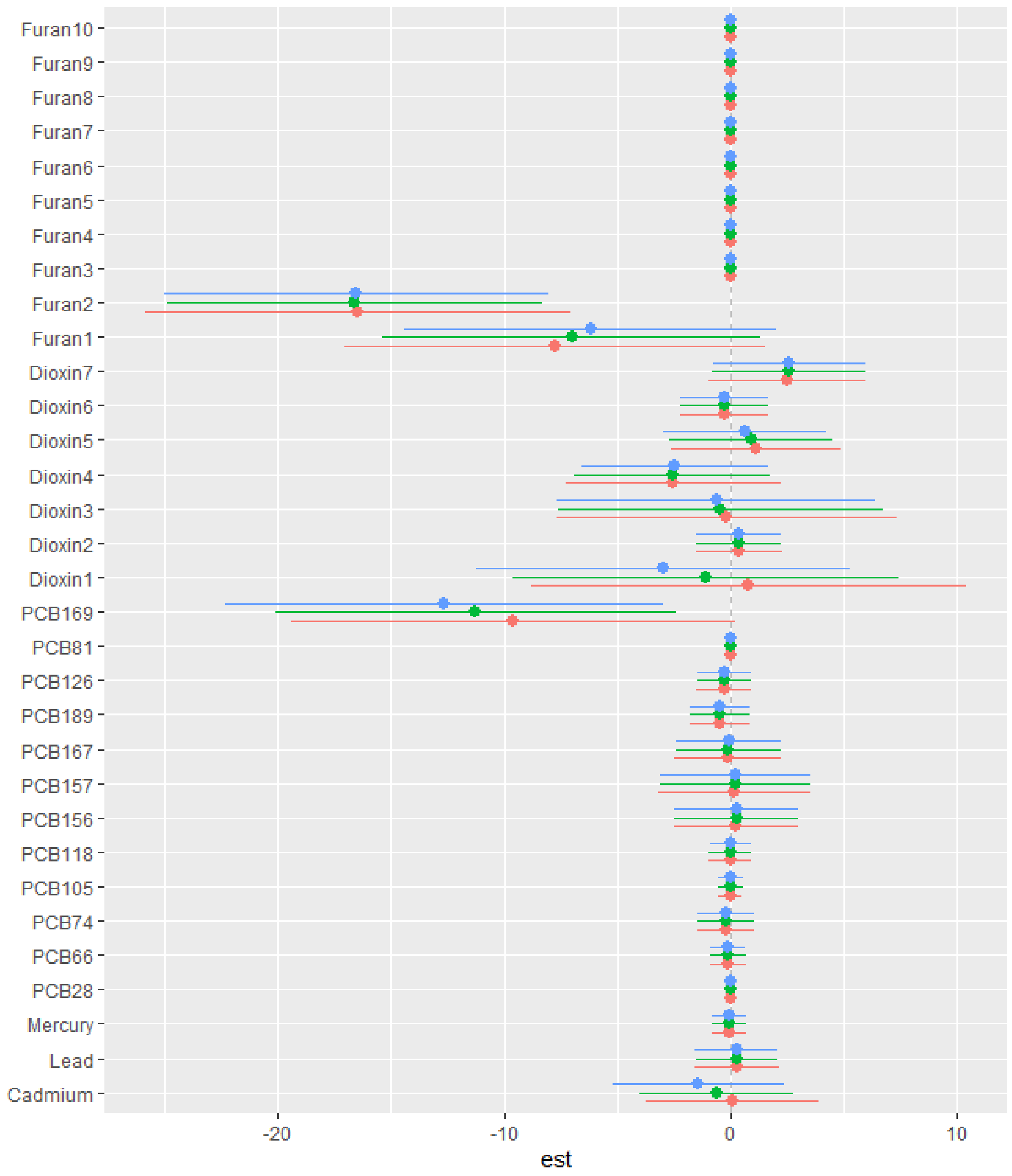

Supplementary Figure S23. Single Variable Effect - Chart showing the single-exposure effect and 95% credible interval, defined as the change in response associated with a change in a single exposure from its 25th to 75th quantile while all other exposures are fixed at a specific quantile (25th, 50th, or 75th) for LDL. Adjusted for alcohol consumption, smoking status, age, ethnicity, income level, gender, and BMI.

TC

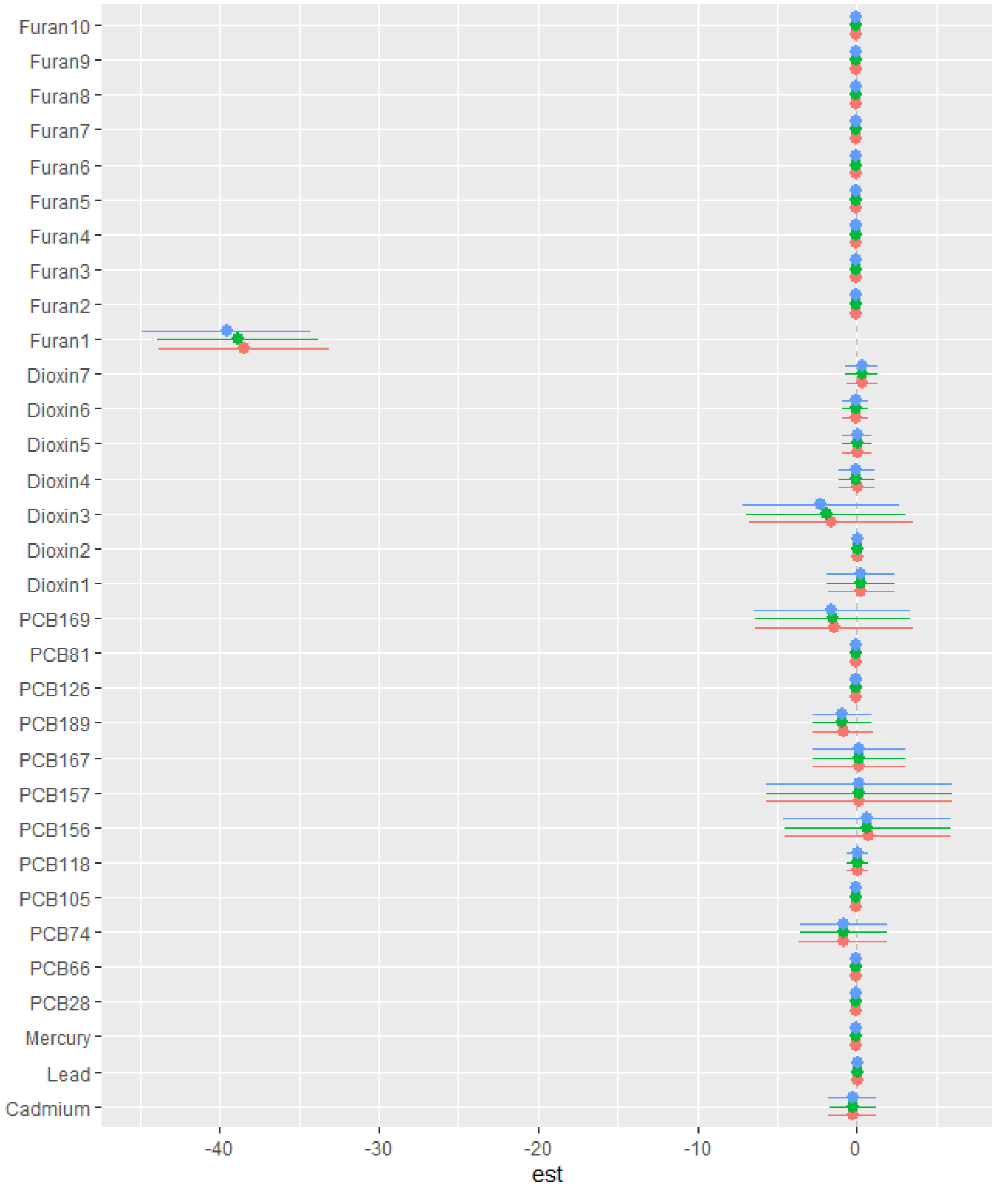

Supplementary Figure S24. Single Variable Effect - Chart showing the single-exposure effect and 95% credible interval, defined as the change in response associated with a change in a single exposure from its 25th to 75th quantile while all other exposures are fixed at a specific quantile (25th, 50th, or 75th) for TC. Adjusted for alcohol consumption, smoking status, age, ethnicity, income level, gender, and BMI.

Triglyerides

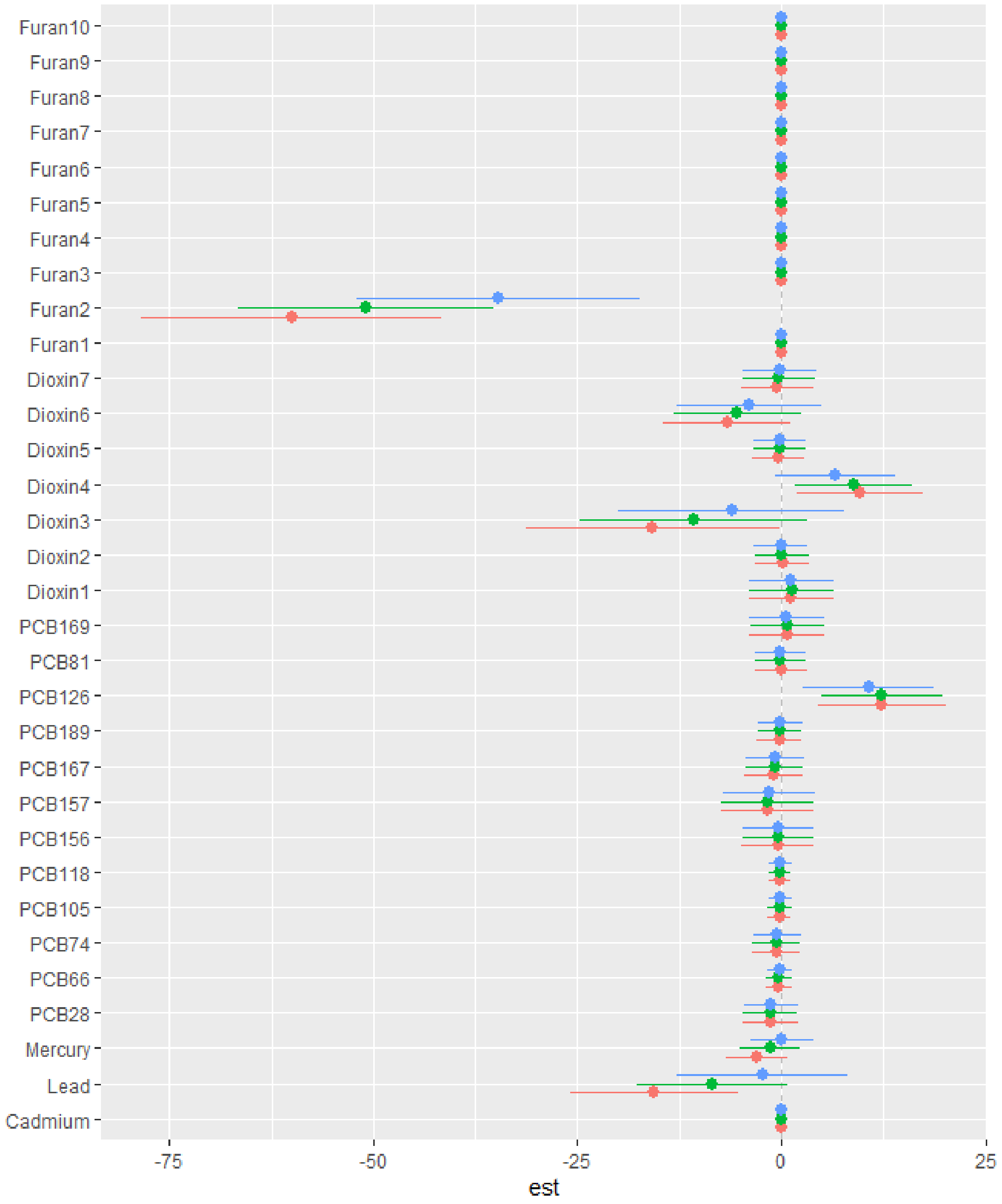

Supplementary Figure S25. Single Variable Effect - Chart showing the single-exposure effect and 95% credible interval, defined as the change in response associated with a change in a single exposure from its 25th to 75th quantile while all other exposures are fixed at a specific quantile (25th, 50th, or 75th) for TC. Adjusted for alcohol consumption, smoking status, age, ethnicity, income level, gender, and BMI.

Single Variable Interaction analysis is shown in supplementary S26–S31 below.

DBP

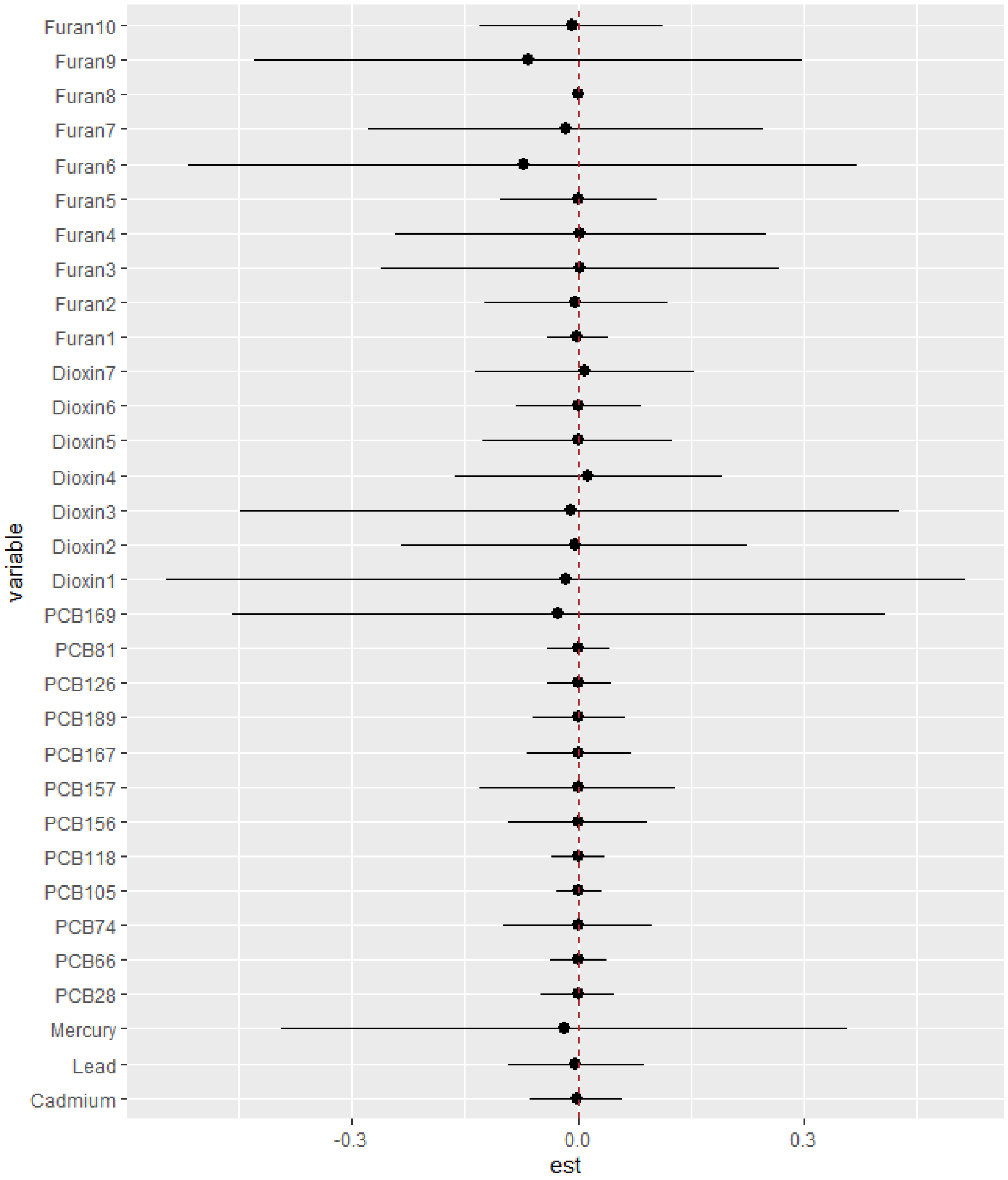

Supplementary Figure S26. Single-variable interaction analyses for FRS assessing how the effect of each environmental exposure (metals, PCBs, dioxins, and furans), from its 25th to 75th percentile, when all others are fixed at the 25th percentile compared to the 75th percentile. Adjusted for alcohol consumption, smoking status, age, ethnicity, income level, gender, and BMI.

SBP

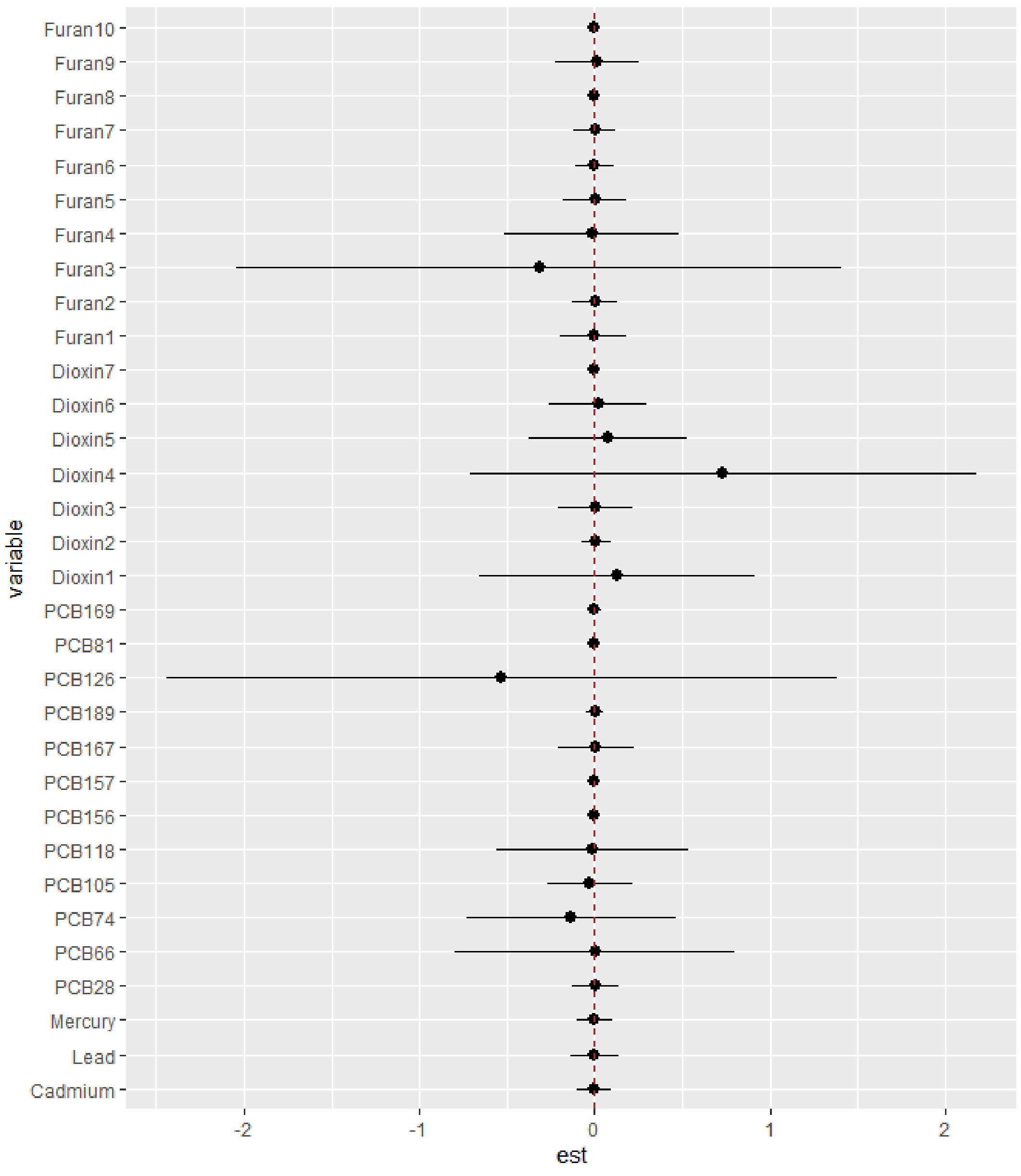

Supplementary Figure S27. Single-variable interaction analyses for SBP assessing how the effect of each environmental exposure (metals, PCBs, dioxins, and furans), from its 25th to 75th percentile, when all others are fixed at the 25th percentile compared to the 75th percentile. Adjusted for alcohol consumption, smoking status, age, ethnicity, income level, gender, and BMI.

HDL Cholesterol

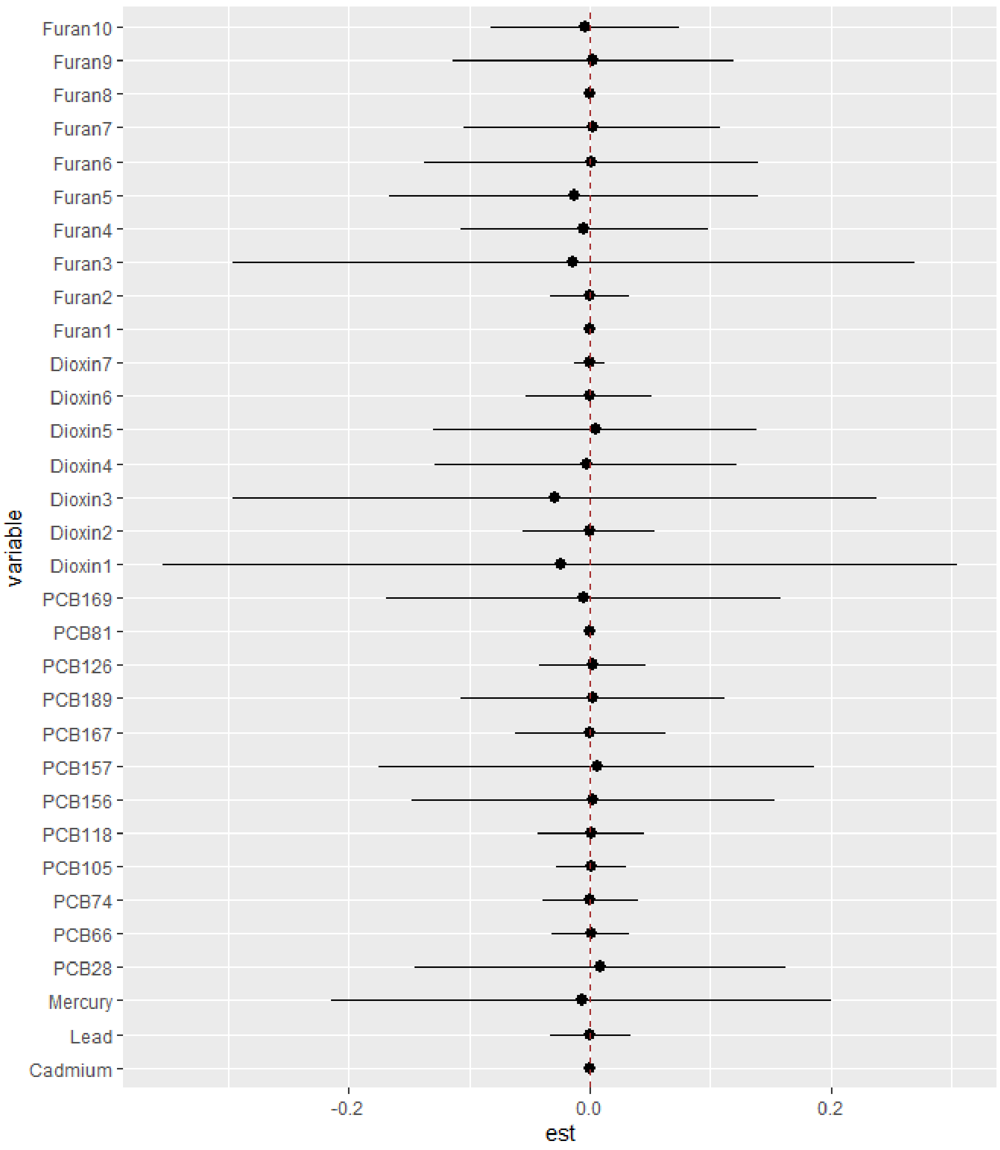

Supplementary Figure S28. Single-variable interaction analyses for HDL assessing how the effect of each environmental exposure (metals, PCBs, dioxins, and furans), from its 25th to 75th percentile, when all others are fixed at the 25th percentile compared to the 75th percentile. Adjusted for alcohol consumption, smoking status, age, ethnicity, income level, gender, and BMI.

LDL Cholesterol

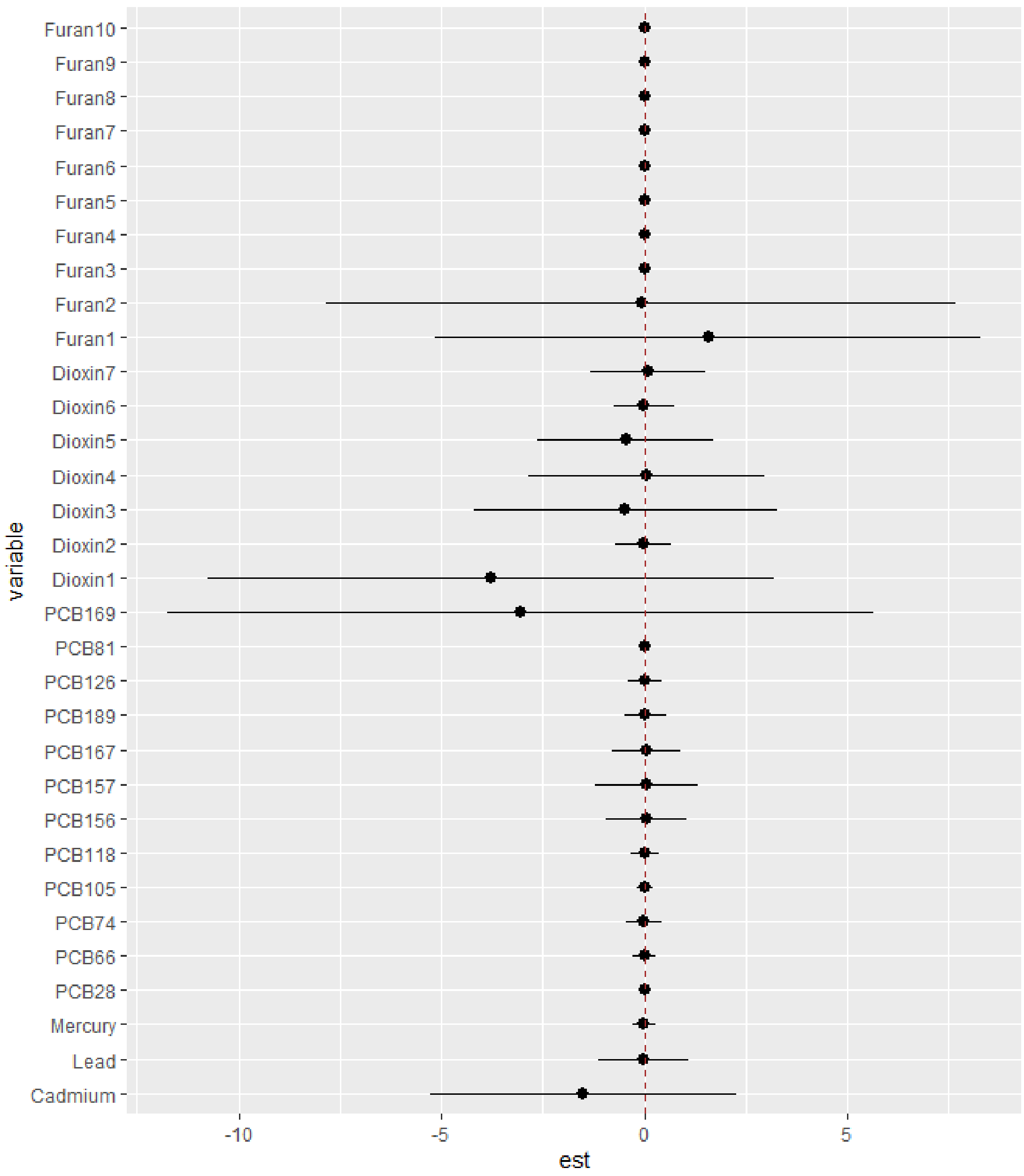

Supplementary Figure S29. Single-variable interaction analyses for LDL assessing how the effect of each environmental exposure (metals, PCBs, dioxins, and furans), from its 25th to 75th percentile, when all others are fixed at the 25th percentile compared to the 75th percentile. Adjusted for alcohol consumption, smoking status, age, ethnicity, income level, gender, and BMI.

TC

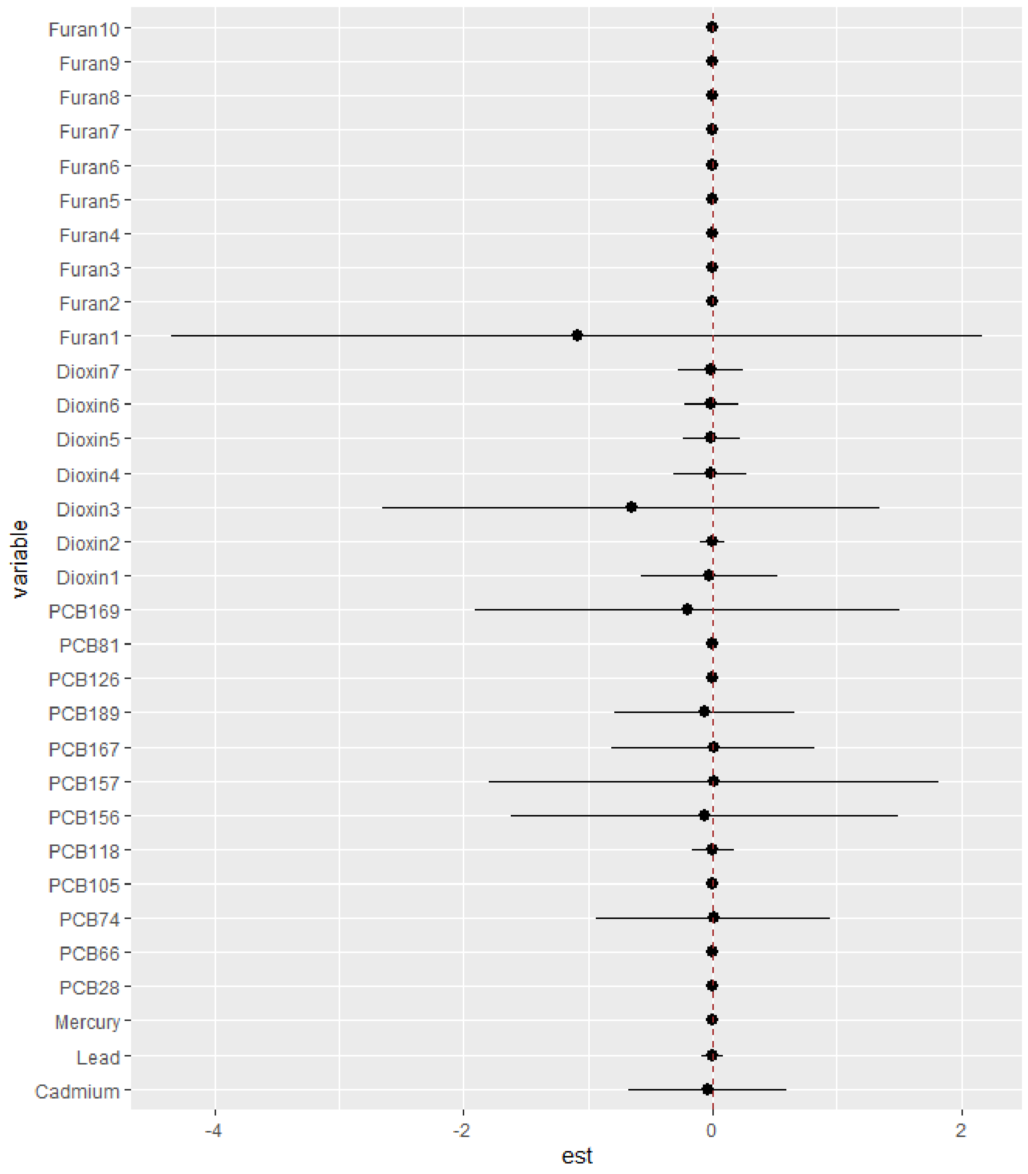

Supplementary Figure S30. Single-variable interaction analyses for TC assessing how the effect of each environmental exposure (metals, PCBs, dioxins, and furans), from its 25th to 75th percentile, when all others are fixed at the 25th percentile compared to the 75th percentile. Adjusted for alcohol consumption, smoking status, age, ethnicity, income level, gender, and BMI.

Triglycerides

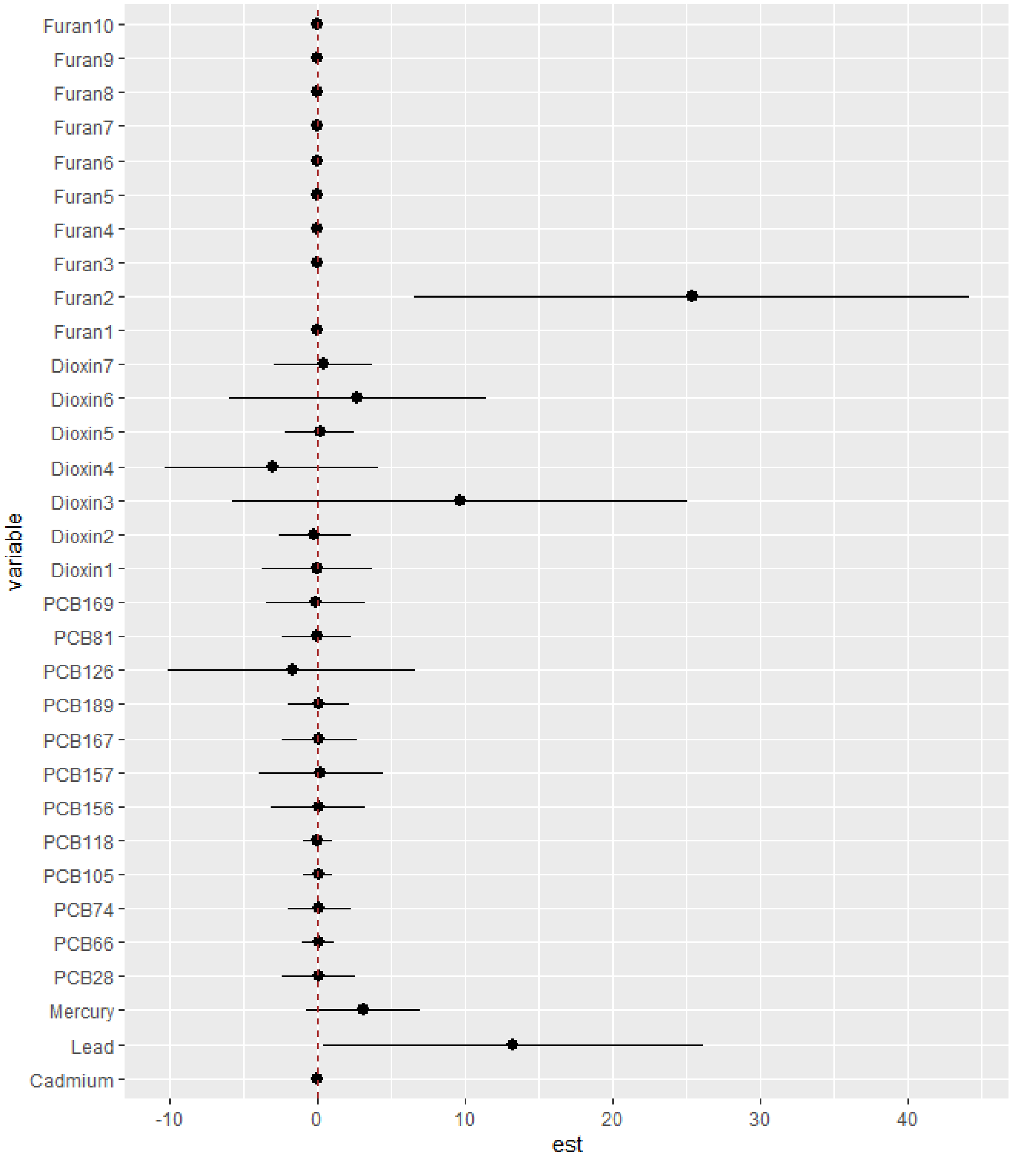

Supplementary Figure S31. Single-variable interaction analyses for triglycerides assessing how the effect of each environmental exposure (metals, PCBs, dioxins, and furans), from its 25th to 75th percentile, when all others are fixed at the 25th percentile compared to the 75th percentile. Adjusted for alcohol consumption, smoking status, age, ethnicity, income level, gender, and BMI.

The bivariate exposure-response function results are shown in Supplementary Figures S32-S37.

DBP

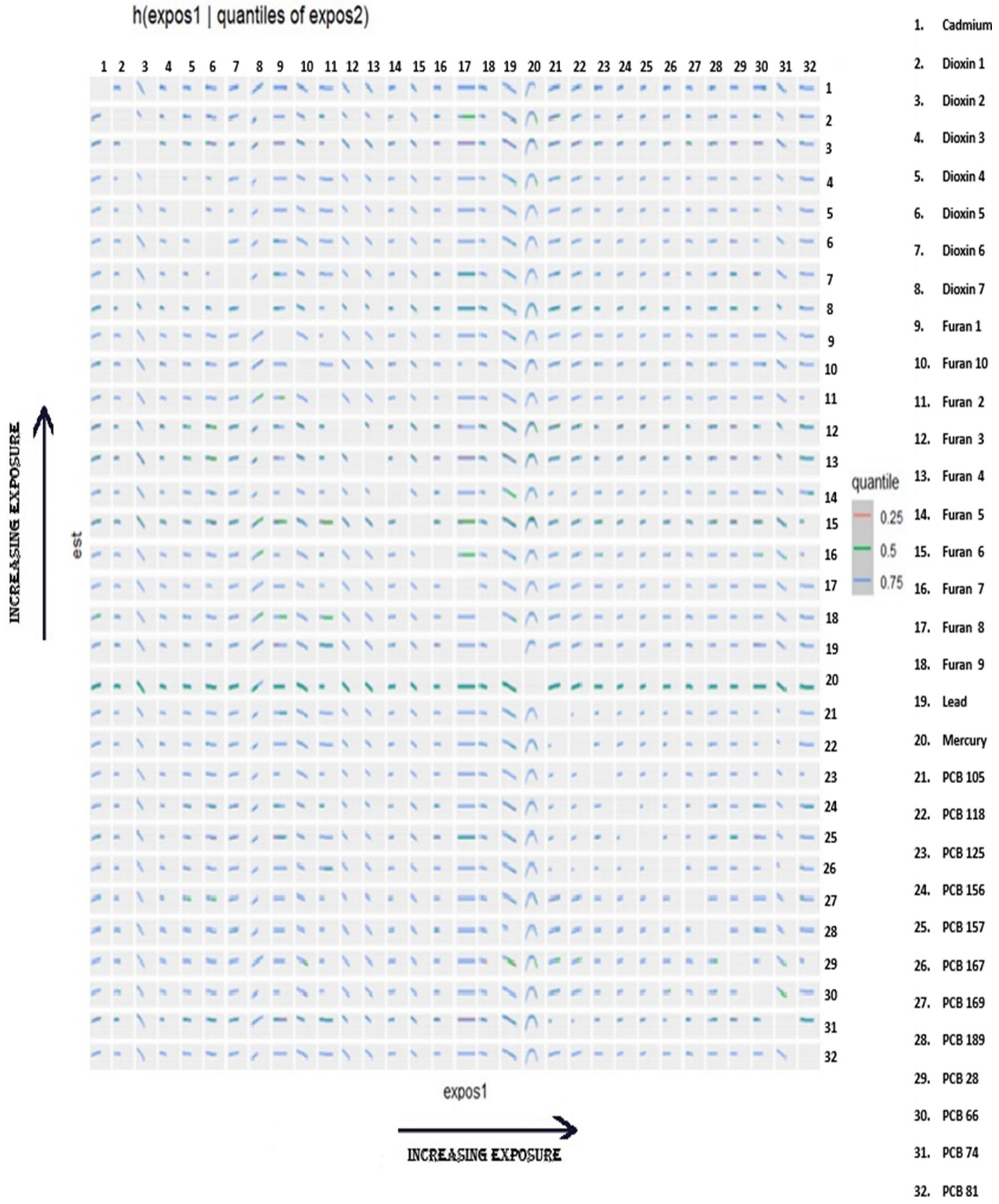

Supplementary Figure 32. Bivariate exposure response relationship for DBP- The bivariate exposure response function illustrates the joint association of increasing exposure of Metals, PCBs, Dioxins, and Furans (X-axis) as compared to increasing quantiles of a second exposure at the 0.25 (red), 0.5 (green), and 0.75 (blue) quantiles. Adjusted for alcohol consumption, smoking status, age, ethnicity, income level, gender, and BMI.

SBP

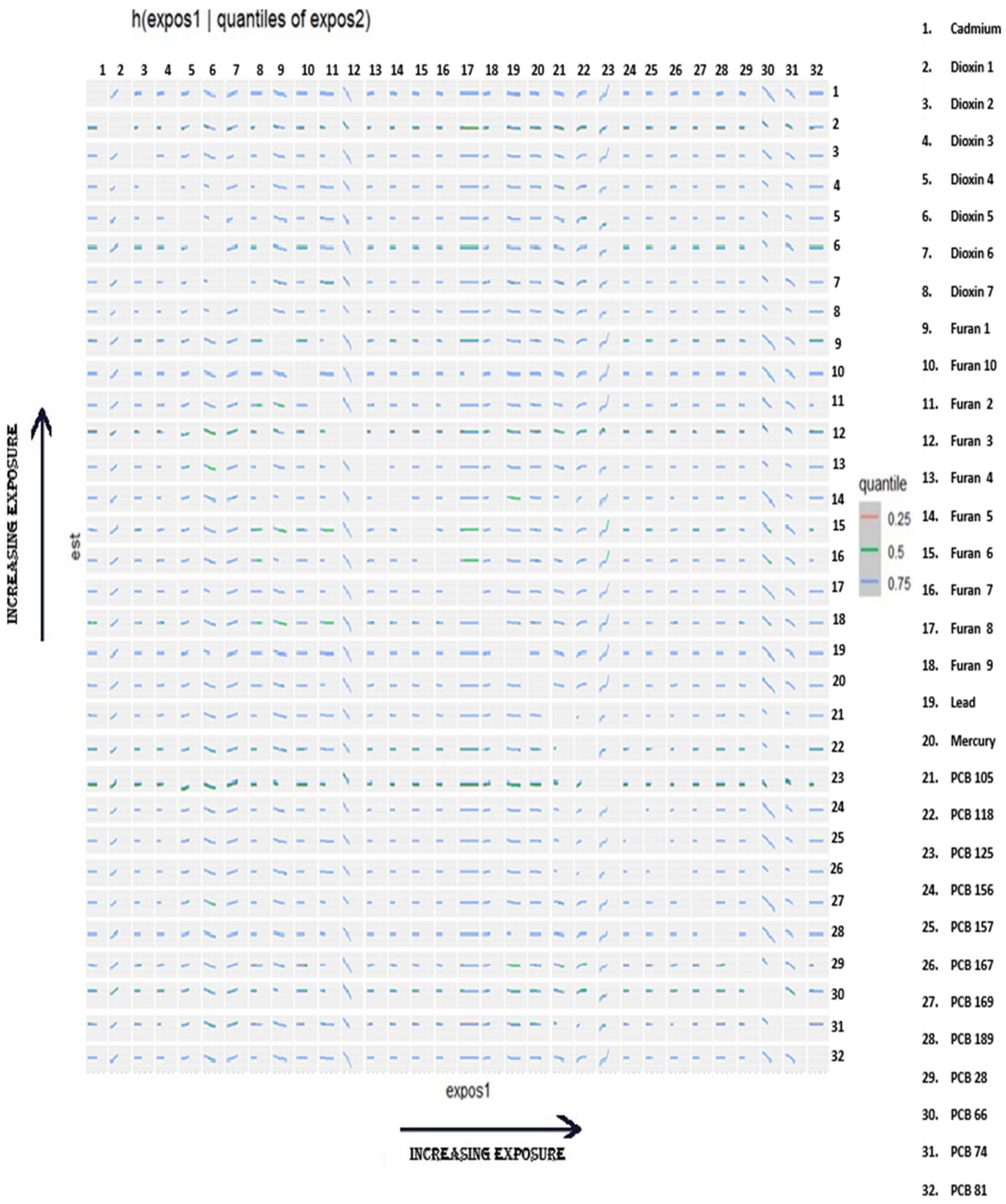

Supplementary Figure S33. Bivariate exposure response relationship for SBP- The bivariate exposure response function illustrates the joint association of increasing exposure of Metals, PCBs, Dioxins, and Furans (X-axis) as compared to increasing quantiles of a second exposure at the 0.25 (red), 0.5 (green), and 0.75 (blue) quantiles. Adjusted for alcohol consumption, smoking status, age, ethnicity, income level, gender, and BMI.

HDL

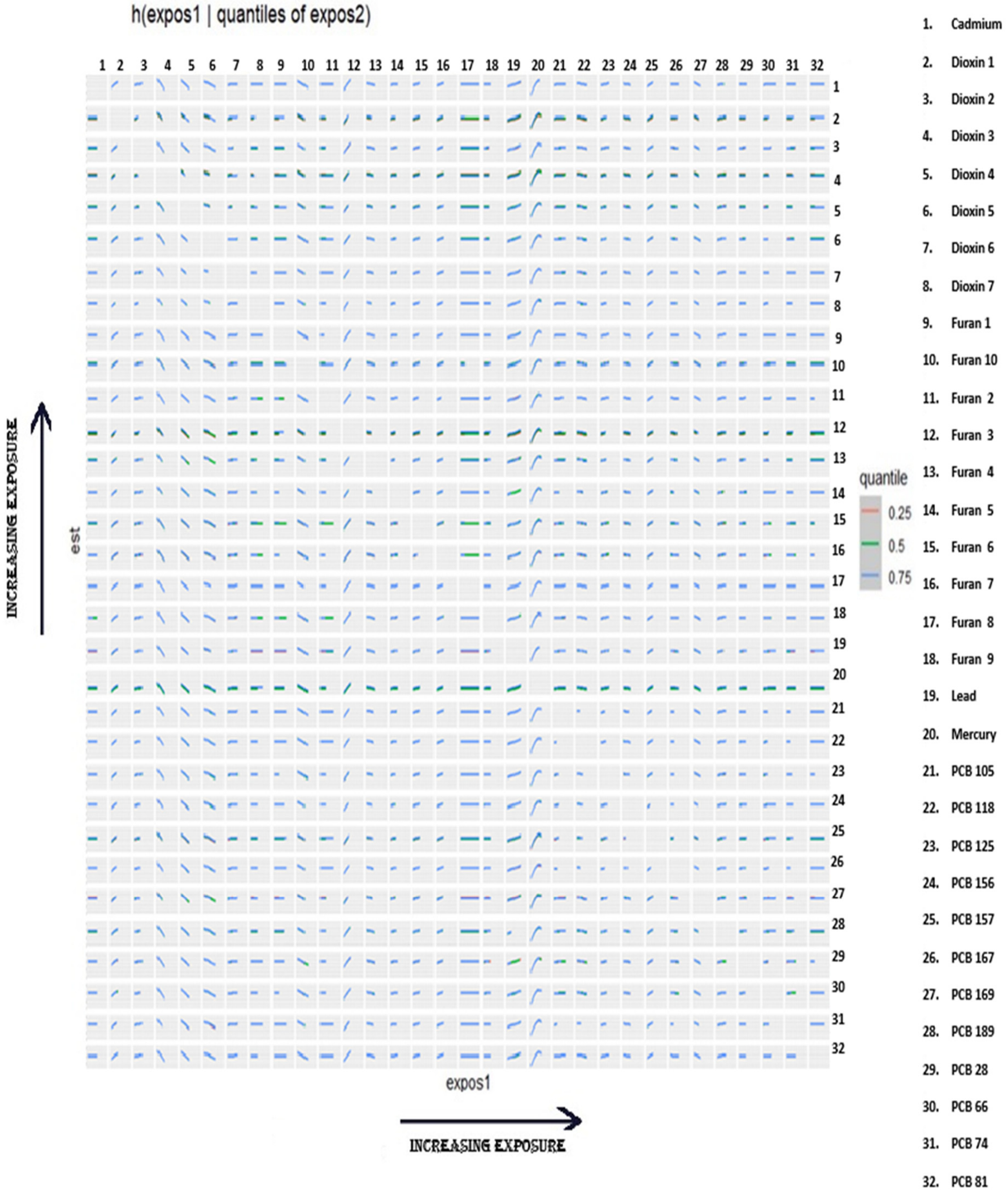

Supplementary Figure S34. Bivariate exposure response relationship for HDL- The bivariate exposure response function illustrates the joint association of increasing exposure of Metals, PCBs, Dioxins, and Furans (X-axis) as compared to increasing quantiles of a second exposure at the 0.25 (red), 0.5 (green), and 0.75 (blue) quantiles. Adjusted for alcohol consumption, smoking status, age, ethnicity, income level, gender, and BMI.

LDL Cholesterol

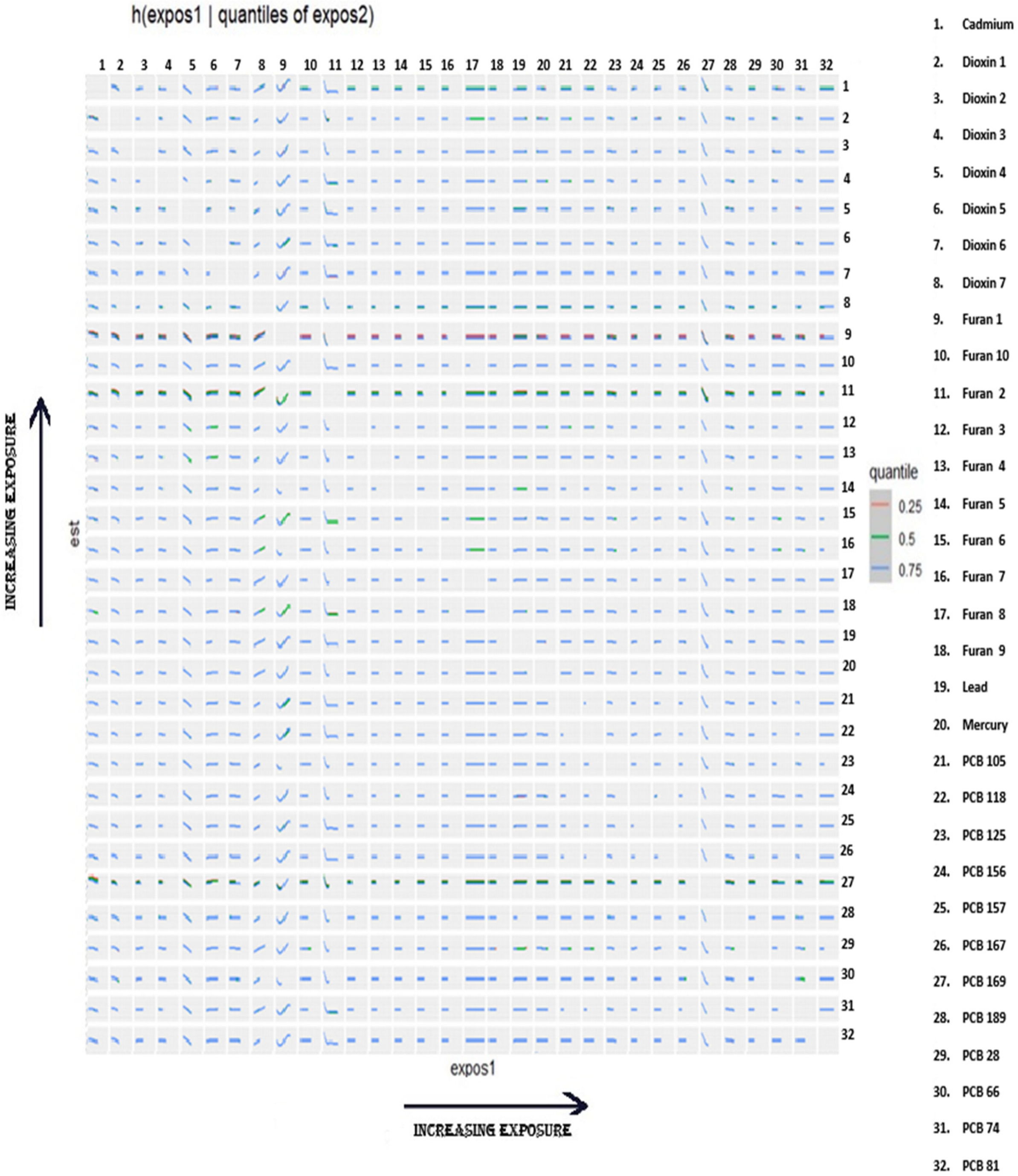

Supplementary Figure S35. Bivariate exposure response relationship for LDL- The bivariate exposure response function illustrates the joint association of increasing exposure of Metals, PCBs, Dioxins, and Furans (X-axis) as compared to increasing quantiles of a second exposure at the 0.25 (red), 0.5 (green), and 0.75 (blue) quantiles. Adjusted for alcohol consumption, smoking status, age, ethnicity, income level, gender, and BMI.

TC

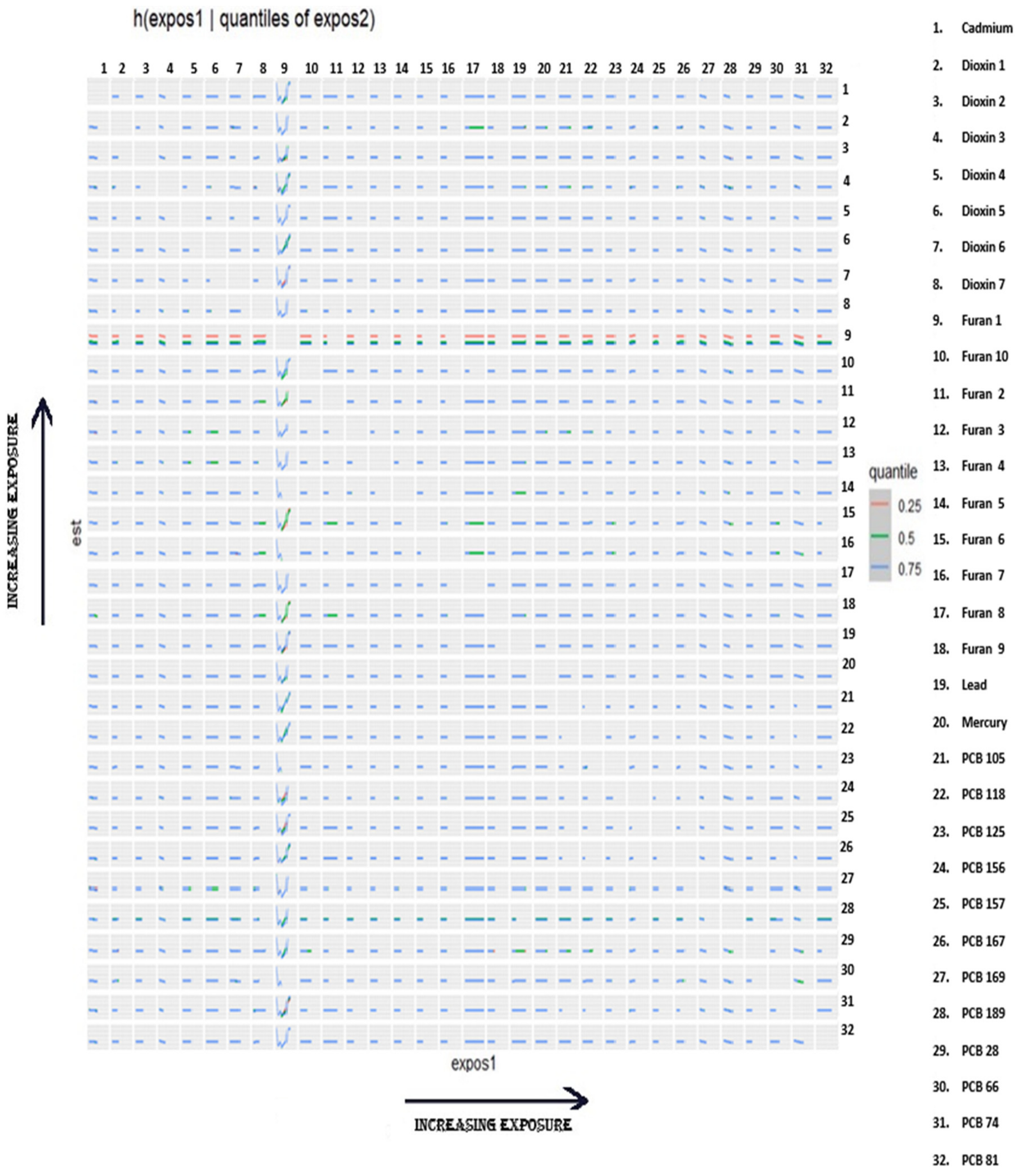

Supplementary Figure S36. Bivariate exposure response relationship for TC- The bivariate exposure response function illustrates the joint association of increasing exposure of Metals, PCBs, Dioxins, and Furans (X-axis) as compared to increasing quantiles of a second exposure at the 0.25 (red), 0.5 (green), and 0.75 (blue) quantiles. Adjusted for alcohol consumption, smoking status, age, ethnicity, income level, gender, and BMI.

Triglycerides

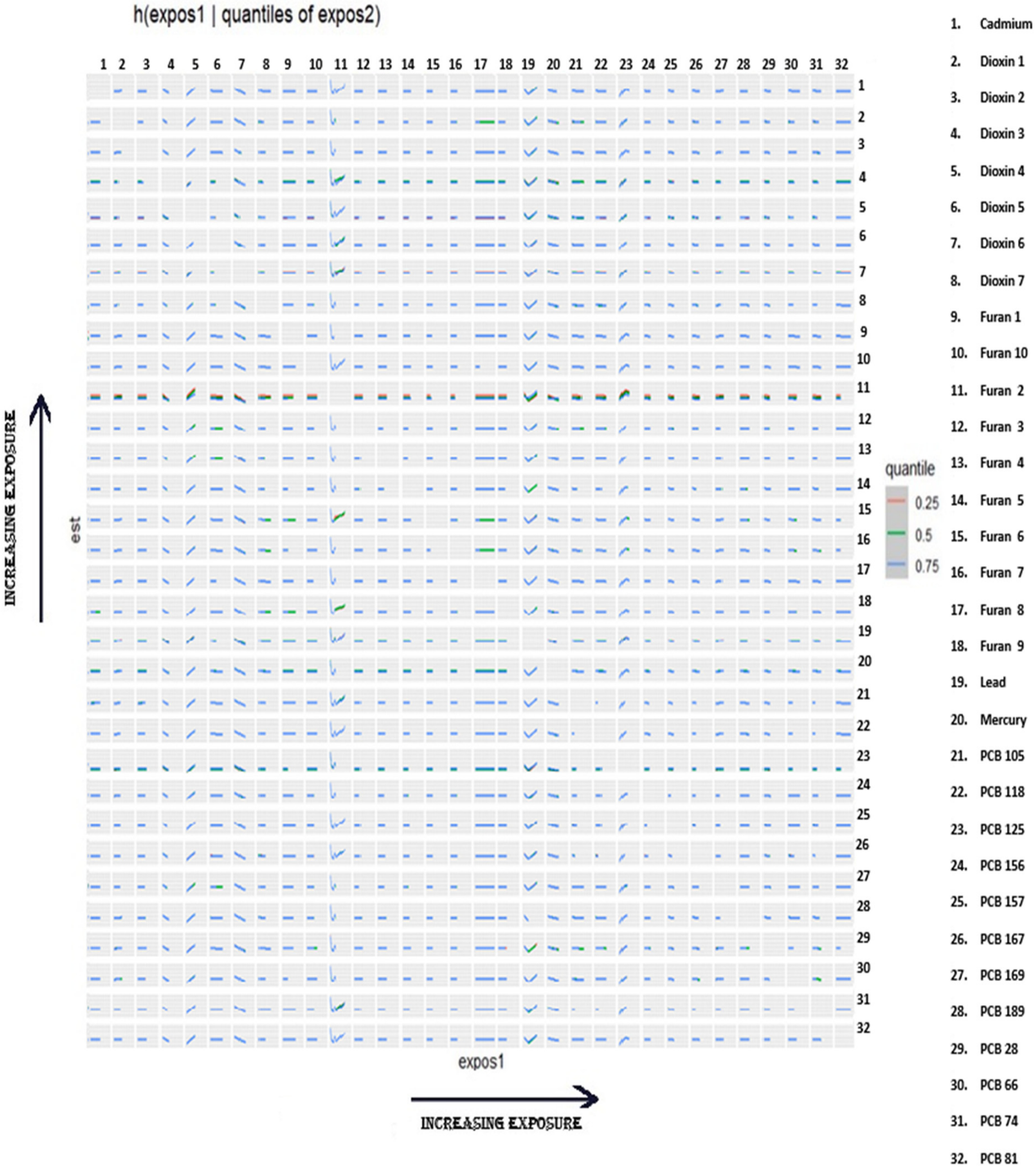

Supplementary Figure S37. Bivariate exposure response relationship for Triglycerides- The bivariate exposure response function illustrates the joint association of increasing exposure of Metals, PCBs, Dioxins, and Furans (X-axis) as compared to increasing quantiles of a second exposure at the 0.25 (red), 0.5 (green), and 0.75 (blue) quantiles. Adjusted for alcohol consumption, smoking status, age, ethnicity, income level, gender, and BMI.
